# Developments in the field of reliability and agreement studies in health, psychological and educational measurement: a methods review protocol

**DOI:** 10.1136/bmjopen-2025-109876

**Published:** 2026-07-22

**Authors:** M-M Pittelkow, M Mönch, L B Mokkink, D L Streiner, Stig Brorson, M Shoukri, J Kottner

**Affiliations:** 1 Institute of Clinical Nursing Science, Charité – University Medical Center Berlin, corporate member of Freie Universität Berlin and Humboldt-Universität zu Berlin, Berlin, Germany; 2 QUEST Center for Responsible Research, Berlin Institute of Health at Charité – Universitätsmedizin Berlin, Berlin, Germany; 3 Institute of Biometry and Clinical Epidemiology, Charité – University Medical Center Berlin, corporate member of Freie Universität Berlin and Humboldt-Universität zu Berlin, Berlin, Germany; 4 Department of Epidemiology and Data Science, Amsterdam UMC location Vrije Universiteit Amsterdam, Amsterdam, The Netherlands; 5 Methodology, Amsterdam Public Health Research Institute, Amsterdam, The Netherlands; 6 Department of Psychiatry and Behavioural Neurosciences, McMaster University, Hamilton, Ontario, Canada; 7 Centre for Evidence-Based Orthopaedics, Department of Orthopaedic Surgery, Zealand University Hospital, Køge, Denmark; 8 Department of Clinical Medicine, University of Copenhagen, Copenhagen, Denmark; 9 Biostatistics and Scientific Computing Department, King Faisal Specialist Hospital and Research Centre, Riyadh, Saudi Arabia; 10 Department of Epidemiology and Biostatistics, Schulich School of Medicine and Dentistry, University of Western Ontario, London, Ontario, Canada

**Keywords:** STATISTICS & RESEARCH METHODS, Methods, Patient Reported Outcome Measures, Psychometrics, Systematic Review

## Abstract

**Introduction:**

The Guidelines for Reporting Reliability and Agreement Studies (GRRAS) were developed to improve the completeness and transparency of reporting of reliability and agreement of health measurement instruments. However, since their publication in 2011 methodological standards, both for reporting guideline development and reliability, agreement and measurement error studies have advanced, highlighting the need for aligning and updating. In addition, related initiatives like COnsensus-based Standards for the selection of health Measurement INstruments (COSMIN) have emerged, offering opportunities to harmonise terminology and promote consistency across diverse types of measurement instruments. This method review aims to (1) systematically identify and synthesise commentaries on and evaluations of the original GRRAS and (2) map recent methodological developments in the planning, conduct and interpretation of reliability, agreement and measurement error studies in health science, psychology and education that should be reflected in the reporting, to inform the development of the GRRAS-COSMIN reporting guidelines.

**Methods and analysis:**

Two complementary search strategies will be employed. First, forward direct citation tracking of the original GRRAS publications will be conducted in Web of Science. We will include sources providing critique, commentaries or suggestions related to the GRRAS. We will exclude publications that used GRRAS just for structuring their report, withdrawn or retracted articles, textbooks, peer reviews, supplements and conference materials without full-text publications. Included articles will be analysed using descriptive content analysis. Second, we will perform a systematic search in MEDLINE, PsycInfo, Embase, ERIC and CINAHL supplemented by key references identified by or known to the author team. We will include studies, reviews, commentaries, editorials, methodological papers, tutorials and guidance documents that discuss or critically reflect on methodological aspects of the measurement properties reliability and/or agreement/measurement error published between 1 January 2015 and 30 June 2026 in health sciences, psychology or education. Eligibility will be assessed by two independent reviewers and included sources will be analysed using ‘codebook’ thematic analysis. Findings will be presented narratively supplemented by visuals and tables if appropriate.

**Ethics and dissemination:**

This study involves publicly available data. No ethical approval is needed. Results will be disseminated through an open-access journal publication following the Preferred Reporting Items for Systematic Reviews and Meta-Analyses-Scoping Review Extension and conference presentations.

**STRENGTHS AND LIMITATIONS OF THIS STUDY**

- This methods review protocol adheres to the methodological guidance provided by the Joanna Briggs Institute for conducting scoping reviews.
- Two complementary search strategies, each aligned with a specific review aim, are used, followed by tailored data synthesis for each.
- No language restrictions will be applied.
- A comprehensive search strategy is employed across multiple databases to ensure thorough coverage of the literature; however, the search strings are specific and focused, which may improve precision but could limit sensitivity, especially for methodological literature that is inconsistently indexed or published outside health, psychology and education databases.
- Grey literature will not be searched systematically; however, database searches will be supplemented by transparently documented additional sources.

## Introduction

In clinical, psychological or educational practice and research, obtained data (eg, blood pressure, values of standardised pain scales, personality tests, evaluations of examinations) or diagnoses (eg, risk of falling, depression, melanoma yes/no) must be reliable. Reliability and agreement/measurement error are fundamental properties of health measurement instruments, including patient-reported outcome measures (PROMs), performance-based instruments, diagnostic test results, clinician-reported outcomes or laboratory study results. While reliability and agreement are often used interchangeably, they reflect conceptually distinct constructs. Definitions vary slightly (see table 1), and for the purposes of this review, we adopt the following working definitions: *Reliability* refers to the ability of a measurement instrument to distinguish between individuals or objects.^1 2^ It is operationalised as the ratio of variability between subjects (eg, participants) to the total variability of all measurements in the sample. *Agreement*, also referred to as measurement error, is defined as the degree to which scores or ratings of a test or instrument are identical.^1 2^

**Table 1:** Selected examples of definitions of reliability and agreement as reported in the literature

	Reliability	Agreement/measurement error/error of measurement
COnsensus-based Standards for the selection of health Measurement INstruments (COSMIN)^54^	“The proportion of the total variance in the measurements which is due to the true difference between patients” (p. 1).^54^	“The systematic and random error of a patient’s score that is not attributed to the true changes in the construct to be measured” (p.1).^54^
Guidelines for Reporting Reliability and Agreement Studies (GRRAS)^1^	“[…] the ratio of variability between subjects (eg, patients) or objects (eg, computed tomography scans) to the total variability of all measurements in the sample” (p. 1).^1^ “[…] the ability of a measurement to differentiate between subjects or objects” (p.1).^1^	“[…] the degree to which scores or ratings are identical” (p.1).^1^
Standards for Educational and Psychological Testing^55^	“The degree to which test scores for a group of test takers are consistent over repeated applications of a measurement procedure and hence are inferred to be dependable and consistent for an individual test taker; the degree to which scores are free of random errors of measurement for a given group” (p.222).^55^	“The difference between an observed score and the corresponding true score” (p. 2019).^55^

Since reliability and agreement/measurement error are essential for judging the quality of measurement instruments, and by extension directly impact the quality of research and practice, their transparent reporting is crucial. Only what is transparently reported can be critically assessed, interpreted and built on. To improve the quality, transparency and consistency of reporting reliability, agreement and measurement error studies, a multidisciplinary working group developed the Guidelines for Reporting Reliability and Agreement Studies (GRRAS), published in 2011.^1 3^ Reporting guidelines—defined as ‘a checklist, flow diagram, or structured text to guide authors in reporting a specific type of research, developed using explicit methodology’^4^—are recognised as valuable for enhancing the transparency and completeness of study reporting across various research designs. By articulating key elements to be addressed, such guidelines enhance transparency, support interpretability and facilitate replicability and synthesis. Since its publication, GRRAS has been cited extensively and remains a frequently used reference for reporting reliability and agreement studies.

Nevertheless, several developments and ongoing challenges highlight the need to revisit and update the existing GRRAS. Central to these are enduring conceptual ambiguities, for example, the distinction between reliability and agreement/measurement error^5 6^ and the appropriate use of internal consistency measures.^7^ These uncertainties have contributed to methodological debates concerning the selection and interpretation of statistical coefficients^7 8^ and errors in the application and reporting of method and results. This, in turn, hampers efforts to synthesise evidence and evaluate measurement quality in systematic reviews.^1 9^ At the same time, important methodological discussions have advanced the assessment and interpretation of reliability, agreement and measurement error in health, psychological and educational research. These include discussions on agreement coefficients,^8 10 11^ recommendations for sample size determination for intraclass correlation coefficients, κ statistics and Bland-Altman approaches,^12–15^ guidance on the design, analysis and interpretation of reliability and measurement error studies.^2^ Commentaries on GRRAS have further emphasised the need for revision, offering constructive feedback and concrete suggestions for improvement.^16 17^

In parallel, related initiatives have emerged, offering opportunities for conceptual and terminological alignment. Notably, the COnsensus-based Standards for the selection of health Measurement INstruments (COSMIN) developed several guidelines for conducting and reporting studies on measurement properties of PROMs^18^ and for conducting and reporting systematic reviews on outcome measurement instruments,^19 20^ including a tool to assess the risk of bias in studies of reliability and agreement/measurement error.^21^ While COSMIN initially focused risk of bias assessments of existing studies on any measurement property, and GRRAS targets specifically on reporting of studies on reliability and agreement, both frameworks address shared constructs. Harmonising these efforts holds potential to improve clarity, promote consistency in terminology and enhance the usability and uptake of reporting standards. Similarly, the Quality Appraisal tool for studies of Diagnostic Reliability (QAREL), developed by Lucas *et al*,^22^ provides a structured framework for evaluating methodological quality in diagnostic reliability studies and is broadly applicable to reliability and agreement research.

In the related but different field of diagnostic accuracy, the Standards for Reporting of Diagnostic Accuracy Studies (STARD) statement^23 24^ offers a comprehensive set of recommendations to ensure transparent and complete reporting, while the Quality Assessment of Diagnostic Accuracy Studies (QUADAS) tool^25 26^ provides a structured approach for evaluating risk of bias of diagnostic accuracy studies included in systematic reviews.

Reporting and risk of bias assessment are inherently interdependent: only what is reported can be critically appraised. Therefore, reporting guidelines must explicitly include the elements targeted by risk of bias tools to ensure meaningful and comprehensive evaluation. In response to these developments and ongoing challenges, our multidisciplinary research group set out to develop the GRRAS-COSMIN guidelines, an updated and extended version of the original GRRAS.

### Objective

The objective of this methods review is to systematically identify and summarise published comments, evaluations and methodological discussions relevant to the development of the new GRRAS-COSMIN reporting guidelines for studies on reliability, agreement and measurement error. While reporting guidelines do not prescribe how studies should be designed, conducted or analysed, they should reflect contemporary methodological standards by ensuring that information relevant to assessing these aspects is reported transparently. Consequently, methodological developments may have implications not only for research practice itself, but also for what information should be reported to enable readers to judge whether current methodological recommendations have been followed. This process aligns with the methodological standards for reporting guideline development and revisions established by the EQUATOR Network.^27^

Specifically, this review will examine literature published in the last decade that either explicitly critiques or evaluates the existing GRRAS, or that provides methodological recommendations, advancements or reflections to inform best practice reporting of reliability, agreement and measurement error studies in health sciences, psychology and education. This includes issues related to the planning, conduct, analysis and interpretation of such studies.

Specific review questions are:

1. What comments and/or evaluations have been published about GRRAS?
2. What major methodological discussions, recommendations and/or advancements have been made in the last decade concerning the design, conduct, analysis, and reporting of reliability and agreement/measurement error studies?

## Methods

This protocol of a methods review is informed by the best practice guidelines for the development of scoping review protocols developed by the Joanna Briggs Institute^28^ as well as relevant guidance for conducting methods reviews.^29–32^ Results reporting will follow an adapted version of the Preferred Reporting Items for Systematic Reviews and Meta-Analysis extension for Scoping Reviews.^33^ To address the two review aims, we will implement two complementary search strategies.

### Eligibility criteria

To address review question 1, we will include peer-reviewed articles, commentaries or other scholarly publications that cite one or both of the original GRRAS publications^1 3^ and provide critique, commentaries or suggestions related to the GRRAS guideline. We will exclude articles that cite GRRAS solely to structure their report; withdrawn or retracted articles; textbooks; peer-reviews; supplements; and conference abstracts, posters or slide presentations without accompanying full-text publications. No language restrictions will be applied. Full texts published in languages other than English or German will be translated using DeepL Translator Pro. According to DeepL’s Privacy Policy, submitted texts are only processed temporarily to generate the translation, deleted after completion of the service and not used to improve DeepL’s services.^34 35^ Data extraction will be performed based on the translated text. In addition, where substantial contributions to the findings are derived from non-English or non-German sources, a back-translation procedure will be used for key excerpts to identify potential discrepancies in meaning.

Inclusion and exclusion criteria to address review question 2 following the Population, Concept and Context scheme are displayed in table 2.

**Table 2:** Inclusion and exclusion criteria for review question 2; structured following recommendations by Clarke *et al*^29^

	Inclusion criteria	Exclusion criteria
Types of documents	Studies, reviews, commentaries, editorials, methodological papers, dissertations and guidance documents that explicitly discuss, evaluate, reflect on or propose methodological aspects of the measurement properties reliability and/or agreement and/or measurement error. Relevant textbook chapters. Published between 1 January 2015 and 30 June 2026.	Empirical studies investigating measurement properties without critical methodological reflection. Reviews summarising measurement properties without methodological discussion. Conference abstracts, posters, slide presentations without full text. Errata, retracted or withdrawn articles.
Types of information	Narrative discussions, recommendations and proposals related to study design, planning, conduct, analysis, reporting and interpretation of reliability/agreement studies. Text on measurement error (absolute and relative), reliability/agreement statistics (eg, ICC, kappa, generalisability studies) and risk of bias appraisal.	Purely empirical numeric results on measurement properties without methodological reflection. Data on internal consistency/homogeneity. Data on reliability of automated systems.
Types of methods	Methods and approaches for designing, conducting, analysing and reporting reliability, agreement, and measurement error studies. Methods for appraising risk of bias in such studies.	Methods addressing automated systems or engineering applications only.
Types of disciplines	Health research, psychology and education.	Engineering; economics.

ICC, intraclass correlation coefficient.

### Information sources

To address review question 1, we will search Web of Science (WoS), which offers a broad and well-curated citation index across disciplines.

To address review question 2, we will consecutively search the databases Embase and MEDLINE via OVID and PsycInfo, ERIC and CINAHL via EBSCOhost. These databases were selected based on two considerations: first, a search in the Information Matrix for the Analysis of Journals (MIAR; https://miar.ub.edu) to identify databases indexing the journals of key references; and second, their relevance for covering the fields we aim to include in this review. In addition, we will search the Charité Library (via Ex Libris Primo) for relevant books and book chapters.

### Search strategy

To address review question 1, we will conduct direct forward citation tracking of the two original GRRAS guideline publications.^1 3^ To this end, we will search WoS for all articles citing the original publications. A pilot search on 1 July 2025 resulted in 1557 citations for reference^1^ and 441 citations for reference^3^ respectively.

To address review question 2, the search string shown in table 3 will be used with publication year restricted between 1 January 2015 to 30 June 2026. A detailed account of the development of the search string is documented in the supplement (online supplemental Table S1-S3). The search string was developed in MEDLINE via Ovid and will be translated to the other databases. The review will include supplementary sources identified by the author team. These sources are intended to supplement the systematic search by providing conceptual context, identifying seminal methodological contributions and reducing the risk of overlooking influential work that may not be retrieved through database searching alone. This may include articles about methodological discussions^8 36^ and relevant textbook chapters judged as important by the author team. Because these sources are intended to supplement the systematic database search, they are not necessarily subject to the same publication date restrictions. Additional sources will be included only if they are directly relevant to the review questions, and the source’s identification and reason for inclusion will be documented. A list of additional sources is presented in the supplement. This list is not exhaustive, and additional sources will be added during the search. Lastly, reference lists of all included articles will be screened for additional relevant sources using the SpiderCite tool from the Systematic Review (SR) Accelerator.^37^

10.1136/bmjopen-2025-109876.supp1 Supplementary data

**Table 3:** Search string to address review question 2

Date	Database	Search string	Hits
2026/0/02	MEDLINE via Ovid	Ovid MEDLINE(R) ALL <1946 to June 01, 2026>	2301
		1 reliability.kf,ti. 62933	
		2 agreement.kf,ti. 15474	
		3 measurement error.kf,ti. 2208	
		4 reproducibility of measurements.kf,ti. 104	
		5 interrater.kf,ti. 1751	
		6 intrarater.kf,ti. 407	
		7 intraobserver.kf,ti. 982	
		8 5 or 6 or 7 2900	
		9 Cohen's.kf,ti. 297	
		10 kappa.kf,ti. 20011	
		11 ICC.kf,ti. 1126	
		12 Gwet's.kf,ti. 18	
		13 guidance.kf,ti. 31327	
		14 guideline.kf,ti. 32524	
		15 tutorial.kf,ti. 2611	
		16 checklist.kf,ti. 9827	
		17 exp "Statistics as Topic"/ 3347331	
		18 exp "Reproducibility of Results"/ 514177	
		19 Research Design/ 135596	
		20 Publishing/ or "Access to Information"/ 39696	
		21 1 and 2 1109	
		22 3 or 4 or 8 or 9 or 10 or 11 or 12 26450	
		23 21 or 22 27336	
		24 13 or 14 or 15 or 16 75846	
		25 17 or 18 or 19 or 20 3776731	
		26 24 or 25 3843007	
		27 23 and 26 4982	
		28 limit 22 to yr="2015 - 2026" 11698	
		29 27 and 28 2301	

### Data management and selection process

The retrieved records will be exported from the respective databases in XML format. For deduplication and screening and dispute we will use the tools provided by the SR Accelerator,^37 38^ a web-based platform designed to support systematic review workflows, per review question. First, duplicate entries will be identified and removed prior to screening. Two reviewers (MMP and MM) will then independently assess titles and abstracts. Full texts of potentially relevant studies will be retrieved and independently assessed against the eligibility criteria. If no full text can be extracted using standard methods (eg, journal websites), we will (1) contact the library or (2) contact the corresponding author. Corresponding authors will be contacted twice with a follow-up after 2 weeks of no reply following the first contact. Reasons for exclusion during full text screening will be documented. Any disagreements will be resolved through discussions, with a third reviewer (JK) consulted in cases where consensus cannot be reached.

During full-text screening, we will also consider whether sources are published in journals or by publishers that raise concerns regarding editorial and publishing practices. For this purpose, we will use the Think. Check. Submit. checklist as guidance.^39^ Sources for which substantial concerns arise will be discussed within the review team before a final inclusion decision is made. Reasons for exclusion will be documented.

### Data collection process and data items

The first author (MMP) will extract the following descriptive information from all included full text: title, author, publication year, country of the corresponding author, publication type, language and field or discipline, and research question(s) or topic as stated by the authors (if available). The field or discipline of each source will be classified using the Organisation for Economic Co-operation and Development (OECD) Fields of Science categories.^40^ For sources indexed in WoS, WoS subject categories will be extracted and mapped to OECD categories using the InCites OECD Category Schema. For sources not indexed in WoS, such as textbooks, guidance documents or other supplementary sources, classification will be based on the source content, journal or book scope and, where informative, author departments or affiliations. Multiple fields may be assigned where appropriate, and unclear cases will be discussed within the review team. Contextual information will be generated during data synthesis described below.

### Risk of bias in individual studies

A critical appraisal of the evidence quality will not be conducted, since the included publication types are expected to be mostly opinion pieces which do not lend themselves to systematic quality assessment geared towards empirical studies.^28^

### Data synthesis

All included full-text publications will be uploaded to MAXQDA V.24^41^ and coded by the first author (MMP). Different qualitative analytical approaches will be employed to address each research question.

To answer review question 1, we will employ qualitative content analysis using a directed approach.^42^ The analysis is planned as deductive and descriptive, focusing on manifest content (ie, what the text says instead of underlying meaning of the text).^43^ Unit of analysis will be the individual sources. This approach is well-suited to systematically categorise and summarise clearly defined information, and thus is appropriate given our primary focus on descriptive and structured content, specifically how the GRRAS guidelines have been discussed or critiqued in the literature. Analysis will occur at the semantic level, emphasising explicit statements, which will be organised into descriptive categories and summarised narratively. Prespecified codes include the type of measurement property addressed, definitions of reliability and agreement (if provided), the specific GRRAS items discussed, recommendations for revision such as proposed new items or clarifications and commentary on the strengths and weaknesses of the original GRRAS.

To answer review question 2, we will use hybrid ‘codebook’ thematic analysis (TA) combining both deductive and inductive coding. We will use ‘codebook’ TA, defined by Braun and Clarke as a cluster of methods that use structured coding frameworks (eg, codebooks), where themes are typically identified early but may be refined through further analysis.^44^ This approach allows to begin with a set of a priori categories, while remaining open to inductively derived insights and aligns with our aim to describe the *main* developments, recurrent themes and topics in the field. In some cases, extracting relevant content will be relatively straightforward, for example, documenting key concepts from included tutorials that illustrate standard approaches. In other cases, the process will be more interpretative, for example, when grouping related methodological discussions into overarching categories and considering their relevance for reporting guidance. We will not apply an a priori threshold for what constitutes a ‘major’ methodological development. Instead, the relevance and prominence of identified methodological discussions will be described transparently, considering the content of the recommendation, the type of source, whether similar issues are discussed across multiple sources and the potential implications for reporting reliability, and agreement/measurement error studies. To enhance trustworthiness, we will use a structured codebook, document coding decisions, discuss uncertainties and changes to the codebook within the multidisciplinary author team and provide examples linking synthesised themes to the underlying source material.

The initial codebook will be structured around the following deductive codes, derived from our research objectives: measurement property addressed (eg, reliability, agreement, measurement error); methodological issues discussed (eg, study design recommendations, statistical methods, handling of measurement error (absolute/relative), risk of bias in reliability/agreement studies, reporting quality); whether existing frameworks are referenced (eg, COSMIN, QAREL, GRRAS, STARD); and definitions (eg, agreement, reliability). As coding progresses, inductive codes will be developed to capture ideas or concepts not anticipated in the initial framework. During synthesis, we will document whether themes are supported by systematically identified sources or arise mainly from supplementary sources.

Results will be synthesised descriptively. Study characteristics will be presented in tabular and graphical formats and summarised narratively in the text. For review question 1, we will map which specific GRRAS items have been commented on, identify proposals for their revision and clarifications. For review question 2, we will narratively summarise the key methodological developments identified through our hybrid ‘codebook’ TA, focusing on aspects related to the design, conduct, reporting and interpretation of reliability and agreement studies. These findings may be organised into overarching categories such as conceptual discussions, practical recommendations and methodological advancements. Furthermore, we aim to identify generated themes not currently addressed by GRRAS that may inform future development of the GRRAS-COSMIN guideline.

## Ethics and dissemination

This study will involve publicly available data. No ethical approval is needed. Results will be disseminated through an open-access journal publication and national and international conference presentations. Data curation and the analysis will be documented and openly accessible from the Open Science Framework.

### Patient and public involvement

Patients and/or the public were not involved in this research’s design, conduct, reporting or dissemination plans.

## Discussion

Reliability and agreement/measurement error are fundamental measurement properties in healthcare, psychology and education.^6 21 45–49^ Transparent, accurate and complete reporting of studies evaluating these properties is essential for the appropriate interpretation and application of measurement instruments. This methods review constitutes the first step in a structured revision of the GRRAS, informed by recent conceptual and methodological developments and conducted in accordance with established standards for reporting guideline development, as outlined by the EQUATOR Network.^4^ The results will inform the development of an initial draft of the GRRAS-COSMIN guideline for reporting studies on reliability and agreement/measurement error, which will subsequently be refined through a modified Delphi consensus process involving key stakeholders and experts.

Well-developed reporting guidelines serve as key tools for enhancing both the reporting and design of research. They provide structured guidance on the minimum essential information to be included when reporting specific types of studies, thereby supporting completeness, clarity and transparency.^50^ Although their primary function is to assist authors during manuscript preparation,^51^ the influence of reporting guidelines goes beyond. When considered early, for instance during study protocol development, reporting guidelines can support improved study planning. When followed during reporting, these guidelines ensure that readers have sufficient information to assess the methodological quality of the research, in the case of GRRAS, the reliability and agreement/measurement error properties of a given measurement instrument.

Improved reporting also benefits systematic reviewers, developers of clinical practice guidelines and those conducting electronic literature searches.^52^ Complete and standardised reporting is crucial for the conduct of meta-analyses and systematic reviews. Incomplete or inconsistent reporting poses significant barriers to the synthesis of findings across studies.^50^ In the field of reliability, agreement and measurement studies this limits the ability to appraise the quality of measurement instruments and hampers the development of cumulative evidence bases. Complete and standardised reporting is thus critical not only for individual study interpretation and informed decision-making but also for the advancement of measurement science more broadly.

Importantly, reporting guidelines do not dictate how research should be conducted. Rather, by articulating what information must be transparently reported, they indirectly encourage better research practices and facilitate the assessment of adherence to contemporary methodological standards. For example, inadequate reporting in clinical trials may result in inflated confidence in the efficacy of an intervention. Similarly, insufficient detail in studies of reliability and agreement can lead to incorrect conclusions about measurement properties. In both cases, the downstream consequences include misguided clinical decision-making and reduced reproducibility. Over time, the widespread use of rigorously developed reporting guidelines, such as the GRRAS-COSMIN reporting guideline, has the potential to enhance research quality by fostering greater methodological awareness and transparency across disciplines.

### Limitations

A comprehensive search strategy with no language restrictions will be employed across multiple databases to ensure thorough coverage of the literature. Nonetheless, this review has several methodological limitations that should be considered when interpreting the findings. First, the search strings are specific and focused, which may improve precision but could limit sensitivity and risk missing relevant literature. Specifically, this review seeks to identify methodological developments relevant to reliability, agreement and measurement error in health, psychological or educational research and focuses on databases that primarily index health, psychological and educational literature. Consequently, methodological developments published exclusively in, for example, mathematics or statistics journals may not be comprehensively captured.

Second, because the review includes diverse publication types such as methodological papers, tutorials, editorials, commentaries, guidance documents and textbook material, and because the objective is to identify methodological developments rather than evaluate the validity of these studies, no formal critical appraisal of included sources will be undertaken. Consequently, the review will describe and synthesise methodological developments discussed in the literature but will not assess the relative merit or empirical support of competing methodological approaches.

Third, identifying and synthesising methodological developments requires a degree of interpretive judgement. This is inherent to qualitative synthesis.^53^ We will address this through study selection being performed independently by two reviewers, documentation of coding decisions, reflexive discussions within the multidisciplinary author team, and transparent reporting of how themes are supported by the included sources.

Finally, while the absence of language restrictions may reduce the risk of language bias, non-English and non-German sources will be translated using automated translation tools. Consequently, inaccuracies in translation or subtle differences in terminology and meaning may affect the interpretation of methodological concepts, definitions or recommendations. We will seek to minimise this risk by checking key passages against the original text where necessary.

## Supplementary Material

Reviewer comments

Author's manuscript
