# Supplementary material for: Developments in the field of reliability and agreement studies in health, psychological and educational measurement: a methods review protocol
Source: BMJ Open. 2026 Jul 22;16(7):e109876. doi: 10.1136/bmjopen-2025-109876 (PMC13410919; doi:10.1136/bmjopen-2025-109876)
Supplement: Supplementary data [file bmjopen-16-7-s001.pdf]

**Supplement of: Developments in the field of reliability and agreement studies in health, psychological, and educational measurement: a methods review protocol**

Pittelkow, M.-M., Mönch, M., Mokkink, L.B., Streiner, D. L., Brorson S., Shoukri M., Kottner J.

This supplement details the sample references and search string development corresponding to review question 2 of the protocol “Developments in the field of reliability and agreement studies in health, psychological, and educational measurement: a methods review protocol
”.

**Review question 2**: What major methodological discussions, recommendations, and/or advancements have been made in the last decade concerning the design, conduct, analysis, and reporting of reliability, and agreement/measurement error studies?

**Sample references**

This is a preliminary list of key references to be included in the scoping review in addition to the sources identified via the systematic search. Please note that this list is not exhaustive and sources may be added during the conduct of the review.

**Journal articles**

1. Borg, D. N., Bach, A. J. E., O’Brien, J. L., & Sainani, K. L. (2022). Calculating sample size for reliability studies. PM & R : The Journal of Injury, Function, and Rehabilitation, 14(8), 1018–1025. https://doi.org/10.1002/pmrj.12850
2. Cohen JF, Korevaar DA, Altman DG, Bruns DE, Gatsonis CA, Hooft L, Irwig L, Levine D, Reitsma JB, de Vet HC, Bossuyt PM. STARD 2015 guidelines for reporting diagnostic accuracy studies: explanation and elaboration. BMJ Open. 2016 Nov 14;6(11):e012799. https://doi.org/10.1136/bmjopen-2016-012799.
3. Koo, T. K., & Li, M. Y. (2016). A Guideline of Selecting and Reporting Intraclass Correlation Coefficients for Reliability Research. Journal of Chiropractic Medicine, 15(2), 155–163. https://doi.org/10.1016/j.jcm.2016.02.012
4. Mokkink, L. B., Boers, M., van der Vleuten, C. P. M., Bouter, L. M., Alonso, J., Patrick, D. L., de Vet, H. C. W., & Terwee, C. B. (2020). COSMIN Risk of Bias tool to assess the quality of studies on reliability or measurement error of outcome measurement instruments: A Delphi study. BMC Medical Research Methodology, 20(1), 293. https://doi.org/10.1186/s12874-020-01179-5
5. Mokkink, L. B., de Vet, H., Diemeer, S., & Eekhout, I. (2023). Sample size recommendations for studies on reliability and measurement error: An online application based on simulation studies.

Health Services and Outcomes Research Methodology, 23(3), 241–265. https://doi.org/10.1007/s10742-022-00293-9

6. Mokkink, L. B., Eekhout, I., Boers, M., van der Vleuten, C. P. M., & de Vet, H. C. W. (2023). Studies on Reliability and Measurement Error of Measurements in Medicine – From Design to Statistics Explained for Medical Researchers. Patient Related Outcome Measures, 14, 193–212. https://doi.org/10.2147/PROM.S398886
7. Oster, N. V., Carney, P. A., Allison, K. H., Weaver, D. L., Reisch, L. M., Longton, G., Onega, T., Pepe, M., Geller, B. M., Nelson, H. D., Ross, T. R., Tosteson, A. N. A., & Elmore, J. G. (2013). Development of a diagnostic test set to assess agreement in breast pathology: Practical application of the Guidelines for Reporting Reliability and Agreement Studies (GRRAS). BMC Women's Health, 13(1), 3. https://doi.org/10.1186/1472-6874-13-3
8. Stratford, P., Spadoni, G. F., Kuspinar, A., & Macedo, L. (2025). Considerations when designing, analyzing, and reporting reliability studies. Brazilian Journal of Physical Therapy, 29(3), 101193. https://doi.org/10.1016/j.bjpt.2025.101193
9. Vach, W., & Gerke, O. (2023). Gwet's AC1 is not a substitute for Cohen's kappa – A comparison of basic properties. MethodsX, 10, 102212. https://doi.org/10.1016/j.mex.2023.102212
10. Vet, H. C. W. de, Mokkink, L. B., Terwee, C. B., Hoekstra, O. S., & Knol, D. L. (2013). Clinicians are right not to like Cohen's κ. BMJ, 346, f2125. https://doi.org/10.1136/bmj.f2125
11. Tran, D., Dolgun, A., & Demirhan, H. (2020). Weighted inter-rater agreement measures for ordinal outcomes. *Communications in Statistics - Simulation and Computation*, *49*(4), 989–1003. https://doi.org/10.1080/03610918.2018.1490428
12. ten Hove, D., Jorgensen, T. D., & van der Ark, L. A. (2024). Updated guidelines on selecting an intraclass correlation coefficient for interrater reliability, with applications to incomplete observational designs. Psychological Methods, 29(5), 967–979. https://doi.org/10.1037/met0000516

**Textbooks**

13. De Vet, H. C., Terwee, C. B., Mokkink, L. B., & Knol, D. L. (2011). *Measurement in medicine: a practical guide*. Cambridge university press.
14. American Educational Research Association, American Psychological Association, & National Council on Measurement in Education. (2014). Standards for educational and psychological testing. American Educational Research Association.

15. Streiner, D. L., Norman, G. R., & Cairney, J. (2024). *Health Measurement Scales: A practical guide to their development and use* (Sixth Edition, Sixth Edition). Oxford University Press.

**Manual**

16. Mokkink, L. B., E. Elsman, C.B. Terwee. COSMIN guideline for systematic reviews of Patient-Reported Outcome Measures version 2.0. Qual Life Res (2024). https://doi.org/10.1007/s11136-024-03761-6.

**Search String Development**

The search string was developed with both sensitivity (maximizing the identification of relevant articles) and feasibility (retrieving a manageable number of records for screening by two reviewers) in mind. Finding this balance can be especially challenging for reviews of methods topics [1]. We started by compiling a reference sample based on PubMed IDs of key journal articles (i.e., 28137831, 27330520, 37448975, 23379630, 27464308, 40022968, 37234937, 23585065, 33267819, 35596122). These articles serve as examples of the types of methodological literature we aim to identify and are not necessarily expected to be fully captured by the final search string. For example, we decided a priori to limit the search to publications from the last decade (1.1.2015 to 30.6.2025), since our aim is to cover recent methodological discussions and advancements. As a result, some of the articles included in the reference sample may not appear in the search output due to their earlier publication date.

In a first step, we identified relevant search terms and Medical Subject Headings (MeSH) terms. To this end, we (1) used the Systematic Review Accelerator (SR-Accelerator) [2] to extract and review frequently used words in the key references (see table S1) and (2) reviewed the assigned MeSH terms for the reference sample using the Yale Mesh Analyzer (1, see table S2). We additionally consulted the term finder in Ovid for MeSH terms related to reliability, agreement, or measurement error as well as the Thesaurus for Health Research Methods from the Library of Guidance for Health Scientists [4].

*Table S1. Word frequencies in title, abstract, and keywords of key references.*

| word | unique | title | abstract | keywords | points |
|---|---|---|---|---|---|
| reliability | 6 | 5 | 18 | 0 | 23 |
| agreement | 4 | 3 | 18 | 0 | 21 |
| measurement | 3 | 4 | 12 | 0 | 16 |
| study | 3 | 1 | 14 | 0 | 15 |
| icc | 2 | 0 | 14 | 0 | 14 |
| studies | 5 | 6 | 7 | 0 | 13 |
| between | 5 | 1 | 10 | 0 | 11 |
| cohen’s | 2 | 1 | 9 | 0 | 10 |
| reporting | 4 | 4 | 6 | 0 | 10 |
| methods | 3 | 1 | 8 | 0 | 9 |
| kappa | 1 | 1 | 8 | 0 | 9 |
| ac1 | 1 | 1 | 8 | 0 | 9 |
| design | 4 | 0 | 9 | 0 | 9 |
| gwet’s ac1 | 1 | 0 | 8 | 0 | 8 |
| gwet’s | 1 | 0 | 8 | 0 | 8 |
| error | 2 | 2 | 6 | 0 | 8 |
| measure | 1 | 0 | 7 | 0 | 7 |
| outcome measurement | 1 | 1 | 6 | 0 | 7 |
| outcome | 1 | 1 | 6 | 0 | 7 |
| cohen’s kappa | 1 | 0 | 7 | 0 | 7 |
| measurements | 2 | 0 | 7 | 0 | 7 |
| form | 1 | 0 | 6 | 0 | 6 |
| values | 4 | 0 | 6 | 0 | 6 |
| test | 4 | 2 | 4 | 0 | 6 |
| repeated | 2 | 0 | 6 | 0 | 6 |
| measurement error | 2 | 2 | 4 | 0 | 6 |
| size | 2 | 1 | 5 | 0 | 6 |
| **word** | **unique** | **title** | **abstract** | **keywords** | **points** |
| sample | 2 | 1 | 5 | 0 | 6 |
| when | 3 | 1 | 5 | 0 | 6 |
| κ | 1 | 1 | 4 | 0 | 5 |
| article | 1 | 0 | 5 | 0 | 5 |
| instrument | 1 | 0 | 5 | 0 | 5 |
| assess | 2 | 2 | 3 | 0 | 5 |
| rate | 1 | 0 | 5 | 0 | 5 |

| | | | | | |
|---|---|---|---|---|---|
| researchers | 3 | 0 | 5 | 0 | 5 |
| ratings | 3 | 0 | 5 | 0 | 5 |
| different | 4 | 0 | 5 | 0 | 5 |
| scores | 2 | 0 | 5 | 0 | 5 |
| correlation | 2 | 1 | 4 | 0 | 5 |
| e | 3 | 0 | 5 | 0 | 5 |
| based | 3 | 1 | 4 | 0 | 5 |
| raters | 4 | 0 | 5 | 0 | 5 |
| information | 4 | 1 | 4 | 0 | 5 |
| results | 3 | 0 | 5 | 0 | 5 |
| sample size | 2 | 1 | 4 | 0 | 5 |
| purpose | 1 | 0 | 5 | 0 | 5 |
| quantity | 1 | 0 | 4 | 0 | 4 |
| clinicians | 1 | 1 | 3 | 0 | 4 |
| diagnostic | 2 | 2 | 2 | 0 | 4 |
| research | 3 | 1 | 3 | 0 | 4 |
| reached | 1 | 0 | 4 | 0 | 4 |
| consensus | 1 | 0 | 4 | 0 | 4 |
| variation | 2 | 0 | 4 | 0 | 4 |
| how | 1 | 0 | 4 | 0 | 4 |

| word | unique | title | abstract | keywords | points |
|---|---|---|---|---|---|
| measurement instruments | 1 | 1 | 3 | 0 | 4 |
| instruments | 1 | 1 | 3 | 0 | 4 |
| quality | 1 | 1 | 3 | 0 | 4 |
| agreement rate | 1 | 0 | 4 | 0 | 4 |
| positive | 2 | 0 | 4 | 0 | 4 |
| properties | 2 | 1 | 3 | 0 | 4 |
| developed | 3 | 0 | 4 | 0 | 4 |
| k | 1 | 0 | 4 | 0 | 4 |
| repeated measurements | 1 | 0 | 4 | 0 | 4 |
| patients | 2 | 0 | 4 | 0 | 4 |
| application | 2 | 2 | 2 | 0 | 4 |
| calculation | 3 | 0 | 4 | 0 | 4 |
| discussion | 3 | 0 | 4 | 0 | 4 |
| provide | 3 | 0 | 4 | 0 | 4 |
| study purpose | 1 | 0 | 4 | 0 | 4 |
| however | 4 | 0 | 4 | 0 | 4 |
| observations | 1 | 1 | 2 | 0 | 3 |
| same | 2 | 0 | 3 | 0 | 3 |
| cohen's κ | 1 | 1 | 2 | 0 | 3 |
| readers | 1 | 0 | 3 | 0 | 3 |
| appropriate | 2 | 0 | 3 | 0 | 3 |
| icc form | 1 | 0 | 3 | 0 | 3 |
| guidelines | 2 | 2 | 1 | 0 | 3 |
| breast | 1 | 1 | 2 | 0 | 3 |
| diagnostic test | 2 | 2 | 1 | 0 | 3 |

| word | unique | title | abstract | keywords | points |
|---|---|---|---|---|---|
| reported | 3 | 0 | 3 | 0 | 3 |
| standards | 1 | 0 | 3 | 0 | 3 |
| measurement instrument | 1 | 0 | 3 | 0 | 3 |
| tool | 2 | 1 | 2 | 0 | 3 |
| both | 2 | 0 | 3 | 0 | 3 |
| paper | 3 | 0 | 3 | 0 | 3 |
| using | 3 | 0 | 3 | 0 | 3 |
| agreement between | 2 | 1 | 2 | 0 | 3 |
| basic | 2 | 1 | 2 | 0 | 3 |
| most | 2 | 0 | 3 | 0 | 3 |
| mse | 1 | 0 | 3 | 0 | 3 |
| parameters | 2 | 0 | 3 | 0 | 3 |
| about | 3 | 0 | 3 | 0 | 3 |
| intraclass correlation | 2 | 1 | 2 | 0 | 3 |
| intraclass | 2 | 1 | 2 | 0 | 3 |
| number | 2 | 0 | 3 | 0 | 3 |
| online application | 1 | 1 | 2 | 0 | 3 |
| online | 1 | 1 | 2 | 0 | 3 |
| whether | 2 | 0 | 3 | 0 | 3 |
| reliability study | 3 | 0 | 3 | 0 | 3 |
| reliability studies | 2 | 1 | 2 | 0 | 3 |
| reporting reliability | 2 | 2 | 1 | 0 | 3 |

Table S2. Results from the Yale MeSH Analyzer

| **PMID** | 28137831 | 27330520 | 37448975 | 23379630 | 27464308 | 40022968 | 37234937 | 23585065 | 33267819 |
|---|---|---|---|---|---|---|---|---|---|
| **MeSH Headings** | Advisory Committees* | | | | | | | | |
| | Bias<br>Biomedical Research | | | Breast / pathology<br>Breast Diseases / pathology*<br>Breast Neoplasms / classification*<br>Breast Neoplasms / diagnosis<br>Breast Neoplasms / pathology* | | | | | Bias |
| | Checklist* | | | | | | | | Consensus |
| | Diagnostic Techniques and Procedures / standards* | | | Diagnosis, Differential | Diagnostic Tests, Routine / standards* | | | | Delphi Technique* |
| | | | | Female | | | | | |
| | | | | | Guidelines as Topic | | | | |
| | Humans | | | Humans | Humans | Humans | | Humans | Humans |
| | | | | Observer Variation | | | | Observer Variation* | |

(continued)

Table S2 continued. Results from the Yale MeSH Analyzer

| **PMID** | 28137831 | 27330520 | 37448975 | 23379630 | 27464308 | 40022968 | 37234937 | 23585065 | 33267819 |
|---|---|---|---|---|---|---|---|---|---|
| | Publishing | | | Predictive Value of Tests | Psychology / standards<br>Psychometrics / standards*<br>Publishing / standards* | | | Probability* | |
| | | | | Quality Assurance, Health Care / standards* | | | | | |
| | Research Design | | | Reproducibility of Results<br>Research Design / standards | | Reproducibility of Results<br>Research Design* | | Reproducibility of Results*<br>Research Design* | Reproducibility of Results |
| | | | | Sensitivity and Specificity | Societies, Scientific | Sample Size | | | |
| | Terminology as Topic | | | | | | | | |
| **Author Assigned Keywords** | Diagnostic accuracy<br>Medical publishing<br>Peer review<br>Reporting quality<br>Research waste<br>Sensitivity and specificity | Reliability and validity<br>Research<br>Statistics | classical test theory<br>generalizability theory<br>intraclass correlation coefficient<br>measurement error<br>reliability<br>standard error of measurement | | | Measurement Methods<br>Rehabilitation<br>Reliability<br>Research design<br>Statistics | Agreement<br>Gwet's AC1<br>Method comparison<br>Observer<br>Rater<br>Reliability<br>Repeatability<br>Reproducibility | | COSMIN<br>Delphi study<br>Measurement error<br>Outcome measurement instruments<br>Quality assessment<br>Reliability<br>Risk of Bias |

From this, we constructed a set of possible search and MeSH terms (see table S3).

**Table S3. Potential search and MeSH terms. MeSH terms are retrieved from Medline via Ovid**

| **Search concept** | **Potential search terms** | |
|---|---|---|
| **Target:** | Reliability | Method* |
| | reproducibility of measurements | Outcome |
| **Methodological studies, reviews, commentaries, editorials, and guidance documents that explicitly discuss, evaluate, reflect on, or propose methodological aspects of reliability, agreement, and/or measurement error.** | Agreement | Cohen's |
| | Bland Altmann analysis | Kappa |
| | Measurement | ICC |
| | Measurement error | AC1 |
| | Measure* | gwet's |
| | Stud* | Guidance |
| | Reporting | How to |
| | Reporting quality | Best practice |
| | MeSH: Research Design / standards | MeSH: Statistics as Topic |
| | MeSH: Reproducibility of Results | MeSH: Reproducibility of Results |
| | MeSH: Publishing / standards* | MeSH: Methods / Research Design |

We then iteratively piloted the search terms in Medline via OVID to develop a combination that was both sensitive and resulted in a feasible number of hits. To check the sensitivity of our search, we searched for the reference sample in the hits of the search string before adding the publication date restriction.

**References**


1 Hirt J, Ewald H, Briel M, *et al.* Searching a methods topic: practical challenges and implications for search design. *J Clin Epidemiol*. 2024;166:111201. doi: 10.1016/j.jclinepi.2023.10.017

2 Clark J, Glasziou P, Del Mar C, *et al.* A full systematic review was completed in 2 weeks using automation tools: a case study. *J Clin Epidemiol*. 2020;121:81–90. doi: 10.1016/j.jclinepi.2020.01.008

3 Yale MeSH Analyzer. https://mesh.med.yale.edu/ (accessed 23 July 2025)

4 Hirt J, Schönenberger CM, Ewald H, *et al.* Introducing the Library of Guidance for Health Scientists (LIGHTS): A Living Database for Methods Guidance. *JAMA Netw Open*. 2023;6:e2253198. doi: 10.1001/jamanetworkopen.2022.53198
