# Supplementary material for: Developments in the field of reliability and agreement studies in health, psychological and educational measurement: a methods review protocol
Source: BMJ Open. 2026 Jul 22;16(7):e109876. doi: 10.1136/bmjopen-2025-109876 (PMC13410919; doi:10.1136/bmjopen-2025-109876)
Supplement: Reviewer comments [file bmjopen-2025-109876.reviewer_comments.pdf]

PEER REVIEW HISTORY

BMJ Open publishes all reviews undertaken for accepted manuscripts. Reviewers are asked to complete a checklist review form and are provided with free text boxes to elaborate on their assessment. These free text comments are reproduced below.

ARTICLE DETAILS

Title (Provisional)

Developments in the field of reliability and agreement studies in health, psychological, and educational measurement: a methods review protocol

Authors

Pittelkow, M.-M.; Mönch, M; Mokkink, L.B.; Streiner, D. L.; Brorson, S.; Shoukri, M.; Kottner, J.

VERSION 1 - REVIEW

**Reviewer**	**1**
**Name**	**Heng, Marilyn**
**Affiliation**	**Boston Medical Center - Brighton**
**Date**	**20-Dec-2025**
**COI**	**None**

The authors present a Methods review protocol for a scoping review of the literature regarding developments for reporting reliability and agreement studies. They focus on publications citing the original GRASS guidelines and methodological advancements concerning reliability and measurement agreement studies. This protocol manuscript is well written and provides clear explanation of how the authors intend to proceed with the review. I have no concerns or suggestions for the manuscript as I think it is clearly and completely presented.

**Reviewer**	**2**
**Name**	**Vanbelle, S**
**Affiliation**	**Maastricht University**
**Date**	**21-Apr-2026**
**COI**	**None**

The authors have undertaken a valuable initiative with this systematic literature review project to improve the GRRAS guidelines by integrating them with the COSMIN toolkit.

Clinimetrics represents an important field of research with implications across many scientific areas. I have some comments regarding the way that the review will be conducted, mainly asking for clarifications for the study to be reproducible.

# Comment 1

I was invited to review this paper in April 2026. Given that the protocol will be published after this date, it would be appropriate to extend the search timeframe to April 2026. At present, the proposed search deadline is nearly one year earlier, and the manuscript also reports inconsistent dates (30-06-2025 on page 8, line 60, and 31-07-2025 in the abstract). Updating the search to April 2026 would clarify the timeframe and should not require substantial additional effort.

# Comment 2

The search is limited to health sciences, education and psychology. Mathematics and statistics are important fields where methodological developments are made. Indeed, developing methods to plan, analyse and interpret reliability, agreement/measurement error studies is by definition a mathematical and statistical problem. I advise the authors to include these fields in their research strategy or to strongly motivate and discuss why these research fields will not be included.

#Comment 3

I see some simulation study cited in the paper, e.g.,

Mokkink LB, de Vet H, Diemeer S, *et al.* Sample size recommendations for studies on reliability and measurement error: an online application based on simulation studies. *Health Serv Outcomes Res Methodol*. 2023;23:241–65. doi: 10.1007/s10742-022-00293-9

while on Page 11, line 19, the following is stated: “reporting guidelines do not dictate how research should be conducted. Rather, by articulating what information must be transparently reported, they indirectly encourage better research practices.”

The difference between “what elements to report” and “how to determine the reported elements” should be clarified in the manuscript. The focus of the paper is not clear as it cites papers making e.g., sample size recommendations while in the discussion,

focusing on “what elements to report”. If the message is in fine “to report how sample size was determined”, then I believe that the above paper doesn’t add to the current guidelines, for example.

#Comment 4

It is somewhat surprising that no mathematical or statistical literature is cited in the discussion of sample sizes, as these are precisely the fields in which methodological developments would be expected.

#Comment 5

Related to Comment 4: It remains unclear how the quality of, for example, simulation studies will be evaluated and how the adequacy of mathematical developments will be assessed. Established guidance already exists for the design and reporting of simulation studies (e.g., the ADEMP guidelines), and such frameworks should be considered when evaluating literature.

It is therefore essential that the authors explicitly describe how the quality of the included literature will be evaluated and which objective assessment tools or criteria will be applied, particularly given that the authors will be evaluating their own work.

#Comment 6

The list of keywords appears rather limited. For instance, in MeSH the indexed terms include *reproducibility of results* and *observer variation* (including intraobserver and interobserver variation). The authors should therefore carefully reconsider and broaden their keyword strategy.

Different scientific fields often use different terminology for closely related concepts. For example, in psychology, variance partitioning coefficients derived from generalisability theory are commonly used to study reliability. In the health sciences, terms such as precision, accuracy, repeatability, and replicability are used (some of these words are included in Appendix 2).

Similarly, including variants of Cohen’s kappa, such as Fleiss’ kappa, Scott’s pi, and similar measures would likely improve coverage of the literature search. The terminology and naming of concepts vary substantially across disciplines and many variants of Cohen’s kappa and intraclass correlation coefficients were introduced over the years. The authors are invited to expand the keyword list accordingly to reduce the risk of missing relevant literature.

# Comment 7

P2, line 30: “Second, we will perform a systematic search in Medline, PsycINFO, Embase, Eric, and Cinahl supplemented by key references identified by or known to the author team.”

It is unclear how the authors will prevent bias in the selection of “key references identified by or known to the author team.” As currently phrased, this approach appears subjective and potentially prone to selection bias. The authors should therefore clarify the procedures.

# Comment 8

P2, line 34:” in the field of health measurements” and P11, line 39-40 “healthcare, psychology and education”. The authors should clarify how these areas relate to one another within the context of the review and provide a clear rationale for their inclusion (and limitation).

# Comment 9

“At the same time, important advances have been made in areas such as sample size determination, handling of measurement error, and the appraisal of risk of bias in studies of reliability and agreement [12–14].” Related to Comment 2, the literature cited here appears highly selective and does not include references from the mathematical or statistical literature, where many of the methodological developments in these areas originate. What is the rationale to cite these three studies in particular?

# Comment 10

P7, line 37: I am a bit confused by the sentence. I understand that peer-reviews, textbooks and dissertations are excluded. It is unclear to me why, while websites are included as eligible sources. This choice appears difficult to justify from a methodological perspective, as the former typically undergo formal scholarly review or editorial oversight, whereas websites may vary considerably in terms of reliability and scientific rigor. The authors should clarify the rationale for this decision and explain how the credibility and quality of website sources will be ensured. Further, the authors should give objective criteria to define a website as “recognized methodological website”. A last issue with website is how to monitor the date of creation of a webpage

with respect to the time window of the literature review. The authors should specify how they will proceed.

I think that recent dissertations could be interesting as sometimes they include chapters that are not yet published or are still under review.

# Comment 11

P7, line 40: It is unclear how texts written in languages other than German or English will be handled. The authors should clarify the procedures that will be used for translation and data extraction from such sources. In particular, it would be important to specify whether professional translation, native-speaking reviewers, or automated translation tools will be used, and how the accuracy and consistency of the translations will be ensured. In a context where wording is important (see Table 1), this is a important detail.

# Comment 12

P8, line 32-33: related to comments 2 and 9. What about statistics and mathematics?

# Comment 13

P9, line 23: "The scoping review will include additional key sources identified by the author team." It is unclear how the authors will ensure that the selection of such additional key sources is conducted in a transparent and unbiased manner (related to Comment 7). As formulated, this approach appears subjective and may introduce selection bias. The authors should therefore clarify the procedures and criteria that will guide the identification and inclusion of these sources.

# Comment 14

P9, lines 4-5: This includes articles about methodological discussions [10,33], resources from the COSMIN website and relevant textbooks. Do the materials hosted on the COSMIN website not correspond to formally published or peer-reviewed work? Are textbooks excluded or included? (see page 7, line 37).

# Comment 15

P10, line 18: “discipline as indicated by the affiliation of the corresponding author (e.g., health sciences, psychology, education).”

It is unclear why the discipline is determined specifically based on the affiliation of the corresponding author. This criterion appears arbitrary, as research papers often involve multidisciplinary teams, and the corresponding author’s affiliation may not accurately reflect the methodological orientation of the publication. The authors should justify why it is considered an appropriate proxy for classifying disciplinary background, or alternatively consider a more robust and transparent classification strategy.

#Comment 16

P10, line 56: “In other cases, the process will be more interpretative, such as determining what qualifies as a major methodological development, which will require informed judgment from the research team”.

This formulation appears highly subjective and raises concerns regarding consistency and reproducibility. The authors should explicitly define objective criteria for what will be considered a “major methodological development” and specify how these criteria will be applied in practice. In addition, it is important to describe the procedures that will be used to minimise bias in these judgments.

#Comment 17

How will the authors handle publications from predatory journals or other potentially non-credible sources? The manuscript does not specify any strategy for identifying or excluding such publications. Given the potential impact of low-quality or non-peer-reviewed documents (such as websites), a strategy should be explained.

#Comment 18.

Appendix:

11. De Vet, H. C., Terwee, C. B., Mokkink, L. B., & Knol, D. L. (2011). *Measurement in medicine: a practical guide*. Cambridge university press.

It is unclear why this reference has been included, given that it was published before the stated search period (01.01.2015–30.06.2025). The authors should clarify the rationale for its inclusion.

# Comment 19

I invite the authors to explicitly discuss potential methodological limitations, including possible sources of bias, limitations in the search strategy, as well as their potential impact on the robustness and interpretability of the findings.

VERSION 1 - AUTHOR RESPONSE

Reviewer 1:

The authors present a Methods review protocol for a scoping review of the literature regarding developments for reporting reliability and agreement studies. They focus on publications citing the original GRASS guidelines and methodological advancements concerning reliability and measurement agreement studies. This protocol manuscript is well written and provides clear explanation of how the authors intend to proceed with the review. I have no concerns or suggestions for the manuscript as I think it is clearly and completely presented.

Thank you for this very positive review.

Reviewer 2:

The authors have undertaken a valuable initiative with this systematic literature review project to improve the GRRAS guidelines by integrating them with the COSMIN toolkit. Clinimetrics represents an important field of research with implications across many scientific areas. I have some comments regarding the way that the review will be conducted, mainly asking for clarifications for the study to be reproducible.

<u># Comment 1:</u> I was invited to review this paper in April 2026. Given that the protocol will be published after this date, it would be appropriate to extend the search timeframe to April 2026. At present, the proposed search deadline is nearly one year earlier, and the manuscript also reports inconsistent dates (30-06-2025 on page 8, line 60, and 31-07-2025 in the abstract). Updating the search to April 2026 would clarify the timeframe and should not require substantial additional effort.

The search will be updated accordingly. We opted to change the timeframe to *01.01.2015 to 30.06.2026* to also account for the time of this revision. Changes are reflected:

- Abstract, line 53: "published between *01.01.2015 and 30.06.2026 in health sciences, psychology, or education"*
- Table 3: „Published between 01.01.2015 and *30.06.2026.*"
- Line 200f: "To address review question 2, the search string shown in Table 3 will be used with publication year restricted between 1.1.2015 to *30.6.2026*."

<u># Comment 2:</u> The search is limited to health sciences, education and psychology. Mathematics and statistics are important fields where methodological developments are made. Indeed, developing methods to plan, analyse and interpret reliability, agreement/measurement error studies is by definition a mathematical and statistical problem. I advise the authors to include these fields in their research strategy or to strongly motivate and discuss why these research fields will not be included.

We completely agree that these are relevant fields and disciplines. During the development and piloting of the search strategy, we found that relevant methodological publications relating to our targeted disciplines authored by statisticians and methodologists were frequently indexed in the databases searched and were retrieved by our search terms. In addition, several key methodological papers and guidance documents authored by statisticians and methodologists (e.g., de Vet et al., 2013; ten Hove et al., 2024; Tran et al., 2020; Vach & Gerke, 2023) are already included among the seed references and additional sources considered during the review process.

Nevertheless, we acknowledge that the primary focus of this review is on methodological developments relevant to the reporting of reliability and agreement studies in health, psychological, and educational research. Accordingly, our search strategy prioritises databases covering psychometrics, clinimetrics, and applied measurement research rather than the broader mathematical or statistical literature. We have now clarified this scope in the manuscript. We also acknowledge that methodological developments published exclusively in mathematics or statistics journals and not indexed in the selected databases may not be comprehensively captured and have added this to the limitations.

To this end, we have made the following changes:

- Line 1: "Developments in the field of reliability and agreement studies *in health, psychological, and educational measurement*: a methods review protocol"
- Line 37ff: "This methods review aims (1) to systematically identify and synthesize commentaries on and evaluations of the original GRRAS, and (2) to map recent methodological developments in the planning, conduct, and interpretation of reliability, agreement and measurement error studies *in health science, psychology and education* that should be reflected in the reporting, to inform the development of the GRRAS-COSMIN reporting guidelines."
- Line 69 ff.: A comprehensive search strategy is employed across multiple databases to ensure thorough coverage of the literature; however, the search strings are specific and focused, which may improve precision but could limit sensitivity, *especially for methodological literature that is inconsistently indexed or published outside health, psychology, and education databases*.
- Line 74ff.: Grey literature will not be searched systematically; *however database searches will be supplemented by transparently documented additional sources*.
- Line 155 ff.: "Specifically, this review will examine literature published in the last decade that either explicitly critiques or evaluates the existing GRRAS, or that provides methodological recommendations, advancements, or reflections to inform best practice reporting of reliability, agreement and measurement error studies *in health sciences, psychology, education*."
- Line 349: *"First, the search strings are specific and focused, which may improve precision but could limit sensitivity and risk missing relevant literature. Specifically, this review seeks to identify methodological developments relevant to reliability, agreement, and measurement error in health, psychological or educational research and focuses on databases that primarily index health, psychological, and educational literature. Consequently, methodological developments published exclusively in, for example, mathematics or statistics journals may not be comprehensively captured."*

#Comment 3: **I see some simulation study cited in the paper, e.g., Mokkink LB, de Vet H, Diemeer S, et al. Sample size recommendations for studies on reliability and measurement error: an online application based on simulation studies. Health Serv Outcomes Res Methodol. 2023;23:241–65. doi: 10.1007/s10742-022-00293-9 while on Page 11, line 19, the following is stated: "reporting guidelines do not dictate how research should be conducted. Rather, by articulating what information must be transparently reported, they indirectly encourage better research practices." The difference between "what elements to report" and "how to determine the reported elements" should be clarified in the manuscript. The focus of the paper is not clear as it cites papers making e.g., sample size recommendations while in the discussion, focusing on "what elements to report". If the message is in fine "to report how sample size was determined", then I believe that the above paper doesn't add to the current guidelines, for example.**

We agree and have added a clarification to the objective:

- Line 147 ff: "*While reporting guidelines do not prescribe how studies should be designed, conducted, or analysed, they should reflect contemporary methodological standards by ensuring that information relevant to assessing these aspects is reported transparently. Consequently, methodological developments may have implications not only for research practice itself, but also for what information should be reported to enable readers to judge whether current methodological recommendations have been followed*."
- Line 339ff: "Importantly, reporting guidelines do not dictate how research should be conducted. Rather, by articulating what information must be transparently reported, they indirectly encourage better research practices *and facilitate the assessment of adherence to contemporary methodological standards*."

#Comment 4: **It is somewhat surprising that no mathematical or statistical literature is cited in the discussion of sample sizes, as these are precisely the fields in which methodological developments would be expected.**

We have added the following citations regarding sample size estimations:

14 Bourredjem A, Cardot H, Devilliers H. Asymptotic Confidence Interval, Sample Size Formulas and Comparison Test for the Agreement Intra-Class Correlation Coefficient in Inter-Rater Reliability Studies. *Stat Med*. 2024;43:5060–76. doi: 10.1002/sim.10217

15 Lu M-J, Zhong W-H, Liu Y-X, *et al*. Sample Size for Assessing Agreement between Two Methods of Measurement by Bland−Altman Method. *Int J Biostat*. 2016;12. doi: 10.1515/ijb-2015-0039

We have also revised this paragraph. Details are provided in response to comment 9.

#Comment 5: **Related to Comment 4: It remains unclear how the quality of, for example, simulation studies will be evaluated and how the adequacy of mathematical developments will be assessed. Established guidance already exists for the design and reporting of simulation studies (e.g., the ADEMP guidelines), and such frameworks should be considered when evaluating literature. It is therefore essential that the authors explicitly describe how the quality of the included literature will be evaluated and which**

objective assessment tools or criteria will be applied, particularly given that the authors will be evaluating their own work.

The objective of this methods review is not to evaluate the methodological quality of simulation studies or to assess the adequacy of mathematical or statistical developments. Rather, the aim is to identify and synthesise methodological developments, discussions, and recommendations that may inform the revision of the GRRAS-COSMIN reporting guideline.

Because the review will include heterogeneous publication types, including methodological papers, tutorials, editorials, commentaries, guidance documents, and textbook material, we do not plan to apply a formal critical appraisal across all included sources. We have clarified this in the manuscript and added it as a limitation. Line 359: *"Second, because the review includes diverse publication types such as methodological papers, tutorials, editorials, commentaries, guidance documents, and textbook material, and because the objective is to identify methodological developments rather than evaluate the validity of these studies, no formal critical appraisal of included sources will be undertaken. Consequently, the review will describe and synthesise methodological developments discussed in the literature but will not assess the relative merit or empirical support of competing methodological approaches. "*

<u>#Comment 6</u>: The list of keywords appears rather limited. For instance, in MeSH the indexed terms include reproducibility of results and observer variation (including intraobserver and interobserver variation). The authors should therefore carefully reconsider and broaden their keyword strategy. Different scientific fields often use different terminology for closely related concepts. For example, in psychology, variance partitioning coefficients derived from generalisability theory are commonly used to study reliability. In the health sciences, terms such as precision, accuracy, repeatability, and replicability are used (some of these words are included in Appendix 2). Similarly, including variants of Cohen's kappa, such as Fleiss' kappa, Scott's pi, and similar measures would likely improve coverage of the literature search. The terminology and naming of concepts vary substantially across disciplines and many variants of Cohen's kappa and intraclass correlation coefficients were introduced over the years. The authors are invited to expand the keyword list accordingly to reduce the risk of missing relevant literature.

We thank the reviewer for this detailed and constructive comment and for engaging with our supplement in so much detail!

In response to the reviewer's suggestions, we re-evaluated and expanded the search strategy. We explored a range of additional terms, including terms related to observer variation, interrater and intrarater assessments, reproducibility, generalizability theory, repeatability, precision, accuracy, and alternative agreement coefficients. The impact of these terms on retrieval was assessed through iterative testing and examination of the retrieved records.

As a result of this process, we expanded the search strategy to include additional terms capturing interrater, intrarater, and intraobserver assessments (including variability, reproducibility, and generalizability). Other suggested terms were not retained because they either substantially reduced search specificity by retrieving large numbers of irrelevant records or did not meaningfully contribute additional relevant records beyond those already captured by the existing strategy. The updated search string is presented in Table 3.

Table3. Search string to address review question 2.

Date	Database	Search String	Hits
2026/0/02	Medline via Ovid	Ovid MEDLINE(R) ALL <1946 to June 01, 2026><br>1 reliability.kf,ti. 62933<br>2 agreement.kf,ti. 15474<br>3 measurement error.kf,ti. 2208<br>4 reproducibility of measurements.kf,ti. 104<br>5 *interrater.kf,ti. 1751*<br>6 *intrarater.kf,ti. 407*<br>*7 intraobserver.kf,ti. 982*<br>8 5 or 6 or 7 2900<br>9 Cohen's.kf,ti. 297<br>10 kappa.kf,ti. 20011<br>11 ICC.kf,ti. 1126<br>12 Gwet's.kf,ti. 18<br>13 guidance.kf,ti. 31327<br>14 guideline.kf,ti. 32524<br>15 tutorial.kf,ti. 2611<br>16 checklist.kf,ti. 9827<br>17 exp "Statistics as Topic"/ 3347331<br>18 exp "Reproducibility of Results"/ 514177<br>19 Research Design/ 135596<br>20 Publishing/ or "Access to Information"/ 39696<br>21 1 and 2 1109<br>22 3 or 4 or 8 or 9 or 10 or 11 or 12 26450<br>23 21 or 22 27336<br>24 13 or 14 or 15 or 16 75846<br>25 17 or 18 or 19 or 20 3776731<br>26 24 or 25 3843007<br>27 23 and 26 4982<br>28 limit 22 to yr="2015 - 2026" 11698<br>29 27 and 28 2301	2301

<u># Comment 7</u>: P2, line 30: "Second, we will perform a systematic search in Medline, PsycINFO, Embase, Eric, and Cinahl supplemented by key references identified by or known to the author team." It is unclear how the authors will prevent bias in the selection of "key references identified by or known to the author team." As currently phrased, this approach appears subjective and potentially prone to selection bias. The authors should therefore clarify the procedures.

The systematic database searches and citation tracking constitute the primary approach to identifying relevant literature. Additional sources identified by the author team are intended only to supplement this process, for example by capturing influential methodological resources, textbooks, guidance documents, or known key papers that may not be retrieved through database searching alone. This approach is particularly relevant for methodological literature, where terminology and indexing are often inconsistent. However, such sources will not be included solely based on personal preference. They must be directly relevant to the review questions. We will document all additional sources, including how each source was identified and the reason for inclusion. We clarified this:

- Line 204: *"These sources are intended to supplement the systematic search by providing conceptual context, identifying seminal methodological contributions, and reducing the risk of overlooking influential work that may not be retrieved through database searching alone."*

- Line 210: "*Additional sources will be included only if they are directly relevant to the review questions, and the source's identification and reason for inclusion will be documented*."

Moreover, we would like to point out that results of this methods review will inform the development of an *initial* draft of the GRRAS-COSMIN guideline for reporting studies on reliability and agreement/measurement error. This draft will subsequently be refined through a large international modified Delphi consensus process involving key stakeholders and experts with methodological, statistical, psychometric, clinimetric, and applied measurement expertise. Thus, the methods review is one component of the guideline development process and will be complemented by expert judgement and consensus when determining the final content of the reporting guideline.

# Comment 8: P2, line 34:" in the field of health measurements" and P11, line 39-40 "healthcare, psychology and education". The authors should clarify how these areas relate to one another within the context of the review and provide a clear rationale for their inclusion (and limitation).

We have revised the manuscript to resolve this inconsistency and now use the terminology "healthcare, psychology and education" consistently throughout.

# Comment 9: "At the same time, important advances have been made in areas such as sample size determination, handling of measurement error, and the appraisal of risk of bias in studies of reliability and agreement [12–14]." Related to Comment 2, the literature cited here appears highly selective and does not include references from the mathematical or statistical literature, where many of the methodological developments in these areas originate. What is the rationale to cite these three studies in particular?

We have changed the phrasing to better reflect the focus on advances in reliability, agreement, and measurement error in health, psychological, and educational research (also in line with Comment 2) and the discussions relating to these fields while retaining the references added in response to comment 4. We have reviewed the chosen references and now provide more specific examples and rationales for their use.

- Line 104 ff.: "Nevertheless, several developments and ongoing challenges highlight the need to revisit and update the existing GRRAS. Central to these are enduring conceptual ambiguities, for example, the distinction between reliability and agreement/measurement error [7,8] and the appropriate use of internal consistency measures [9]. These uncertainties have contributed to methodological debates concerning the selection and interpretation of statistical coefficients [9,10] and errors in the application and reporting of method and results. This, in turn, hampers efforts to synthesize evidence and evaluate measurement quality in systematic reviews [1,11]. *At the same time, important methodological discussions have advanced the assessment and interpretation of reliability, agreement, and measurement error in health, psychological, and educational research. These include discussions on agreement coefficients [10,12,13], recommendations for sample size determination for intraclass correlation coefficients (ICCs), κ statistics, and Bland–Altman approaches [14–17], guidance on the design, analysis, and interpretation of reliability and measurement error*

studies [2]. Commentaries on GRRAS have further emphasized the need for revision, offering constructive feedback and concrete suggestions for improvement [18,19]."

<u># Comment 10:</u> P7, line 37: I am a bit confused by the sentence. I understand that peer-reviews, textbooks and dissertations are excluded. It is unclear to me why, while websites are included as eligible sources. This choice appears difficult to justify from a methodological perspective, as the former typically undergo formal scholarly review or editorial oversight, whereas websites may vary considerably in terms of reliability and scientific rigor. The authors should clarify the rationale for this decision and explain how the credibility and quality of website sources will be ensured. Further, the authors should give objective criteria to define a website as "recognized methodological website". A last issue with website is how to monitor the date of creation of a webpage with respect to the time window of the literature review. The authors should specify how they will proceed. I think that recent dissertations could be interesting as sometimes they include chapters that are not yet published or are still under review.

Thank you for pointing this out. We agree that the rationale for including website-based resources while excluding dissertations was insufficiently explained and is inconsistent. We share the concerns raised by the reviewer and have therefore removed the inclusion of website material. In addition, we agree that dissertations may contain important methodological developments that have not yet been published elsewhere. Since we also do not exclude other non-peer reviewed scientific output (e.g., preprints), we decided to include dissertations too. The changes are reflected in Table 2.

<u># Comment 11:</u> P7, line 40: It is unclear how texts written in languages other than German or English will be handled. The authors should clarify the procedures that will be used for translation and data extraction from such sources. In particular, it would be important to specify whether professional translation, native-speaking reviewers, or automated translation tools will be used, and how the accuracy and consistency of the translations will be ensured. In a context where wording is important (see Table 1), this is a important detail.

We have clarified the translation procedures in the manuscript. Since we will rely on automated tools, we cannot guarantee accuracy and have added this to the limitation section.

- Line 177ff: "*Full texts published in languages other than English or German will be translated using DeepL Translator[36]. Data extraction will be performed based on the translated text. In addition, where substantial contributions to the findings are derived from non-English or non-German sources, a back-translation procedure will be used for key excerpts to identify potential discrepancies in meaning.*"
- Line 370ff: "*Finally, while the absence of language restrictions may reduce the risk of language bias, non-English and non-German sources will be translated using automated translation tools. Consequently, inaccuracies in translation or subtle differences in terminology and meaning may affect the interpretation of methodological concepts, definitions, or recommendations. We will seek to minimise this risk by checking key passages against the original text where necessary.*"

# Comment 12: P8, line 32-33: related to comments 2 and 9. What about statistics and mathematics?

We were unable to identify the exact sentence or section to which this comment refers since we did not include line numbers in our initial submission. However, we understand the concern as being related to the scope of the review and the extent to which mathematics and statistics are covered. We have therefore clarified the targeted literature throughout the manuscript. The review focuses on methodological developments relevant to reliability, agreement, and measurement error studies in health, psychological, and educational measurement research. This does not mean that methodological work from mathematics or statistics is excluded. Rather, statistical and biostatistical literature will be included where it is relevant to the review questions and retrieved through the search strategy or supplementary source identification. We have revised the manuscript to make this scope more explicit and to acknowledge that methodological developments published exclusively in mathematics or statistics journals may not be comprehensively captured (see also response to Comment 2 and 9).

# Comment 13: P9, line 23: "The scoping review will include additional key sources identified by the author team." It is unclear how the authors will ensure that the selection of such additional key sources is conducted in a transparent and unbiased manner (related to Comment 7). As formulated, this approach appears subjective and may introduce selection bias. The authors should therefore clarify the procedures and criteria that will guide the identification and inclusion of these sources.

We have changed our language to clarify that additional author-identified sources are used as a supplementary search method, not as a replacement for the systematic database searches, citation tracking, and reference list screening. This approach is consistent with methodological guidance for evidence synthesis searches. For example, PRISMA-S recognises "other methods" for identifying sources, including searching personal files, and emphasises that these methods should be transparently reported even when they are not fully reproducible (Rethlefsen et al., 2021).

We have revised the manuscript to clarify that all supplementary sources must be directly relevant to the review questions, will be assessed for eligibility by two reviewers, and will be documented separately, including the route of identification and reason for inclusion (see also the response to comment 7). We also added a step to the data analysis to ensure that we will be able to identify whether themes were created on systematically searched literature or personally selected literature only.

- Line 291ff: "*During synthesis, we will document whether themes are supported by systematically identified sources or arise mainly from supplementary sources.*"

# Comment 14: P9, lines 4-5: This includes articles about methodological discussions [10,33], resources from the COSMIN website and relevant textbooks. Do the materials hosted on the COSMIN website not correspond to formally published or peerreviewed work? Are textbooks excluded or included? (see page 7, line 37).

Textbooks are excluded for the forward citation analysis of GRRAS-related comments, whereas relevant textbook chapters are eligible for the second review question, as specified in Table 2. In response to Comment 10, we have removed websites as an eligible source type. COSMIN materials will only be considered where they correspond to formal guidance documents, manuals, or peer-reviewed publications that meet the eligibility criteria.

# Comment 15: P10, line 18: “discipline as indicated by the affiliation of the corresponding *author* (e.g., health sciences, psychology, education).” It is unclear why the discipline is determined specifically based on the affiliation of the corresponding author. This criterion appears arbitrary, as research papers often involve multidisciplinary teams, and the corresponding author’s affiliation may not accurately reflect the methodological orientation of the publication. The authors should justify why it is considered an appropriate proxy for classifying disciplinary background, or alternatively consider a more robust and transparent classification strategy.

We have reconsidered our strategy to make it more robust and transparent.

- Line 240ff: “*The field or discipline of each source will be classified using the OECD (Organisation for Economic Co-operation and Development) Fields of Science categories [40]. For sources indexed in Web of Science, Web of Science subject categories will be extracted and mapped to OECD categories using the InCites OECD Category Schema. For sources not indexed in Web of Science, such as textbooks, guidance documents, or other supplementary sources, classification will be based on the source content, journal or book scope, and, where informative, author departments or affiliations. Multiple fields may be assigned where appropriate, and unclear cases will be discussed within the review team.*”

#Comment 16: P10, line 56: “In other cases, the process will be more interpretative, such as determining what qualifies as a major methodological development, which will require informed judgment from the research team”. This formulation appears highly subjective and raises concerns regarding consistency and reproducibility. The authors should explicitly define objective criteria for what will be considered a “major methodological development” and specify how these criteria will be applied in practice. In addition, it is important to describe the procedures that will be used to minimise bias in these judgments.

We do not think that fully objective criteria for what constitutes a “major methodological development” would be appropriate for this qualitative synthesis. In line with qualitative analysis and qualitative evidence synthesis guidance, rigour will instead be addressed through transparent and systematic analytic procedures. We have expanded this section and clarified that we will use a structured codebook, document coding decisions, discuss uncertainties within the multidisciplinary author team, and provide examples linking themes to the underlying source material. We have also added this issue to the limitations section.

- Line 274ff: “*for example when grouping related methodological discussions into overarching categories and considering their relevance for reporting guidance. We will not apply an a priori threshold for what constitutes a “major” methodological development. Instead, the relevance and prominence of identified methodological discussions will be described transparently, considering the content of the recommendation, the type of source, whether similar issues are discussed across multiple sources, and the potential implications for reporting reliability and agreement/ measurement error studies. To enhance trustworthiness, we will use a structured codebook, document coding decisions, discuss uncertainties and changes to the*

codebook within the multidisciplinary author team, and provide examples linking synthesized themes to the underlying source material.“

- Line 365ff: “*Third, identifying and synthesising methodological developments requires a degree of interpretive judgement. This is inherent to qualitative synthesis [55]. We will address this through study selection being performed independently by two reviewers, documentation of coding decisions, reflexive discussions within the multidisciplinary author team, and transparent reporting of how themes are supported by the included sources.* “

#Comment 17: How will the authors handle publications from predatory journals or other potentially non-credible sources? The manuscript does not specify any strategy for identifying or excluding such publications. Given the potential impact of low-quality or non-peer-reviewed documents (such as websites), a strategy should be explained.

We have added criteria for assessing whether journals or publishers should be considered potentially predatory. For this purpose, we will use the checklist provided by Think. Check. Submit (https://thinkchecksubmit.org/). Sources published in journals or by publishers that raise substantial concerns according to these criteria will be discussed within the review team and reasons for in- or exclusion will be documented. This procedure has been added to the manuscript.

- Line 230ff: “*During full-text screening, we will also consider whether sources are published in journals or by publishers that raise concerns regarding editorial and publishing practices. For this purpose, we will use the Think. Check. Submit. checklist as guidance [40]. Sources for which substantial concerns arise will be discussed within the review team before a final decision is made. Reasons for in- or exclusion will be documented.“*

#Comment 18: Appendix: 11. De Vet, H. C., Terwee, C. B., Mokkink, L. B., & Knol, D. L. (2011). Measurement in medicine: a practical guide. Cambridge university press. It is unclear why this reference has been included, given that it was published before the stated search period (01.01.2015–30.06.2025). The authors should clarify the rationale for its inclusion.

The publication date restriction applies to the systematic database search to be conducted to identify recent methodological developments relevant to reliability, agreement, and measurement error studies. The additional sources identified by the author team serve a different purpose. They are included as supplementary methodological resources to provide conceptual context, identify seminal methodological contributions, and reduce the risk of overlooking influential work that may not be retrieved through database searching alone. As methodological literature often remains relevant for extended periods of time, these supplementary sources are not necessarily subject to the same publication date restrictions as the systematic search. We have clarified this distinction in the manuscript:

- Line 187: “The review will include *supplementary* sources identified by the author team. *These sources are intended to supplement the systematic search by providing conceptual context, identifying seminal methodological contributions, and reducing the risk of overlooking influential work that may not be retrieved through database searching alone.* This *may* include articles about methodological discussions [10,37]and relevant textbook

chapters *judged as important by the author team. Because these sources are intended to supplement the systematic database search, they are not necessarily subject to the same publication date restrictions. Additional sources will be included only if they are directly relevant to the review questions, and the source's identification and reason for inclusion will be documented."*

<u># Comment 19</u>: I invite the authors to explicitly discuss potential methodological limitations, including possible sources of bias, limitations in the search strategy, as well as their potential impact on the robustness and interpretability of the findings.

We have added a limitations section to the discussion.

- Line 349 ff: *"A comprehensive search strategy with no language restrictions will be employed across multiple databases to ensure thorough coverage of the literature. Nonetheless, this review has several methodological limitations that should be considered when interpreting the findings. First, the search strings are specific and focused, which may improve precision but could limit sensitivity and risk missing relevant literature. Specifically, this review seeks to identify methodological developments relevant to reliability, agreement, and measurement error in health, psychological or educational research and focuses on databases that primarily index health, psychological, and educational literature. Consequently, methodological developments published exclusively in, for example, mathematics or statistics journals may not be comprehensively captured. Second, because the review includes diverse publication types such as methodological papers, tutorials, editorials, commentaries, guidance documents, and textbook material, and because the objective is to identify methodological developments rather than evaluate the validity of these studies, no formal critical appraisal of included sources will be undertaken. Consequently, the review will describe and synthesise methodological developments discussed in the literature but will not assess the relative merit or empirical support of competing methodological approaches. Third, identifying and synthesising methodological developments requires a degree of interpretive judgement. This is inherent to qualitative synthesis [55]. We will address this through study selection being performed independently by two reviewers, documentation of coding decisions, reflexive discussions within the multidisciplinary author team, and transparent reporting of how themes are supported by the included sources. Finally, while the absence of language restrictions may reduce the risk of language bias, non-English and non-German sources will be translated using automated translation tools. Consequently, inaccuracies in translation or subtle differences in terminology and meaning may affect the interpretation of methodological concepts, definitions, or recommendations. We will seek to minimise this risk by checking key passages against the original text where necessary."*

VERSION 2 - REVIEW

**Reviewer**	**2**
**Name**	**Vanbelle, S**

**Affiliation**	**Maastricht University**
**Date**	**30-Jun-2026**
**COI**	

Dear Editor,

I appreciated the efforts of the authors to address my concerns.

I thank you for your efforts in providing detailed answers to my comments, which were addressed adequately. I only have a minor comment. I am unsure whether it is allowed to upload academic work in deepL.

VERSION 2 - AUTHOR RESPONSE

Reviewer: 2

Thank you for the positive response overall and the critical question.

We will use the DeepL Pro version only, and the translations will be used solely for our internal understanding and manuscript preparation. We will not redistribute the translated material or make it publicly available. According to DeepL's Privacy Policy, "When using our services DeepL Translator Pro, DeepL API Pro and DeepL Write Pro to translate or improve texts, the texts or documents you submit will not be permanently stored and will only be kept temporarily to the extent necessary for the production and transmission of the translation or improvement. After complete performance of the contractually agreed services all submitted texts or documents and their translations or improvements will be deleted. When using these services, your texts will not be used to improve the quality of our services" (DeepL Privacy Policy, https://www.deepl.com/en/privacy, accessed July 1, 2026). The cited DeepL Pro terms indicate that submitted texts are processed only temporarily to generate the translation, are deleted after completion of the service, and are not used to improve DeepL's services. Therefore, the use of DeepL Pro does not involve permanent storage, model training, or public dissemination of the academic works. In addition, the resulting translations will be used only internally and will not be redistributed.

We have clarified this in the manuscript:
Line 173: "English or German will be translated using DeepL Translator Pro 1[37]."
Footnote: "1According to DeepL's Privacy Policy, submitted texts are only processed temporarily to generate the translation, deleted after completion of the service, and not used to improve DeepL's services, and no translated copyrighted material was redistributed [36]."

36 DeepL Privacy Policy | Protecting Your Privacy Is Important To Us.
https://www.deepl.com/en/privacy (accessed 1 July 2026)
37 KI-Plattform von DeepL: Übersetzung, Voice und API. https://www.deepl.com (accessed 1 June 2026)
We hope that this adequately addresses the point raised by the reviewer.
