# Supplementary material for: Developments in the field of reliability and agreement studies in health, psychological and educational measurement: a methods review protocol
Source: BMJ Open. 2026 Jul 22;16(7):e109876. doi: 10.1136/bmjopen-2025-109876 (PMC13410919; doi:10.1136/bmjopen-2025-109876)
Supplement: Author's manuscript [file bmjopen-2025-109876.draft_revisions.pdf]

BMJ Open is committed to open peer review. As part of this commitment we make the peer review history of every article we publish publicly available.

When an article is published we post the peer reviewers' comments and the authors' responses online. We also post the versions of the paper that were used during peer review. These are the versions that the peer review comments apply to.

The versions of the paper that follow are the versions that were submitted during the peer review process. They are not the versions of record or the final published versions. They should not be cited or distributed as the published version of this manuscript.

BMJ Open is an open access journal and the full, final, typeset and author-corrected version of record of the manuscript is available on our site with no access controls, subscription charges or pay-per-view fees (http://bmjopen.bmj.com).

If you have any questions on BMJ Open's open peer review process please email info.bmjopen@bmj.com

BMJ Open

# Developments in the field of reliability and agreement studies: a methods review protocol



| | |
|---|---|
| Journal: | *BMJ Open* |
| Manuscript ID | bmjopen-2025-109876 |
| Article Type: | Protocol |
| Date Submitted by the Author: | 25-Aug-2025 |





| | |
|---|---|
| Complete List of Authors: | Pittelkow, M.-M.; Charite - Universitatsmedizin Berlin, Institute of Clinical Nursing Science; Berlin Institute of Health at Charite, QUEST Center for Responsible Research<br>Mönch, M; Charite - Universitatsmedizin Berlin, Institute of Biometry and Clinical Epidemiology<br>Mokkink, L.B.; VU University Medical Centre Amsterdam, Department of Epidemiology and Data Science; Amsterdam Public Health Research Institute, Methodology<br>Streiner, D.; McMaster University, Psychiatry & Behavioural Neurosciences<br>Brorson, S.; Zealand University Hospital Koge, Dept. of Orthopaedic Surgery<br>Shoukri, M.; King Faisal Specialist Hospital and Research Centre, 8 Biostatistics and Scientific Computing Department; Western University Schulich School of Medicine & Dentistry, Department of Epidemiology and Biostatistics<br>Kottner, J.; Charite - Universitatsmedizin Berlin, Institute of Clinical Nursing Science |





| | |
|---|---|
| Keywords: | STATISTICS & RESEARCH METHODS, Methods, Patient Reported Outcome Measures, Psychometrics, Systematic Review |
| | |



SCHOLARONE
MANUSCRIPTS

# Developments in the field of reliability and agreement studies: a methods review protocol

Pittelkow, M.-M.[1,2*], Maximilian Mönch[3], Mokkink, L.B. [4,5], Streiner, D. L.[6], Brorson S.[7], Shoukri M.[8,9], Kottner J.[1]

[1] Charité Universitätsmedizin Berlin, Institute of Clinical Nursing Science, Berlin, Germany

[2] QUEST Center for Responsible Research, Berlin Institute of Health at Charité Universitätsmedizin Berlin, Berlin, Germany

[3] Charité – Universitätsmedizin Berlin, corporate member of Freie Universität Berlin and Humboldt-Universität zu Berlin, Institute of Biometry and Clinical Epidemiology, Charitéplatz 1, 10117 Berlin, Germany

[4] Amsterdam UMC, Vrije Universiteit Amsterdam, Department of Epidemiology and Data Science, Amsterdam, Netherlands

[5] Amsterdam Public Health research institute, Methodology, Amsterdam, Netherlands

[6] Department of Psychiatry and Behavioural Neurosciences, McMaster University, Hamilton, Ontario, Canada

[7] Centre for Evidence-Based Orthopaedics, Zealand University Hospital and Department of Clinical Medicine, University of Copenhagen, Denmark

[8] Biostatistics and Scientific Computing Department, King Faisal Specialist Hospital and Research Centre, Riyadh, 11211, Saudi Arabia

[9] Department of Epidemiology and Biostatistics, Schulich School of Medicine and Dentistry, University of Western Ontario, London, Ontario, Canada

[*] Correspondence should be addressed to Merle-Marie Pittelkow; Charité-Universitätsmedizin Berlin, Institute of Clinical Nursing Science, Charitéplatz 1, 10117 Berlin, Germany, Email: merle-marie.pittelkow@charite.de

For peer review only

## ABSTRACT

**Introduction**: The Guidelines for Reporting Reliability and Agreement Studies (GRRAS) were developed to improve the completeness and transparency of reporting of reliability and agreement of health measurement instruments. However, since their publication in 2011 methodological standards, both for reporting guideline development and reliability, agreement, and measurement error studies, have advanced, highlighting the need for aligning and updating. In addition, related initiatives like COnsensus-based Standards for the selection of health Measurement Instruments (COSMIN) have emerged, offering opportunities to harmonize terminology and promote consistency across diverse types of measurement instruments. This methods review aims (1) to systematically identify and synthesize commentaries on and evaluations of the original GRRAS, and (2) to map recent methodological developments in the planning, conduct, and interpretation of reliability, agreement and measurement error studies that should be reflected in the reporting and may imply or explicitly recommend updates, to inform the development of the GRRAS-COSMIN reporting guidelines.

**Methods and analysis**: Two complementary search strategies will be employed. First, forward direct citation tracking of the original GRRAS publications will be conducted in Web of Science. We will include sources providing critique, commentaries, or suggestions related to the GRRAS. We will exclude publications that used GRRAS just for structuring their report, withdrawn or retracted articles, textbooks, peer reviews, supplements, and conference materials without full-text publications. Included articles will be analysed using descriptive content analysis. Second, we will perform a systematic search in Medline, PsycINFO, Embase, Eric, and Cinahl supplemented by key references identified by or known to the author team. We will include studies, reviews, commentaries, editorials, methodological papers, tutorials and guidance documents that discuss or critically reflect on methodological aspects of the measurement properties reliability and/or agreement/measurement error published between 01.01.2015 and 31.07.2025 in the field of health measurement research. Eligibility will be assessed by two independent reviewers and included sources will be analysed using 'codebook' thematic analysis. Findings will be presented narratively supplemented by visuals and tables if appropriate.

**Ethics and dissemination**: This study involves publicly available data. No ethical approval is needed. Results will be disseminated through an open-access journal publication following the Preferred Reporting Items for Systematic Reviews and Meta-Analyses-Scoping Review Extension and conference presentations.

***Keywords:*** reliability, agreement, measurement error, reporting guidelines, methods review

## STRENGTH AND LIMITATIONS:

- This methods review protocol adheres to the methodological guidance provided by the Joanna Briggs Institute for conducting scoping reviews.
- Two complementary search strategies, each aligned with a specific review aim, are used, followed by tailored data synthesis for each.
- No language restrictions will be applied.
- A comprehensive search strategy is employed across multiple databases to ensure thorough coverage of the literature; however, the search strings are specific and focused, which may

For peer review only

improve precision but could limit sensitivity and risk missing relevant but less directly indexed literature.

- Grey literature will not be searched systematically; however, relevant sources identified by the authors will be included where possible.

For peer review only

## INTRODUCTION

In clinical, psychological or educational practice and research, obtained data (e.g. blood pressure, values of standardised pain scales, evaluations of exams) or diagnoses (e.g. risk of falling, melanoma yes/no) must be reliable. Reliability and agreement/measurement error are fundamental properties of measurement instruments, including patient-reported outcome measures (PROMs), performance-based instruments, diagnostic test results, clinician-reported outcomes, or lab results. While reliability and agreement are often used interchangeably, they reflect conceptually distinct constructs. Definitions vary slightly (see Table 1), and for the purposes of this review, we adopt the following working definitions: *Reliability* refers to the ability of a measurement instrument to distinguish between individuals or objects [1,2]. It is operationalised as the ratio of variability between subjects (e.g., participants) to the total variability of all measurements in the sample. *Agreement*, also referred to as measurement error, is defined as the degree to which scores or ratings of a test or instrument are identical [1,2].

**Table 1. Selected examples of definitions of reliability and agreement as reported in the literature**

| | Reliability | Agreement/ Measurement error/ Error of measurement |
|---|---|---|
| COnsensus-based Standards for the selection of health Measurement Instruments (COSMIN) [3] | "the proportion of the total variance in the measurements which is due to the true difference between patients" (p. 1) [3] | "The systematic and random error of a patient's score that is not attributed to the true changes in the construct to be measured." (p.1) [3] |
| Guidelines for Reporting Reliability and Agreement Studies (GRRAS)[1] | "the ratio of variability between subjects (e.g., patients) or objects (e.g., computed tomography scans) to the total variability of all measurements in the sample" (p. 1) [1]<br>"the ability of a measurement to differentiate between subjects or objects" (p.1) [1] | "the degree to which scores or ratings are identical" (p.1) [1] |
| Standards for Educational and Psychological Testing [4] | "The degree to which test scores for a group of test takers are consistent over repeated applications of a measurement procedure and hence are inferred to be dependable and consistent for an individual test taker; the degree to which scores are free of random errors of measurement for a given group". [4] | "The difference between an observed score and the corresponding true score". [4] |

Since reliability and agreement/measurement error are essential for judging the quality of measurement instruments, and by extension directly impact the quality of research and practice, their transparent reporting is crucial. Only what is transparently reported can be critically assessed, interpreted, and built upon. To improve the quality, transparency, and consistency of reporting reliability, agreement and measurement error studies, a multidisciplinary working group developed the Guidelines for Reporting Reliability and Agreement Studies (GRRAS), published in 2011 [1,5]. Reporting guidelines - defined as "a checklist, flow diagram, or structured text to guide authors in

For peer review only

reporting a specific type of research, developed using explicit methodology" [6] - are recognised as valuable for enhancing the transparency and completeness of study reporting across various research designs. By articulating key elements to be addressed, such guidelines enhance transparency, support interpretability, and facilitate replicability and synthesis. Since its publication, GRRAS has been cited extensively and remains a frequently used reference for reporting reliability and agreement studies.

Nevertheless, several developments and ongoing challenges highlight the need to revisit and update the existing GRRAS. Central to these are enduring conceptual ambiguities, for example, the distinction between reliability and agreement/measurement error, the implications of relative versus absolute measurement error, and the appropriate use of internal consistency measures [7–9]. These uncertainties have contributed to methodological debates concerning the selection and interpretation of statistical coefficients [8,10] and errors in the application and reporting of method and results. This, in turn, hampers efforts to synthesize evidence and evaluate measurement quality in systematic reviews [1,11]. At the same time, important advances have been made in areas such as sample size determination, handling of measurement error, and the appraisal of risk of bias in studies of reliability and agreement [12–14]. Commentaries on GRRAS have further emphasized the need for revision, offering constructive feedback and concrete suggestions for improvement [15,16].

In parallel, related initiatives have emerged, offering opportunities for conceptual and terminological alignment. Notably, the COnsensus-based Standards for the selection of health Measurement INstruments (COSMIN) developed several guidelines for conducting and reporting studies on measurement properties of PROMs [17] and for conducting and reporting systematic reviews on outcome measurement instruments [18,19], including a tool to assess the risk of bias in studies of reliability and agreement/measurement error [20]. While COSMIN initially focused risk of bias assessments of existing studies on any measurement property, and GRRAS targets specifically on reporting of studies on reliability and agreement, both frameworks address shared constructs. Harmonizing these efforts holds potential to improve clarity, promote consistency in terminology, and enhance the usability and uptake of reporting standards. Similarly, the Quality Appraisal tool for studies of Diagnostic Reliability (QAREL), developed by Lucas et al. [21], provides a structured framework for evaluating methodological quality in diagnostic reliability studies and is broadly applicable to reliability and agreement research.

In the related but different field of diagnostic accuracy, the Standards for Reporting of Diagnostic Accuracy Studies (STARD) statement [22,23] offers a comprehensive set of recommendations to ensure transparent and complete reporting, while the Quality Assessment of Diagnostic Accuracy Studies (QUADAS) tool [24,25] provides a structured approach for evaluating risk of bias of diagnostic accuracy studies included in systematic reviews.

Reporting and risk of bias assessment are inherently interdependent: only what is reported can be critically appraised. Therefore, reporting guidelines must explicitly include the elements targeted by risk of bias tools to ensure meaningful and comprehensive evaluation. In response to these developments and ongoing challenges, our multidisciplinary research group set out to develop the GRRAS-COSMIN guidelines, an updated and extended version of the original GRRAS.

**Objective**

The objective of this methods review is to systematically identify and summarize published comments, evaluations, and methodological discussions relevant to the development of the new GRRAS-COSMIN

For peer review only

reporting guidelines for studies on reliability, agreement, and measurement error. This process aligns with the methodological standards for reporting guideline development and revisions established by the EQUATOR Network [26].

Specifically, this review will examine literature published in the last decade that either explicitly critiques or evaluates the existing GRRAS, or that provides methodological recommendations, advancements, or reflections to inform best practice reporting of reliability, agreement and measurement error studies. This includes issues related to the planning, conduct, analysis, and interpretation of such studies.

Specific review questions are:

1. What comments and/or evaluations have been published about GRRAS?
2. What major methodological discussions, recommendations, and/or advancements have been made in the last decade concerning the design, conduct, analysis, and reporting of reliability, and agreement/measurement error studies?

## METHODS

This protocol of a methods review is informed by the best practice guidelines for the development of scoping review protocols developed by the Joanna Briggs Institute [27] as well as relevant guidance for conducting methods reviews [28–31]. Results reporting will follow an adapted version of the Preferred Reporting Items for Systematic Reviews and Meta-Analyses-Scoping Review Extension [32]. To address the two review aims, we will implement two complementary search strategies.

### Eligibility criteria

To address review question 1, we will include peer-reviewed articles, commentaries, or other scholarly publications that cite one or both of the original GRRAS publications [1,5] and provide critique, commentaries, or suggestions related to the GRRAS guideline. We will exclude articles that cite GRRAS solely to structure their report; withdrawn or retracted articles; textbooks; peer-reviews; supplements; dissertations; and conference abstracts, posters, or slide presentations without accompanying full-text publications. No language restrictions will be applied. Texts in languages other than German or English will be translated.

In- and exclusion criteria to address review question 2 following the Population, Concept, and Context (PCC) scheme are displayed in Table 2.

**Table 2.** In- and exclusion criteria for review question 2; structure adapted from [28].

| | Inclusion criteria | Exclusion criteria |
|---|---|---|
| **Types of documents** | • Studies, reviews, commentaries, editorials, methodological papers, and guidance documents that explicitly discuss, evaluate, reflect on, or propose methodological aspects of the measurement properties reliability and/or agreement and/or measurement error | • Empirical studies investigating measurement properties without critical methodological reflection.<br>• Reviews summarizing measurement properties without methodological discussion.<br>• Conference abstracts, posters, slide presentations without full text.<br>• Errata, retracted or withdrawn articles. |

For peer review only - http://bmjopen.bmj.com/site/about/guidelines.xhtml

| | | |
|---|---|---|
| | • Relevant textbook chapters and material from recognized methodological websites (e.g., COSMIN.nl).<br>• Published between 01.01.2015 and 31.07.2025. | |
| **Types of information** | • Narrative discussions, recommendations, and proposals related to study design, planning, conduct, analysis, reporting, and interpretation of reliability/agreement studies.<br>• Text on measurement error (absolute and relative), reliability/agreement statistics (e.g., ICC, kappa, generalizability studies), and risk of bias appraisal. | • Purely empirical numeric results on measurement properties without methodological reflection<br>• Data on internal consistency/homogeneity.<br>• Data on reliability of automated systems. |
| **Types of methods** | • Methods and approaches for designing, conducting, analysing, and reporting reliability, agreement, and measurement error studies.<br>• Methods for appraising risk of bias in such studies. | • Methods addressing automated systems or engineering applications only. |
| **Types of disciplines** | • Health research, psychology and education | • Engineering; economics |

**Information sources**

To address review question 1, we will search Web of Science (WoS), which offers a broad and well-curated citation index across disciplines.

To address review question 2, we will consecutively search the databases Embase and Medline via OVID and PsycINFO, Eric, and Cinahl via EBSCOhost. These databases were selected based on two considerations: first, a search in the Information Matrix for the Analysis of Journals (MIAR; https://miar.ub.edu) to identify databases indexing the journals of key references; and second, their relevance for covering the fields we aim to include in this review. In addition, we will search the Charité Library (via Ex Libris Primo) for relevant books and book chapters.

**Search strategy**

To address review question 1, we will conduct direct forward citation tracking of the two original GRRAS guideline publications [1,5]. To this end, we will search WoS for all articles citing the original publications. A pilot search on July 1st 2025 resulted in 1557 citations for reference [1], and 441 citations for reference [5] respectively.

To address review question 2, the search string shown in Table 3 will be used with publication year restricted between 1.1.2015 to 30.6.2025. A detailed account of the development of the search string

For peer review only

is documented in the supplement. The search string was developed in Medline via Ovid and will be translated to the other databases. The scoping review will include additional key sources identified by the author team. This includes articles about methodological discussions [10,33], resources from the COSMIN website and relevant textbooks. A list of additional sources is presented in the supplement. This list is not exhaustive, and additional sources will be added during the search. Lastly, reference lists of all included articles will be screened for additional relevant sources using the SpiderCite tool from the Systematic Review (SR) Accelerator [34].

**Table3.** Search string to address review question 2.

| Date | Database | Search String | Hits |
|---|---|---|---|
| 2025/07/23 | Medline via Ovid | Ovid MEDLINE(R) ALL <1946 to July 22, 2025><br>1 reliability.kf,ti. 58836<br>2 agreement.kf,ti. 14479<br>3 measurement error.kf,ti. 2066<br>4 reproducibility of measurements.kf,ti. 104<br>5 Cohen's.kf,ti. 273<br>6 kappa.kf,ti. 19344<br>7 ICC.kf,ti. 1040<br>8 Gwet's.kf,ti. 16<br>9 guidance.kf,ti. 29096<br>10 guideline.kf,ti. 29764<br>11 tutorial.kf,ti. 2382<br>12 checklist.kf,ti. 9181<br>13 exp "Statistics as Topic"/ 3252455<br>14 exp "Reproducibility of Results"/ 499563<br>15 Research Design/ 132229<br>16 Publishing/ or "Access to Information"/39294<br>17 1 and 2 1006<br>18 3 or 4 or 5 or 6 or 7 or 8 or 17 23646<br>19 9 or 10 or 11 or 12 70055<br>20 13 or 14 or 15 or 16 3669295<br>21 19 or 20 3730226<br>22 18 and 21 3075<br>23 limit 22 to yr="2015 - 2025" 1736 | 1736 |

## Data management and selection process

The retrieved records will be exported from the respective databases in XML format. For deduplication and screening and dispute we will use the tools provided by the SR Accelerator [34,35], a web-based platform designed to support systematic review workflows, per review question. First, duplicate entries will be identified and removed prior to screening. Two reviewers (MMP and MM) will then independently assess titles and abstracts. Full texts of potentially relevant studies will be retrieved and independently assessed against the eligibility criteria. If no full text can be extracted using

For peer review only - http://bmjopen.bmj.com/site/about/guidelines.xhtml

standard methods (e.g., journal websites), we will (1) contact the library or (2) contact the corresponding author. Corresponding authors will be contacted twice with a follow-up after two weeks of no reply following the first contact.

Reasons for exclusion during full text screening will be documented. Any disagreements will be resolved through discussions, with a third reviewer (JK) consulted in cases where consensus cannot be reached.

**Data collection process and data items**

The first author (MMP) will extract the following, descriptive information from all included full text: title, author, publication year, country of the corresponding author, publication type, language, and field or discipline as indicated by the affiliation of the corresponding author (e.g., health sciences, psychology, education), and research question(s) or topic as stated by the authors (if available).

Contextual information will be generated during data synthesis described below.

**Risk of bias in individual studies**

A critical appraisal of the evidence quality will not be conducted, since the included publication types are expected to be mostly opinion pieces which do not lend themselves to systematic quality assessment geared towards empirical studies [27].

**Data synthesis**

All included full-text publications will be uploaded to MAXQDA 24 [36] and coded by the first author (MMP). Different qualitative analytic approaches will be employed to address each research question.

To answer review question 1, we will employ qualitative content analysis using a directed approach [37]. The analysis is planned as deductive and descriptive, focusing on manifest content (i.e., what the text says instead of underlying meaning of the text [38]. Unit of analysis will be the individual sources. This approach is well-suited to systematically categorise and summarise clearly defined information, and thus is appropriate given our primary focus on descriptive and structured content, specifically how the GRRAS guidelines have been discussed or critiqued in the literature. Analysis will occur at the semantic level, emphasizing explicit statements, which will be organized into descriptive categories and summarized narratively. Prespecified codes include the type of measurement property addressed, definitions of reliability and agreement (if provided), the specific GRRAS items discussed, recommendations for revision such as proposed new items or clarifications, and commentary on the strengths and weaknesses of the original GRRAS.

To answer review question 2, we will use hybrid 'codebook' thematic analysis (TA) combining both deductive and inductive coding. We will use 'codebook' TA, defined by Braun and Clarke as a cluster of methods that use structured coding frameworks (e.g., codebooks), where themes are typically identified early but may be refined through further analysis [40]. This approach allows to begin with a set of a priori categories, while remaining open to inductively derived insights and aligns with our aim to describe the *main* developments, recurrent themes, and topics in the field. In some cases, extracting relevant content will be relatively straightforward—for example, documenting key concepts from included tutorials that illustrate standard approaches. In other cases, the process will be more interpretative, such as determining what qualifies as a major methodological development, which will require informed judgment from the research team.

For peer review only - http://bmjopen.bmj.com/site/about/guidelines.xhtml

The initial codebook will be structured around the following deductive codes, derived from our research objectives: measurement property addressed (e.g., reliability, agreement, measurement error); methodological issues discussed (e.g., study design recommendations, statistical methods, handling of measurement error (absolute/relative), risk of bias in reliability/agreement studies, reporting quality); whether existing frameworks are referenced (e.g., COSMIN, QAREL, GRRAS, STARD); and definitions (e.g., agreement, reliability). As coding progresses, inductive codes will be developed to capture ideas or concepts not anticipated in the initial framework.

Results will be synthesized descriptively. Study characteristics will be presented in tabular and graphical formats and summarized narratively in the text. For review question 1, we will map which specific GRRAS items have been commented on, identify proposals for their revision and clarifications. For review question 2, we will narratively summarize the key methodological developments identified through our hybrid 'codebook' TA, focusing on aspects related to the design, conduct, reporting, and interpretation of reliability and agreement studies. These findings may be organized into overarching categories such as conceptual discussions, practical recommendations, and methodological advancements. Furthermore, we aim to identify generated themes not currently addressed by GRRAS that may inform future development of the GRRAS-COSMIN guideline.

## ETHICS AND DISSEMINATION

This study will involve publicly available data. No ethical approval is needed. Results will be disseminated through an open-access journal publication and national and international conference presentations. Data curation and the analysis will be documented and openly accessible from the Open Science Framework.

**Patient and public involvement**

Patients and/or the public were not involved in this research's design, conduct, reporting or dissemination plans.

## DISCUSSION

Reliability and agreement/measurement error are fundamental measurement properties in healthcare, psychology, and education [9,20,41–45]. Transparent, accurate, and complete reporting of studies evaluating these properties is essential for the appropriate interpretation and application of measurement instruments. This methods review constitutes the first step in a structured revision of the GRRAS, informed by recent conceptual and methodological developments and conducted in accordance with established standards for reporting guideline development, as outlined by the EQUATOR Network [6]. The results will inform the development of an initial draft of the GRRAS-COSMIN guideline for reporting studies on reliability and agreement/measurement error, which will subsequently be refined through a modified Delphi consensus process involving key stakeholders and experts.

Well-developed reporting guidelines serve as key tools for enhancing both the reporting and design of research. They provide structured guidance on the minimum essential information to be included when reporting specific types of studies, thereby supporting completeness, clarity, and transparency [46]. Although their primary function is to assist authors during manuscript preparation [47], the influence of reporting guidelines goes beyond. When considered early, for instance during study protocol development, reporting guidelines can support improved study planning. When followed during reporting, these guidelines ensure that readers have sufficient information to assess the

For peer review only - http://bmjopen.bmj.com/site/about/guidelines.xhtml

methodological quality of the research – in the case of GRRAS the reliability and agreement/measurement error properties of a given measurement instrument.

Improved reporting also benefits systematic reviewers, developers of clinical practice guidelines, and those conducting electronic literature searches [48]. Complete and standardized reporting is crucial for the conduct of meta-analyses and systematic reviews. Incomplete or inconsistent reporting of pose significant barriers to the synthesis of findings across studies [46]. In the field of reliability, agreement and measurement studies this limits the ability to appraise the quality of measurement instruments and hampers the development of cumulative evidence bases. Complete and standardized reporting is thus critical not only for individual study interpretation and informed decision-making but also for the advancement of measurement science more broadly.

Importantly, reporting guidelines do not dictate how research should be conducted. Rather, by articulating what information must be transparently reported, they indirectly encourage better research practices. For example, inadequate reporting in clinical trials may result in inflated confidence in the efficacy of an intervention. Similarly, insufficient detail in studies of reliability and agreement can lead to incorrect conclusions about measurement properties. In both cases, the downstream consequences include misguided clinical decision-making and reduced reproducibility. Over time, the widespread use of rigorously developed reporting guidelines, such as the GRRAS-COSMIN reporting guideline, has the potential to enhance research quality by fostering greater methodological awareness and transparency across disciplines.

Taken together, this methods review will provide a critical foundation for updating GRRAS into GRRAS-COSMIN reporting guideline. By mapping current reporting practices in reliability, agreement, and measurement error studies, it aims to support the development of updated reporting guidelines that foster transparency and reproducibility and synthesis these studies.

For peer review only

## References

1 Kottner J, Audigé L, Brorson S, *et al.* Guidelines for Reporting Reliability and Agreement Studies (GRRAS) were proposed. *J Clin Epidemiol*. 2011;64:96–106. doi: 10.1016/j.jclinepi.2010.03.002

2 Mokkink LB, Eekhout I, Boers M, *et al.* Studies on Reliability and Measurement Error of Measurements in Medicine - From Design to Statistics Explained for Medical Researchers. *Patient Relat Outcome Meas*. 2023;14:193–212. doi: 10.2147/PROM.S398886

3 COSMIN definitions of domains, measurement properties, and aspects of measurement properties. https://cosmin.nl/wp-content/uploads/COSMIN-definitions-domains-measurement-properties.pdf (accessed 19 August 2025)

4 American Educational Research Association, American Psychological Association, National Council on Measurement in Education. *Standards for educational and psychological testing*. Washington, DC: American Educational Research Association 2014.

5 Kottner J, Audige L, Brorson S, *et al.* Guidelines for Reporting Reliability and Agreement Studies (GRRAS) were proposed. *Int J Nurs Stud*. 2011;48:661–71. doi: 10.1016/j.ijnurstu.2011.01.016

6 What is a reporting guideline? | EQUATOR Network. https://www.equator-network.org/about-us/what-is-a-reporting-guideline/ (accessed 3 June 2025)

7 Kottner J, Streiner DL. The difference between reliability and agreement. *J Clin Epidemiol*. 2011;64:701–2. doi: 10.1016/j.jclinepi.2010.12.001

8 Kottner J, Streiner DL. Internal consistency and Cronbach's *α*: A comment on Beeckman et al. (2010). *Int J Nurs Stud*. 2010;47:926–8. doi: 10.1016/j.ijnurstu.2009.12.018

9 Streiner DL. Statistics Commentary Series: Commentary #15—Reliability. *J Clin Psychopharmacol*. 2016;36:305. doi: 10.1097/JCP.0000000000000517

10 Vach W, Gerke O. Gwet's AC1 is not a substitute for Cohen's kappa – A comparison of basic properties. *MethodsX*. 2023;10:102212. doi: 10.1016/j.mex.2023.102212

11 Van Tiggelen H, Kottner J, Campbell K, *et al.* Measurement properties of classifications for skin tears: A systematic review. *Int J Nurs Stud*. 2020;110:103694. doi: 10.1016/j.ijnurstu.2020.103694

12 Cairney J, Streiner DL. Using relative improvement over chance (RIOC) to examine agreement between tests: Three case examples using studies of developmental coordination disorder (DCD) in children. *Res Dev Disabil*. 2011;32:87–92. doi: 10.1016/j.ridd.2010.09.001

13 Mokkink LB, de Vet H, Diemeer S, *et al.* Sample size recommendations for studies on reliability and measurement error: an online application based on simulation studies. *Health Serv Outcomes Res Methodol*. 2023;23:241–65. doi: 10.1007/s10742-022-00293-9

14 Hong H, Choi Y, Hahn S, *et al.* Nomogram for sample size calculation on a straightforward basis for the kappa statistic. *Ann Epidemiol*. 2014;24:673–80. doi: 10.1016/j.annepidem.2014.06.097

15 Oster C, Schoo A, Litt J, *et al.* Supporting workforce practice change: protocol for a pilot study of a motivational interviewing virtual client software tool for health professionals. *BMJ OPEN*. 2020;10. doi: 10.1136/bmjopen-2019-033080

For peer review only - http://bmjopen.bmj.com/site/about/guidelines.xhtml

16 Gerke O, Möller S, Debrabant B, *et al.* Experience Applying the Guidelines for Reporting Reliability and Agreement Studies (GRRAS) Indicated Five Questions Should Be Addressed in the Planning Phase from a Statistical Point of View. *Diagnostics*. 2018;8:69. doi: 10.3390/diagnostics8040069

17 Gagnier J, de Arruda G, Terwee C, *et al.* COSMIN reporting guideline for studies on measurement properties of patient-reported outcome measures: version 2.0. *Qual LIFE Res*. 2025;34:1901–11. doi: 10.1007/s11136-025-03950-x

18 Elsman E, Mokkink L, Terwee C, *et al.* Guideline for reporting systematic reviews of outcome measurement instruments (OMIs): PRISMA-COSMIN for OMIs 2024. *J PATIENT-Rep OUTCOMES*. 2024;8. doi: 10.1186/s41687-024-00727-7

19 Mokkink LB, Elsman EBM, Terwee CB. COSMIN guideline for systematic reviews of patient-reported outcome measures version 2.0. *Qual Life Res*. 2024;33:2929–39. doi: 10.1007/s11136-024-03761-6

20 Mokkink LB, Boers M, Vleuten CPM van der, *et al.* COSMIN Risk of Bias tool to assess the quality of studies on reliability or measurement error of outcome measurement instruments: a Delphi study. *BMC Med Res Methodol*. 2020;20. doi: 10.1186/s12874-020-01179-5

21 Lucas NP, Macaskill P, Irwig L, *et al.* The development of a quality appraisal tool for studies of diagnostic reliability (QAREL). *J Clin Epidemiol*. 2010;63:854–61. doi: 10.1016/j.jclinepi.2009.10.002

22 Cohen JF, Korevaar DA, Altman DG, *et al.* STARD 2015 guidelines for reporting diagnostic accuracy studies: explanation and elaboration. *BMJ Open*. 2016;6:e012799. doi: 10.1136/bmjopen-2016-012799

23 Bossuyt PM, Reitsma JB, Bruns DE, *et al.* STARD 2015: an updated list of essential items for reporting diagnostic accuracy studies. *BMJ*. 2015;351:h5527. doi: 10.1136/bmj.h5527

24 Whiting PF, Rutjes AWS, Westwood ME, *et al.* QUADAS-2: a revised tool for the quality assessment of diagnostic accuracy studies. *Ann Intern Med*. 2011;155:529–36. doi: 10.7326/0003-4819-155-8-201110180-00009

25 Whiting P, Rutjes AW, Reitsma JB, *et al.* The development of QUADAS: a tool for the quality assessment of studies of diagnostic accuracy included in systematic reviews. *BMC Med Res Methodol*. 2003;3:25. doi: 10.1186/1471-2288-3-25

26 Moher D, Schulz KF, Simera I, *et al.* Guidance for Developers of Health Research Reporting Guidelines. *PLOS Med*. 2010;7:e1000217. doi: 10.1371/journal.pmed.1000217

27 Peters MDJ, Godfrey C, McInerney P, *et al.* Best practice guidance and reporting items for the development of scoping review protocols. *JBI Evid Synth*. 2022;20:953. doi: 10.11124/JBIES-21-00242

28 Clarke M. Guide to the contents of a Cochrane Methodology protocol and review.

29 Aguinis H, Ramani RS, Alabduljader N. Best-Practice Recommendations for Producers, Evaluators, and Users of Methodological Literature Reviews. *Organ Res Methods*. 2023;26:46–76. doi: 10.1177/1094428120943281

For peer review only

30 Hirt J, Ewald H, Briel M, *et al.* Searching a methods topic: practical challenges and implications for search design. *J Clin Epidemiol*. 2024;166:111201. doi: 10.1016/j.jclinepi.2023.10.017

31 Gentles SJ, Charles C, Nicholas DB, *et al.* Reviewing the research methods literature: principles and strategies illustrated by a systematic overview of sampling in qualitative research. *Syst Rev*. 2016;5:172. doi: 10.1186/s13643-016-0343-0

32 Tricco AC, Lillie E, Zarin W, *et al.* PRISMA Extension for Scoping Reviews (PRISMA-ScR): Checklist and Explanation. *Ann Intern Med*. 2018;169:467–73. doi: 10.7326/M18-0850

33 Vet HCW de, Mokkink LB, Terwee CB, *et al.* Clinicians are right not to like Cohen's κ. *BMJ*. 2013;346:f2125. doi: 10.1136/bmj.f2125

34 Clark J, Glasziou P, Del Mar C, *et al.* A full systematic review was completed in 2 weeks using automation tools: a case study. *J Clin Epidemiol*. 2020;121:81–90. doi: 10.1016/j.jclinepi.2020.01.008

35 Forbes C, Greenwood H, Carter M, *et al.* Automation of duplicate record detection for systematic reviews: Deduplicator. *Syst Rev*. 2024;13:206. doi: 10.1186/s13643-024-02619-9

36 MAXQDA Software für qualitative Datenanalyse. 1989.

37 Hsieh H-F, Shannon SE. Three approaches to qualitative content analysis. *Qual Health Res*. 2005;15:1277–88. doi: 10.1177/1049732305276687

38 Graneheim UH, Lundman B. Qualitative content analysis in nursing research: concepts, procedures and measures to achieve trustworthiness. *Nurse Educ Today*. 2004;24:105–12. doi: 10.1016/j.nedt.2003.10.001

39 10.2.8 Analysis of the evidence - JBI Manual for Evidence Synthesis - JBI Global Wiki. https://jbi-global-wiki.refined.site/space/MANUAL/355862791/10.2.8+Analysis+of+the+evidence (accessed 3 July 2025)

40 Braun V, and Clarke V. One size fits all? What counts as quality practice in (reflexive) thematic analysis? *Qual Res Psychol*. 2021;18:328–52. doi: 10.1080/14780887.2020.1769238

41 De Vet HCW, Terwee CB, Mokkink LB, *et al. Measurement in Medicine: A Practical Guide*. 1st ed. Cambridge University Press 2011.

42 Streiner DL, Kottner J. Recommendations for reporting the results of studies of instrument and scale development and testing. *J Adv Nurs*. 2014;70:1970–9. doi: 10.1111/jan.12402

43 Streiner DL, Norman GR, Cairney J. *Health Measurement Scales: A practical guide to their development and use*. Sixth Edition, Sixth Edition. Oxford, New York: Oxford University Press 2024.

44 Prinsen CAC, Vohra S, Rose MR, *et al.* How to select outcome measurement instruments for outcomes included in a "Core Outcome Set" – a practical guideline. *Trials*. 2016;17:449. doi: 10.1186/s13063-016-1555-2

45 Kottner J., Blume-Peytavi U. Reliability and agreement of instrumental skin barrier measurements in clinical pressure ulcer prevention research. *Int Wound J*. 2021;18:716 EP – 727. doi: 10.1111/iwj.13574

For peer review only

46 Importance of Transparent Reporting of Health Research. *Guidelines for Reporting Health Research: A User's Manual*. Wiley 2014:1–13.

47 Simera I, Altman DG. Writing a research article that is "fit for purpose": EQUATOR Network and reporting guidelines. *Ann Intern Med*. 2009;151:JC2-2. doi: 10.7326/0003-4819-151-4-200908180-02002

48 Using Reporting Guidelines Effectively to Ensure Good Reporting of Health Research. *Guidelines for Reporting Health Research: A User's Manual*. Wiley 2014:32–40.

**Author contributions:** D.L.S.: Funding acquisition, Methodology, and Writing - review & editing. J.K.: Conceptualization, Funding acquisition, Methodology, Project administration, Supervision, and Writing - review & editing. L.B.M.: Funding acquisition, Methodology, and Writing - review & editing. M.-M.P.: Conceptualization, Data curation, Methodology, Project administration, Resources, and Writing - original draft. M.M.: Data curation, Methodology, and Writing - review & editing. M.S.: Funding acquisition, Methodology, and Writing - review & editing. S.B.: Funding acquisition, Methodology, and Writing - review & editing.

**Funding statement:** The project is funded by the Deutsche Forschungsgemeinschaft (DFG, German Research Foundation) – 560535232.

**Competing Interest Statement:** The authors have no conflict of interest to declare.

**Acknowledgement:** We thank Corinna Dressler, PhD; Medical Library Charité-Universitätsmedizin Berlin for her support in the development of the search strateg

For peer review only

**Supplement of: Recent developments in the field of reliability and agreement studies: protocol for a scoping review of methodological literature**

Pittelkow, M.-M., Maximilian M., Mokkink, L.B., Streiner, D. L., Brorson S., Shoukri M., Kottner J.

This supplement details the sample references and search string development corresponding to review question 2 of the protocol "*Recent developments in the field of reliability and agreement studies: protocol for a scoping review of methodological literature*".

**Review question 2**: What major methodological discussions, recommendations, and/or advancements have been made in the last decade concerning the design, conduct, analysis, and reporting of reliability, and agreement/measurement error studies?

**Sample references**

This is a preliminary list of key references to be included in the scoping review in addition to the sources identified via the systematic search. Please note that this list is not exhaustive and sources may be added during the conduct of the review.

**Journal articles**

1. Borg, D. N., Bach, A. J. E., O'Brien, J. L., & Sainani, K. L. (2022). Calculating sample size for reliability studies. PM & R : The Journal of Injury, Function, and Rehabilitation, 14(8), 1018–1025. https://doi.org/10.1002/pmrj.12850
2. Cohen JF, Korevaar DA, Altman DG, Bruns DE, Gatsonis CA, Hooft L, Irwig L, Levine D, Reitsma JB, de Vet HC, Bossuyt PM. STARD 2015 guidelines for reporting diagnostic accuracy studies: explanation and elaboration. BMJ Open. 2016 Nov 14;6(11):e012799. https://doi.org/10.1136/bmjopen-2016-012799.
3. Koo, T. K., & Li, M. Y. (2016). A Guideline of Selecting and Reporting Intraclass Correlation Coefficients for Reliability Research. Journal of Chiropractic Medicine, 15(2), 155–163. https://doi.org/10.1016/j.jcm.2016.02.012
4. Mokkink, L. B., Boers, M., van der Vleuten, C. P. M., Bouter, L. M., Alonso, J., Patrick, D. L., de Vet, H. C. W., & Terwee, C. B. (2020). COSMIN Risk of Bias tool to assess the quality of studies on reliability or measurement error of outcome measurement instruments: A Delphi study. BMC Medical Research Methodology, 20(1), 293. https://doi.org/10.1186/s12874-020-01179-5
5. Mokkink, L. B., de Vet, H., Diemeer, S., & Eekhout, I. (2023). Sample size recommendations for studies on reliability and measurement error: An online application based on simulation studies. Health Services and Outcomes Research Methodology, 23(3), 241–265. https://doi.org/10.1007/s10742-022-00293-9



For peer review only

6. Mokkink, L. B., Eekhout, I., Boers, M., van der Vleuten, C. P. M., & de Vet, H. C. W. (2023). Studies on Reliability and Measurement Error of Measurements in Medicine – From Design to Statistics Explained for Medical Researchers. Patient Related Outcome Measures, 14, 193–212. https://doi.org/10.2147/PROM.S398886
7. Oster, N. V., Carney, P. A., Allison, K. H., Weaver, D. L., Reisch, L. M., Longton, G., Onega, T., Pepe, M., Geller, B. M., Nelson, H. D., Ross, T. R., Tosteson, A. N. A., & Elmore, J. G. (2013). Development of a diagnostic test set to assess agreement in breast pathology: Practical application of the Guidelines for Reporting Reliability and Agreement Studies (GRRAS). BMC Women's Health, 13(1), 3. https://doi.org/10.1186/1472-6874-13-3
8. Stratford, P., Spadoni, G. F., Kuspinar, A., & Macedo, L. (2025). Considerations when designing, analyzing, and reporting reliability studies. Brazilian Journal of Physical Therapy, 29(3), 101193. https://doi.org/10.1016/j.bjpt.2025.101193
9. Vach, W., & Gerke, O. (2023). Gwet's AC1 is not a substitute for Cohen's kappa – A comparison of basic properties. MethodsX, 10, 102212. https://doi.org/10.1016/j.mex.2023.102212
10. Vet, H. C. W. de, Mokkink, L. B., Terwee, C. B., Hoekstra, O. S., & Knol, D. L. (2013). Clinicians are right not to like Cohen's κ. BMJ, 346, f2125. https://doi.org/10.1136/bmj.f2125

**Textbooks**

11. De Vet, H. C., Terwee, C. B., Mokkink, L. B., & Knol, D. L. (2011). *Measurement in medicine: a practical guide*. Cambridge university press.
12. American Educational Research Association, American Psychological Association, & National Council on Measurement in Education. (2014). Standards for educational and psychological testing. American Educational Research Association.
13. Streiner, D. L., Norman, G. R., & Cairney, J. (2024). *Health Measurement Scales: A practical guide to their development and use* (Sixth Edition, Sixth Edition). Oxford University Press.

**Search String Development**

The search string was developed with both sensitivity (maximizing the identification of relevant articles) and feasibility (retrieving a manageable number of records for screening by two reviewers) in mind. Finding this balance can be especially challenging for reviews of methods topics [1]. We started by compiling a reference sample based on PubMed IDs of key journal articles (i.e., 28137831**,** 27330520**,** 37448975**,** 23379630**,** 27464308**,** 40022968**,** 37234937**,** 23585065**,** 33267819**,** 35596122). These articles serve as examples of the types of methodological literature we aim to identify and are not necessarily expected to be fully captured by the final search string. For example, we decided a priori to limit the search to publications from the last decade (1.1.2015 to 30.6.2025), since our aim is to cover recent

methodological discussions and advancements. As a result, some of the articles included in the reference sample may not appear in the search output due to their earlier publication date.

In a first step, we identified relevant search terms and Medical Subject Headings (MeSH) terms. To this end, we (1) used the Systematic Review Accelerator (SR-Accelerator) [2] to extract and review frequently used words in the key references (see table S1) and (2) reviewed the assigned MeSH terms for the reference sample using the Yale Mesh Analyzer (1, see table S2). We additionally consulted the term finder in Ovid for MeSH terms related to reliability, agreement, or measurement error as well as the Thesaurus for Health Research Methods from the Library of Guidance for Health Scientists [4].

For peer review only

*Table S1. Word frequencies in title, abstract, and keywords of key references.*

| word | unique | title | abstract | keywords | points |
|---|---|---|---|---|---|
| reliability | 6 | 5 | 18 | 0 | 23 |
| agreement | 4 | 3 | 18 | 0 | 21 |
| measurement | 3 | 4 | 12 | 0 | 16 |
| study | 3 | 1 | 14 | 0 | 15 |
| icc | 2 | 0 | 14 | 0 | 14 |
| studies | 5 | 6 | 7 | 0 | 13 |
| between | 5 | 1 | 10 | 0 | 11 |
| cohen's | 2 | 1 | 9 | 0 | 10 |
| reporting | 4 | 4 | 6 | 0 | 10 |
| methods | 3 | 1 | 8 | 0 | 9 |
| kappa | 1 | 1 | 8 | 0 | 9 |
| ac1 | 1 | 1 | 8 | 0 | 9 |
| design | 4 | 0 | 9 | 0 | 9 |
| gwet's ac1 | 1 | 0 | 8 | 0 | 8 |
| gwet's | 1 | 0 | 8 | 0 | 8 |
| error | 2 | 2 | 6 | 0 | 8 |
| measure | 1 | 0 | 7 | 0 | 7 |
| outcome measurement | 1 | 1 | 6 | 0 | 7 |
| outcome | 1 | 1 | 6 | 0 | 7 |
| cohen's kappa | 1 | 0 | 7 | 0 | 7 |
| measurements | 2 | 0 | 7 | 0 | 7 |
| form | 1 | 0 | 6 | 0 | 6 |
| values | 4 | 0 | 6 | 0 | 6 |
| test | 4 | 2 | 4 | 0 | 6 |
| repeated | 2 | 0 | 6 | 0 | 6 |
| measurement error | 2 | 2 | 4 | 0 | 6 |
| size | 2 | 1 | 5 | 0 | 6 |
| sample | 2 | 1 | 5 | 0 | 6 |
| when | 3 | 1 | 5 | 0 | 6 |
| κ | 1 | 1 | 4 | 0 | 5 |
| article | 1 | 0 | 5 | 0 | 5 |
| instrument | 1 | 0 | 5 | 0 | 5 |
| assess | 2 | 2 | 3 | 0 | 5 |
| rate | 1 | 0 | 5 | 0 | 5 |
| researchers | 3 | 0 | 5 | 0 | 5 |
| ratings | 3 | 0 | 5 | 0 | 5 |
| different | 4 | 0 | 5 | 0 | 5 |
| scores | 2 | 0 | 5 | 0 | 5 |
| correlation | 2 | 1 | 4 | 0 | 5 |
| e | 3 | 0 | 5 | 0 | 5 |
| based | 3 | 1 | 4 | 0 | 5 |
| raters | 4 | 0 | 5 | 0 | 5 |
| information | 4 | 1 | 4 | 0 | 5 |
| results | 3 | 0 | 5 | 0 | 5 |
| sample size | 2 | 1 | 4 | 0 | 5 |
| purpose | 1 | 0 | 5 | 0 | 5 |
| quantity | 1 | 0 | 4 | 0 | 4 |
| clinicians | 1 | 1 | 3 | 0 | 4 |
| diagnostic | 2 | 2 | 2 | 0 | 4 |
| research | 3 | 1 | 3 | 0 | 4 |
| reached | 1 | 0 | 4 | 0 | 4 |
| consensus | 1 | 0 | 4 | 0 | 4 |
| variation | 2 | 0 | 4 | 0 | 4 |
| how | 1 | 0 | 4 | 0 | 4 |

| word | unique | title | abstract | keywords | points |
|---|---|---|---|---|---|
| measurement instruments | 1 | 1 | 3 | 0 | 4 |
| instruments | 1 | 1 | 3 | 0 | 4 |
| quality | 1 | 1 | 3 | 0 | 4 |
| agreement rate | 1 | 0 | 4 | 0 | 4 |
| positive | 2 | 0 | 4 | 0 | 4 |
| properties | 2 | 1 | 3 | 0 | 4 |
| developed | 3 | 0 | 4 | 0 | 4 |
| k | 1 | 0 | 4 | 0 | 4 |
| repeated measurements | 1 | 0 | 4 | 0 | 4 |
| patients | 2 | 0 | 4 | 0 | 4 |
| application | 2 | 2 | 2 | 0 | 4 |
| calculation | 3 | 0 | 4 | 0 | 4 |
| discussion | 3 | 0 | 4 | 0 | 4 |
| provide | 3 | 0 | 4 | 0 | 4 |
| study purpose | 1 | 0 | 4 | 0 | 4 |
| however | 4 | 0 | 4 | 0 | 4 |
| observations | 1 | 1 | 2 | 0 | 3 |
| same | 2 | 0 | 3 | 0 | 3 |
| cohen’s κ | 1 | 1 | 2 | 0 | 3 |
| readers | 1 | 0 | 3 | 0 | 3 |
| appropriate | 2 | 0 | 3 | 0 | 3 |
| icc form | 1 | 0 | 3 | 0 | 3 |
| guidelines | 2 | 2 | 1 | 0 | 3 |
| breast | 1 | 1 | 2 | 0 | 3 |
| diagnostic test | 2 | 2 | 1 | 0 | 3 |
| word | unique | title | abstract | keywords | points |
| reported | 3 | 0 | 3 | 0 | 3 |
| standards | 1 | 0 | 3 | 0 | 3 |
| measurement instrument | 1 | 0 | 3 | 0 | 3 |
| tool | 2 | 1 | 2 | 0 | 3 |
| both | 2 | 0 | 3 | 0 | 3 |
| paper | 3 | 0 | 3 | 0 | 3 |
| using | 3 | 0 | 3 | 0 | 3 |
| agreement between | 2 | 1 | 2 | 0 | 3 |
| basic | 2 | 1 | 2 | 0 | 3 |
| most | 2 | 0 | 3 | 0 | 3 |
| mse | 1 | 0 | 3 | 0 | 3 |
| parameters | 2 | 0 | 3 | 0 | 3 |
| about | 3 | 0 | 3 | 0 | 3 |
| intraclass correlation | 2 | 1 | 2 | 0 | 3 |
| intraclass | 2 | 1 | 2 | 0 | 3 |
| number | 2 | 0 | 3 | 0 | 3 |
| online application | 1 | 1 | 2 | 0 | 3 |
| online | 1 | 1 | 2 | 0 | 3 |
| whether | 2 | 0 | 3 | 0 | 3 |
| reliability study | 3 | 0 | 3 | 0 | 3 |
| reliability studies | 2 | 1 | 2 | 0 | 3 |
| reporting reliability | 2 | 2 | 1 | 0 | 3 |

For peer review only

Table S2. Results from the Yale MeSH Analyzer

| **PMID** | 28137831 | 27330520 | 37448975 | 23379630 | 27464308 | 40022968 | 37234937 | 23585065 | 33267819 |
|---|---|---|---|---|---|---|---|---|---|
| **MeSH Headings** | Advisory Committees* | | | | | | | | |
| | Bias<br>Biomedical Research | | | Breast / pathology<br>Breast Diseases / pathology*<br>Breast Neoplasms / classification*<br>Breast Neoplasms / diagnosis<br>Breast Neoplasms / pathology* | | | | | Bias |
| | Checklist* | | | | | | | | Consensus |
| | Diagnostic Techniques and Procedures / standards* | | | Diagnosis, Differential | Diagnostic Tests, Routine / standards* | | | | Delphi Technique* |
| | | | | Female | | | | | |
| | | | | | Guidelines as Topic | | | | |
| | Humans | | | Humans | Humans | Humans | | Humans | Humans |
| | | | | Observer Variation | | | | Observer Variation* | |

(continued)

For peer review only

Table S2 continued. Results from the Yale MeSH Analyzer

| **PMID** | 28137831 | 27330520 | 37448975 | 23379630 | 27464308 | 40022968 | 37234937 | 23585065 | 33267819 |
|---|---|---|---|---|---|---|---|---|---|
| | Publishing | | | Predictive Value of Tests | Psychology / standards<br>Psychometrics / standards*<br>Publishing / standards* | | | Probability* | |
| | | | | Quality Assurance, Health Care / standards* | | | | | |
| | Research Design | | | Reproducibility of Results<br>Research Design / standards | | Reproducibility of Results<br>Research Design* | | Reproducibility of Results*<br>Research Design* | Reproducibility of Results |
| | | | | Sensitivity and Specificity | Societies, Scientific | Sample Size | | | |
| | Terminology as Topic | | | | | | | | |
| **Author Assigned Keywords** | Diagnostic accuracy<br>Medical publishing<br>Peer review<br>Reporting quality<br>Research waste<br>Sensitivity and specificity | Reliability and validity<br>Research<br>Statistics | classical test theory<br>generalizability theory<br>intraclass correlation coefficient<br>measurement error<br>reliability<br>standard error of measurement | | | Measurement Methods<br>Rehabilitation<br>Reliability<br>Research design<br>Statistics | Agreement<br>Gwet's AC1<br>Method comparison<br>Observer<br>Rater<br>Reliability<br>Repeatability<br>Reproducibility | | COSMIN<br>Delphi study<br>Measurement error<br>Outcome measurement instruments<br>Quality assessment<br>Reliability<br>Risk of Bias |

For peer review only

For peer review only - http://bmjopen.bmj.com/site/about/guidelines.xhtml

From this, we constructed a set of possible search and MeSH terms (see table S3).

**Table S3. Potential search and MeSH terms. MeSH terms are retrieved from Medline via Ovid**

| Search concept | Potential search terms | |
|---|---|---|
| **Target:**<br><br>**Methodological studies, reviews, commentaries, editorials, and guidance documents that explicitly discuss, evaluate, reflect on, or propose methodological aspects of reliability, agreement, and/or measurement error.** | Reliability | Method* |
| | reproducibility of measurements | Outcome |
| | Agreement | Cohen's |
| | Bland Altmann analysis | Kappa |
| | Measurement | ICC |
| | Measurement error | AC1 |
| | Measure* | gwet's |
| | Stud* | Guidance |
| | Reporting | How to |
| | Reporting quality | Best practice |
| | MeSH: Research Design / standards | MeSH: Statistics as Topic |
| | MeSH: Reproducibility of Results | MeSH: Reproducibility of Results |
| | MeSH: Publishing / standards* | MeSH: Methods / Research Design |

We then iteratively piloted the search terms in Medline via OVID to develop a combination that was both sensitive and resulted in a feasible number of hits. To check the sensitivity of our search, we searched for the reference sample in the hits of the search string before adding the publication date restriction.

## References

1 Hirt J, Ewald H, Briel M, *et al.* Searching a methods topic: practical challenges and implications for search design. *J Clin Epidemiol*. 2024;166:111201. doi: 10.1016/j.jclinepi.2023.10.017

2 Clark J, Glasziou P, Del Mar C, *et al.* A full systematic review was completed in 2 weeks using automation tools: a case study. *J Clin Epidemiol*. 2020;121:81–90. doi: 10.1016/j.jclinepi.2020.01.008

3 Yale MeSH Analyzer. https://mesh.med.yale.edu/ (accessed 23 July 2025)

4 Hirt J, Schönenberger CM, Ewald H, *et al.* Introducing the Library of Guidance for Health Scientists (LIGHTS): A Living Database for Methods Guidance. *JAMA Netw Open*. 2023;6:e2253198. doi: 10.1001/jamanetworkopen.2022.53198

For peer review only

BMJ Open

# Developments in the field of reliability and agreement studies in health, psychological, and educational measurement: a methods review protocol

| | |
|---|---|
| Journal: | *BMJ Open* |
| Manuscript ID | bmjopen-2025-109876.R1 |
| Article Type: | Protocol |
| Date Submitted by the Author: | 15-Jun-2026 |
| Complete List of Authors: | Pittelkow, M.-M.; Charité – University Medical Center Berlin, corporate member of Freie Universität Berlin and Humboldt-Universität zu Berlin, Institute of Clinical Nursing Science; Berlin Institute of Health at Charité, Universitätsmedizin Berlin, QUEST Center for Responsible Research<br>Mönch, M; Charité - Universitätsmedizin Berlin, corporate member of Freie Universität Berlin and Humboldt-Universität zu Berlin, Institute of Biometry and Clinical Epidemiology<br>Mokkink, L.B.; Amsterdam UMC, Vrije Universiteit Amsterdam, Department of Epidemiology and Data Science; Amsterdam Public Health Research Institute, Methodology<br>Streiner, D.; McMaster University, Department of Psychiatry and Behavioural Neurosciences<br>Brorson, S.; University of Copenhagen, Centre for Evidence-Based Orthopaedics, Zealand University Hospital and Department of Clinical Medicine<br>Shoukri, M.; King Faisal Specialist Hospital and Research Centre, Biostatistics and Scientific Computing Department; University of Western Ontario, Department of Epidemiology and Biostatistics, Schulich School of Medicine and Dentistry<br>Kottner, J.; Charité – University Medical Center Berlin, corporate member of Freie Universität Berlin and Humboldt-Universität zu Berlin, Institute of Clinical Nursing Science |
| <b>Primary Subject Heading</b>: | Research methods |
| Secondary Subject Heading: | Diagnostics |
| Keywords: | STATISTICS & RESEARCH METHODS, Methods, Patient Reported Outcome Measures, Psychometrics, Systematic Review |
| | |

SCHOLARONE MANUSCRIPTS

# Developments in the field of reliability and agreement studies in health, psychological, and educational measurement: a methods review protocol

Pittelkow, M.-M.[1,2*], Maximilian Mönch[3], Mokkink, L.B. [4,5], Streiner, D. L.[6], Brorson S.[7], Shoukri M.[8,9], Kottner J.[1]

[1] Charité – University Medical Center Berlin, corporate member of Freie Universität Berlin and Humboldt-Universität zu Berlin, Institute of Clinical Nursing Science, Berlin, Germany

[2] Berlin Institute of Health at Charité – Universitätsmedizin Berlin, QUEST Center for Responsible Research, Berlin, Germany

[3] Charité – Universitätsmedizin Berlin, corporate member of Freie Universität Berlin and Humboldt-Universität zu Berlin, Institute of Biometry and Clinical Epidemiology, Charitéplatz 1, 10117 Berlin, Germany

[4] Amsterdam UMC, Vrije Universiteit Amsterdam, Department of Epidemiology and Data Science, Amsterdam, Netherlands

[5] Amsterdam Public Health research institute, Methodology, Amsterdam, Netherlands

[6] Department of Psychiatry and Behavioural Neurosciences, McMaster University, Hamilton, Ontario, Canada

[7] Centre for Evidence-Based Orthopaedics, Zealand University Hospital and Department of Clinical Medicine, University of Copenhagen, Denmark

[8] Biostatistics and Scientific Computing Department, King Faisal Specialist Hospital and Research Centre, Riyadh, 11211, Saudi Arabia

[9] Department of Epidemiology and Biostatistics, Schulich School of Medicine and Dentistry, University of Western Ontario, London, Ontario, Canada

[*] Correspondence should be addressed to Merle-Marie Pittelkow; Charité-Universitätsmedizin Berlin, Institute of Clinical Nursing Science, Charitéplatz 1, 10117 Berlin, Germany, Email: merle-marie.pittelkow@charite.de

For peer review only

## ABSTRACT

**Introduction**: The Guidelines for Reporting Reliability and Agreement Studies (GRRAS) were developed to improve the completeness and transparency of reporting of reliability and agreement of health measurement instruments. However, since their publication in 2011 methodological standards, both for reporting guideline development and reliability, agreement, and measurement error studies, have advanced, highlighting the need for aligning and updating. In addition, related initiatives like COnsensus-based Standards for the selection of health Measurement Instruments (COSMIN) have emerged, offering opportunities to harmonize terminology and promote consistency across diverse types of measurement instruments. This methods review aims (1) to systematically identify and synthesize commentaries on and evaluations of the original GRRAS, and (2) to map recent methodological developments in the planning, conduct, and interpretation of reliability, agreement and measurement error studies in health science, psychology and education that should be reflected in the reporting, to inform the development of the GRRAS-COSMIN reporting guidelines.

**Methods and analysis**: Two complementary search strategies will be employed. First, forward direct citation tracking of the original GRRAS publications will be conducted in Web of Science. We will include sources providing critique, commentaries, or suggestions related to the GRRAS. We will exclude publications that used GRRAS just for structuring their report, withdrawn or retracted articles, textbooks, peer reviews, supplements, and conference materials without full-text publications. Included articles will be analysed using descriptive content analysis. Second, we will perform a systematic search in Medline, PsycINFO, Embase, Eric, and Cinahl supplemented by key references identified by or known to the author team. We will include studies, reviews, commentaries, editorials, methodological papers, tutorials and guidance documents that discuss or critically reflect on methodological aspects of the measurement properties reliability and/or agreement/measurement error published between 01.01.2015 and 30.06.2026 in health sciences, psychology, or education. Eligibility will be assessed by two independent reviewers and included sources will be analysed using 'codebook' thematic analysis. Findings will be presented narratively supplemented by visuals and tables if appropriate.

**Ethics and dissemination**: This study involves publicly available data. No ethical approval is needed. Results will be disseminated through an open-access journal publication following the Preferred Reporting Items for Systematic Reviews and Meta-Analyses-Scoping Review Extension and conference presentations.


***Keywords:*** reliability, agreement, measurement error, reporting guidelines, methods review

## STRENGTH AND LIMITATIONS:

- This methods review protocol adheres to the methodological guidance provided by the Joanna Briggs Institute for conducting scoping reviews.
- Two complementary search strategies, each aligned with a specific review aim, are used, followed by tailored data synthesis for each.
- No language restrictions will be applied.
- A comprehensive search strategy is employed across multiple databases to ensure thorough coverage of the literature; however, the search strings are specific and focused, which may

For peer review only

improve precision but could limit sensitivity, especially for methodological literature that is inconsistently indexed or published outside health, psychology, and education databases.

- Grey literature will not be searched systematically; however, database searches will be supplemented by transparently documented additional sources.

For peer review only

## INTRODUCTION

In clinical, psychological or educational practice and research, obtained data (e.g. blood pressure, values of standardised pain scales, personality tests, evaluations of exams) or diagnoses (e.g. risk of falling, depression, melanoma yes/no) must be reliable. Reliability and agreement/measurement error are fundamental properties of health measurement instruments, including patient-reported outcome measures (PROMs), performance-based instruments, diagnostic test results, clinician-reported outcomes, or lab results. While reliability and agreement are often used interchangeably, they reflect conceptually distinct constructs. Definitions vary slightly (see Table 1), and for the purposes of this review, we adopt the following working definitions: *Reliability* refers to the ability of a measurement instrument to distinguish between individuals or objects [1,2]. It is operationalised as the ratio of variability between subjects (e.g., participants) to the total variability of all measurements in the sample. *Agreement*, also referred to as measurement error, is defined as the degree to which scores or ratings of a test or instrument are identical [1,2].

**Table 1. Selected examples of definitions of reliability and agreement as reported in the literature**

| | **Reliability** | **Agreement/ Measurement error/ Error of measurement** |
|---|---|---|
| COnsensus-based Standards for the selection of health Measurement Instruments (COSMIN) [3] | "the proportion of the total variance in the measurements which is due to the true difference between patients" (p. 1) [3] | "The systematic and random error of a patient's score that is not attributed to the true changes in the construct to be measured." (p.1) [3] |
| Guidelines for Reporting Reliability and Agreement Studies (GRRAS)[1] | "the ratio of variability between subjects (e.g., patients) or objects (e.g., computed tomography scans) to the total variability of all measurements in the sample" (p. 1) [1]<br>"the ability of a measurement to differentiate between subjects or objects" (p.1) [1] | "the degree to which scores or ratings are identical" (p.1) [1] |
| Standards for Educational and Psychological Testing [4] | "The degree to which test scores for a group of test takers are consistent over repeated applications of a measurement procedure and hence are inferred to be dependable and consistent for an individual test taker; the degree to which scores are free of random errors of measurement for a given group". [4] | "The difference between an observed score and the corresponding true score". [4] |

Since reliability and agreement/measurement error are essential for judging the quality of measurement instruments, and by extension directly impact the quality of research and practice, their transparent reporting is crucial. Only what is transparently reported can be critically assessed, interpreted, and built upon. To improve the quality, transparency, and consistency of reporting reliability, agreement and measurement error studies, a multidisciplinary working group developed the Guidelines for Reporting Reliability and Agreement Studies (GRRAS), published in 2011 [1,5]. Reporting guidelines - defined as "a checklist, flow diagram, or structured text to guide authors in

reporting a specific type of research, developed using explicit methodology" [6] - are recognised as valuable for enhancing the transparency and completeness of study reporting across various research designs. By articulating key elements to be addressed, such guidelines enhance transparency, support interpretability, and facilitate replicability and synthesis. Since its publication, GRRAS has been cited extensively and remains a frequently used reference for reporting reliability and agreement studies.

Nevertheless, several developments and ongoing challenges highlight the need to revisit and update the existing GRRAS. Central to these are enduring conceptual ambiguities, for example, the distinction between reliability and agreement/measurement error [7,8] and the appropriate use of internal consistency measures [9]. These uncertainties have contributed to methodological debates concerning the selection and interpretation of statistical coefficients [9,10] and errors in the application and reporting of method and results. This, in turn, hampers efforts to synthesize evidence and evaluate measurement quality in systematic reviews [1,11]. At the same time, important methodological discussions have advanced the assessment and interpretation of reliability, agreement, and measurement error in health, psychological, and educational research. These include discussions on agreement coefficients [10,12,13], recommendations for sample size determination for intraclass correlation coefficients (ICCs), κ statistics, and Bland–Altman approaches [14–17], guidance on the design, analysis, and interpretation of reliability and measurement error studies [2]. Commentaries on GRRAS have further emphasized the need for revision, offering constructive feedback and concrete suggestions for improvement [18,19].

In parallel, related initiatives have emerged, offering opportunities for conceptual and terminological alignment. Notably, the COnsensus-based Standards for the selection of health Measurement INstruments (COSMIN) developed several guidelines for conducting and reporting studies on measurement properties of PROMs [20] and for conducting and reporting systematic reviews on outcome measurement instruments [21,22], including a tool to assess the risk of bias in studies of reliability and agreement/measurement error [23]. While COSMIN initially focused risk of bias assessments of existing studies on any measurement property, and GRRAS targets specifically on reporting of studies on reliability and agreement, both frameworks address shared constructs. Harmonizing these efforts holds potential to improve clarity, promote consistency in terminology, and enhance the usability and uptake of reporting standards. Similarly, the Quality Appraisal tool for studies of Diagnostic Reliability (QAREL), developed by Lucas et al. [24], provides a structured framework for evaluating methodological quality in diagnostic reliability studies and is broadly applicable to reliability and agreement research.

In the related but different field of diagnostic accuracy, the Standards for Reporting of Diagnostic Accuracy Studies (STARD) statement [25,26] offers a comprehensive set of recommendations to ensure transparent and complete reporting, while the Quality Assessment of Diagnostic Accuracy Studies (QUADAS) tool [27,28] provides a structured approach for evaluating risk of bias of diagnostic accuracy studies included in systematic reviews.

Reporting and risk of bias assessment are inherently interdependent: only what is reported can be critically appraised. Therefore, reporting guidelines must explicitly include the elements targeted by risk of bias tools to ensure meaningful and comprehensive evaluation. In response to these developments and ongoing challenges, our multidisciplinary research group set out to develop the GRRAS-COSMIN guidelines, an updated and extended version of the original GRRAS.

**Objective**

For peer review only

The objective of this methods review is to systematically identify and summarize published comments, evaluations, and methodological discussions relevant to the development of the new GRRAS-COSMIN reporting guidelines for studies on reliability, agreement, and measurement error. While reporting guidelines do not prescribe how studies should be designed, conducted, or analysed, they should reflect contemporary methodological standards by ensuring that information relevant to assessing these aspects is reported transparently. Consequently, methodological developments may have implications not only for research practice itself, but also for what information should be reported to enable readers to judge whether current methodological recommendations have been followed. This process aligns with the methodological standards for reporting guideline development and revisions established by the EQUATOR Network [29].

Specifically, this review will examine literature published in the last decade that either explicitly critiques or evaluates the existing GRRAS, or that provides methodological recommendations, advancements, or reflections to inform best practice reporting of reliability, agreement and measurement error studies in health sciences, psychology, education. This includes issues related to the planning, conduct, analysis, and interpretation of such studies.

Specific review questions are:

1. What comments and/or evaluations have been published about GRRAS?
2. What major methodological discussions, recommendations, and/or advancements have been made in the last decade concerning the design, conduct, analysis, and reporting of reliability, and agreement/measurement error studies?

## METHODS

This protocol of a methods review is informed by the best practice guidelines for the development of scoping review protocols developed by the Joanna Briggs Institute [30] as well as relevant guidance for conducting methods reviews [31–34]. Results reporting will follow an adapted version of the Preferred Reporting Items for Systematic Reviews and Meta-Analyses-Scoping Review Extension [35]. To address the two review aims, we will implement two complementary search strategies.

### Eligibility criteria

To address review question 1, we will include peer-reviewed articles, commentaries, or other scholarly publications that cite one or both of the original GRRAS publications [1,5] and provide critique, commentaries, or suggestions related to the GRRAS guideline. We will exclude articles that cite GRRAS solely to structure their report; withdrawn or retracted articles; textbooks; peer-reviews; supplements; and conference abstracts, posters, or slide presentations without accompanying full-text publications. No language restrictions will be applied. Full texts published in languages other than English or German will be translated using DeepL Translator [36]. Data extraction will be performed based on the translated text. In addition, where substantial contributions to the findings are derived from non-English or non-German sources, a back-translation procedure will be used for key excerpts to identify potential discrepancies in meaning.

In- and exclusion criteria to address review question 2 following the Population, Concept, and Context (PCC) scheme are displayed in Table 2.

**Table 2.** In- and exclusion criteria for review question 2; structure adapted from [31].

| | Inclusion criteria | Exclusion criteria |
|---|---|---|

| | | |
|---|---|---|
| **Types of documents** | • Studies, reviews, commentaries, editorials, methodological papers, dissertations, and guidance documents that explicitly discuss, evaluate, reflect on, or propose methodological aspects of the measurement properties reliability and/or agreement and/or measurement error<br>• Relevant textbook chapters. Published between 01.01.2015 and 30.06.2026. | • Empirical studies investigating measurement properties without critical methodological reflection.<br>• Reviews summarizing measurement properties without methodological discussion.<br>• Conference abstracts, posters, slide presentations without full text.<br>• Errata, retracted or withdrawn articles. |
| **Types of information** | • Narrative discussions, recommendations, and proposals related to study design, planning, conduct, analysis, reporting, and interpretation of reliability/agreement studies.<br>• Text on measurement error (absolute and relative), reliability/agreement statistics (e.g., ICC, kappa, generalizability studies), and risk of bias appraisal. | • Purely empirical numeric results on measurement properties without methodological reflection<br>• Data on internal consistency/homogeneity.<br>• Data on reliability of automated systems. |
| **Types of methods** | • Methods and approaches for designing, conducting, analysing, and reporting reliability, agreement, and measurement error studies.<br>• Methods for appraising risk of bias in such studies. | • Methods addressing automated systems or engineering applications only. |
| **Types of disciplines** | • Health research, psychology and education | • Engineering; economics |

For peer review only

**Information sources**

To address review question 1, we will search Web of Science (WoS), which offers a broad and well-curated citation index across disciplines.

To address review question 2, we will consecutively search the databases Embase and Medline via OVID and PsycINFO, Eric, and Cinahl via EBSCOhost. These databases were selected based on two considerations: first, a search in the Information Matrix for the Analysis of Journals (MIAR; https://miar.ub.edu) to identify databases indexing the journals of key references; and second, their relevance for covering the fields we aim to include in this review. In addition, we will search the Charité Library (via Ex Libris Primo) for relevant books and book chapters.

**Search strategy**

To address review question 1, we will conduct direct forward citation tracking of the two original GRRAS guideline publications [1,5]. To this end, we will search WoS for all articles citing the original publications. A pilot search on July 1st 2025 resulted in 1557 citations for reference [1], and 441 citations for reference [5] respectively.

To address review question 2, the search string shown in Table 3 will be used with publication year restricted between 1.1.2015 to 30.6.2026. A detailed account of the development of the search string is documented in the supplement (Table S1-S3). The search string was developed in Medline via Ovid and will be translated to the other databases. The review will include supplementary sources identified by the author team. These sources are intended to supplement the systematic search by providing conceptual context, identifying seminal methodological contributions, and reducing the risk of overlooking influential work that may not be retrieved through database searching alone. This may include articles about methodological discussions [10,37]and relevant textbook chapters judged as important by the author team. Because these sources are intended to supplement the systematic database search, they are not necessarily subject to the same publication date restrictions. Additional sources will be included only if they are directly relevant to the review questions, and the source's identification and reason for inclusion will be documented. A list of additional sources is presented in the supplement. This list is not exhaustive, and additional sources will be added during the search. Lastly, reference lists of all included articles will be screened for additional relevant sources using the SpiderCite tool from the Systematic Review (SR) Accelerator [38].

**Table3.** Search string to address review question 2.

| Date | Database | Search String | Hits |
|---|---|---|---|
| 2026/0/02 | Medline via Ovid | Ovid MEDLINE(R) ALL <1946 to June 01, 2026> | 2301 |
| | | 1 reliability.kf,ti. 62933 | |
| | | 2 agreement.kf,ti. 15474 | |
| | | 3 measurement error.kf,ti. 2208 | |
| | | 4 reproducibility of measurements.kf,ti. 104 | |
| | | 5 interrater.kf,ti. 1751 | |
| | | 6 intrarater.kf,ti. 407 | |
| | | 7 intraobserver.kf,ti. 982 | |
| | | 8 5 or 6 or 7 2900 | |
| | | 9 Cohen's.kf,ti. 297 | |
| | | 10 kappa.kf,ti. 20011 | |
| | | 11 ICC.kf,ti. 1126 | |
| | | 12 Gwet's.kf,ti. 18 | |
| | | 13 guidance.kf,ti. 31327 | |
| | | 14 guideline.kf,ti. 32524 | |
| | | 15 tutorial.kf,ti. 2611 | |
| | | 16 checklist.kf,ti. 9827 | |
| | | 17 exp "Statistics as Topic"/ 3347331 | |
| | | 18 exp "Reproducibility of Results"/ 514177 | |
| | | 19 Research Design/ 135596 | |

For peer review only

| | | | | |
|---|---|---|---|---|
| | | 20 | Publishing/ or "Access to Information"/39696 | |
| | | 21 | 1 and 2 1109 | |
| | | 22 | 3 or 4 or 8 or 9 or 10 or 11 or 12 26450 | |
| | | 23 | 21 or 22 27336 | |
| | | 24 | 13 or 14 or 15 or 16 75846 | |
| | | 25 | 17 or 18 or 19 or 20 3776731 | |
| | | 26 | 24 or 25 3843007 | |
| | | 27 | 23 and 26 4982 | |
| | | 28 | limit 22 to yr="2015 - 2026" 11698 | |
| | | 29 | 27 and 28 2301 | |

**Data management and selection process**

The retrieved records will be exported from the respective databases in XML format. For deduplication and screening and dispute we will use the tools provided by the SR Accelerator [38,39], a web-based platform designed to support systematic review workflows, per review question. First, duplicate entries will be identified and removed prior to screening. Two reviewers (MMP and MM) will then independently assess titles and abstracts. Full texts of potentially relevant studies will be retrieved and independently assessed against the eligibility criteria. If no full text can be extracted using standard methods (e.g., journal websites), we will (1) contact the library or (2) contact the corresponding author. Corresponding authors will be contacted twice with a follow-up after two weeks of no reply following the first contact. Reasons for exclusion during full text screening will be documented. Any disagreements will be resolved through discussions, with a third reviewer (JK) consulted in cases where consensus cannot be reached.

During full-text screening, we will also consider whether sources are published in journals or by publishers that raise concerns regarding editorial and publishing practices. For this purpose, we will use the Think. Check. Submit. checklist as guidance [40]. Sources for which substantial concerns arise will be discussed within the review team before a final inclusion decision is made. Reasons for exclusion will be documented.

**Data collection process and data items**

The first author (MMP) will extract the following, descriptive information from all included full text: title, author, publication year, country of the corresponding author, publication type, language, and field or discipline, and research question(s) or topic as stated by the authors (if available). The field or discipline of each source will be classified using the OECD (Organisation for Economic Co-operation and Development) Fields of Science categories [41]. For sources indexed in Web of Science, Web of Science subject categories will be extracted and mapped to OECD categories using the InCites OECD Category Schema. For sources not indexed in Web of Science, such as textbooks, guidance documents, or other supplementary sources, classification will be based on the source content, journal or book scope, and, where informative, author departments or affiliations. Multiple fields may be assigned where appropriate, and unclear cases will be discussed within the review team. Contextual information will be generated during data synthesis described below.

**Risk of bias in individual studies**

For peer review only

A critical appraisal of the evidence quality will not be conducted, since the included publication types are expected to be mostly opinion pieces which do not lend themselves to systematic quality assessment geared towards empirical studies [30].

**Data synthesis**

All included full-text publications will be uploaded to MAXQDA 24 [42] and coded by the first author (MMP). Different qualitative analytic approaches will be employed to address each research question.

To answer review question 1, we will employ qualitative content analysis using a directed approach [43]. The analysis is planned as deductive and descriptive, focusing on manifest content (i.e., what the text says instead of underlying meaning of the text [44]. Unit of analysis will be the individual sources. This approach is well-suited to systematically categorise and summarise clearly defined information, and thus is appropriate given our primary focus on descriptive and structured content, specifically how the GRRAS guidelines have been discussed or critiqued in the literature. Analysis will occur at the semantic level, emphasizing explicit statements, which will be organized into descriptive categories and summarized narratively. Prespecified codes include the type of measurement property addressed, definitions of reliability and agreement (if provided), the specific GRRAS items discussed, recommendations for revision such as proposed new items or clarifications, and commentary on the strengths and weaknesses of the original GRRAS.

To answer review question 2, we will use hybrid ‘codebook’ thematic analysis (TA) combining both deductive and inductive coding. We will use ‘codebook’ TA, defined by Braun and Clarke as a cluster of methods that use structured coding frameworks (e.g., codebooks), where themes are typically identified early but may be refined through further analysis [46]. This approach allows to begin with a set of a priori categories, while remaining open to inductively derived insights and aligns with our aim to describe the *main* developments, recurrent themes, and topics in the field. In some cases, extracting relevant content will be relatively straightforward—for example, documenting key concepts from included tutorials that illustrate standard approaches. In other cases, the process will be more interpretative, for example when grouping related methodological discussions into overarching categories and considering their relevance for reporting guidance. We will not apply an a priori threshold for what constitutes a “major” methodological development. Instead, the relevance and prominence of identified methodological discussions will be described transparently, considering the content of the recommendation, the type of source, whether similar issues are discussed across multiple sources, and the potential implications for reporting reliability, and agreement/measurement error studies. To enhance trustworthiness, we will use a structured codebook, document coding decisions, discuss uncertainties and changes to the codebook within the multidisciplinary author team, and provide examples linking synthesized themes to the underlying source material.

The initial codebook will be structured around the following deductive codes, derived from our research objectives: measurement property addressed (e.g., reliability, agreement, measurement error); methodological issues discussed (e.g., study design recommendations, statistical methods, handling of measurement error (absolute/relative), risk of bias in reliability/agreement studies, reporting quality); whether existing frameworks are referenced (e.g., COSMIN, QAREL, GRRAS, STARD); and definitions (e.g., agreement, reliability). As coding progresses, inductive codes will be developed to capture ideas or concepts not anticipated in the initial framework. During synthesis, we

For peer review only

will document whether themes are supported by systematically identified sources or arise mainly from supplementary sources.

Results will be synthesized descriptively. Study characteristics will be presented in tabular and graphical formats and summarized narratively in the text. For review question 1, we will map which specific GRRAS items have been commented on, identify proposals for their revision and clarifications. For review question 2, we will narratively summarize the key methodological developments identified through our hybrid 'codebook' TA, focusing on aspects related to the design, conduct, reporting, and interpretation of reliability and agreement studies. These findings may be organized into overarching categories such as conceptual discussions, practical recommendations, and methodological advancements. Furthermore, we aim to identify generated themes not currently addressed by GRRAS that may inform future development of the GRRAS-COSMIN guideline.

## ETHICS AND DISSEMINATION

This study will involve publicly available data. No ethical approval is needed. Results will be disseminated through an open-access journal publication and national and international conference presentations. Data curation and the analysis will be documented and openly accessible from the Open Science Framework.

### Patient and public involvement

Patients and/or the public were not involved in this research's design, conduct, reporting or dissemination plans.

## DISCUSSION

Reliability and agreement/measurement error are fundamental measurement properties in healthcare, psychology, and education [8,23,47–51]. Transparent, accurate, and complete reporting of studies evaluating these properties is essential for the appropriate interpretation and application of measurement instruments. This methods review constitutes the first step in a structured revision of the GRRAS, informed by recent conceptual and methodological developments and conducted in accordance with established standards for reporting guideline development, as outlined by the EQUATOR Network [6]. The results will inform the development of an initial draft of the GRRAS-COSMIN guideline for reporting studies on reliability and agreement/measurement error, which will subsequently be refined through a modified Delphi consensus process involving key stakeholders and experts.

Well-developed reporting guidelines serve as key tools for enhancing both the reporting and design of research. They provide structured guidance on the minimum essential information to be included when reporting specific types of studies, thereby supporting completeness, clarity, and transparency [52]. Although their primary function is to assist authors during manuscript preparation [53], the influence of reporting guidelines goes beyond. When considered early, for instance during study protocol development, reporting guidelines can support improved study planning. When followed during reporting, these guidelines ensure that readers have sufficient information to assess the methodological quality of the research – in the case of GRRAS, the reliability and agreement/measurement error properties of a given measurement instrument.

Improved reporting also benefits systematic reviewers, developers of clinical practice guidelines, and those conducting electronic literature searches [54]. Complete and standardised reporting is crucial

For peer review only

for the conduct of meta-analyses and systematic reviews. Incomplete or inconsistent reporting poses significant barriers to the synthesis of findings across studies [52]. In the field of reliability, agreement and measurement studies this limits the ability to appraise the quality of measurement instruments and hampers the development of cumulative evidence bases. Complete and standardised reporting is thus critical not only for individual study interpretation and informed decision-making but also for the advancement of measurement science more broadly.

Importantly, reporting guidelines do not dictate how research should be conducted. Rather, by articulating what information must be transparently reported, they indirectly encourage better research practices and facilitate the assessment of adherence to contemporary methodological standards. For example, inadequate reporting in clinical trials may result in inflated confidence in the efficacy of an intervention. Similarly, insufficient detail in studies of reliability and agreement can lead to incorrect conclusions about measurement properties. In both cases, the downstream consequences include misguided clinical decision-making and reduced reproducibility. Over time, the widespread use of rigorously developed reporting guidelines, such as the GRRAS-COSMIN reporting guideline, has the potential to enhance research quality by fostering greater methodological awareness and transparency across disciplines.

**Limitations**

A comprehensive search strategy with no language restrictions will be employed across multiple databases to ensure thorough coverage of the literature. Nonetheless, this review has several methodological limitations that should be considered when interpreting the findings. First, the search strings are specific and focused, which may improve precision but could limit sensitivity and risk missing relevant literature. Specifically, this review seeks to identify methodological developments relevant to reliability, agreement, and measurement error in health, psychological or educational research and focuses on databases that primarily index health, psychological, and educational literature. Consequently, methodological developments published exclusively in, for example, mathematics or statistics journals may not be comprehensively captured.

Second, because the review includes diverse publication types such as methodological papers, tutorials, editorials, commentaries, guidance documents, and textbook material, and because the objective is to identify methodological developments rather than evaluate the validity of these studies, no formal critical appraisal of included sources will be undertaken. Consequently, the review will describe and synthesise methodological developments discussed in the literature but will not assess the relative merit or empirical support of competing methodological approaches.

Third, identifying and synthesising methodological developments requires a degree of interpretive judgement. This is inherent to qualitative synthesis [55]. We will address this through study selection being performed independently by two reviewers, documentation of coding decisions, reflexive discussions within the multidisciplinary author team, and transparent reporting of how themes are supported by the included sources.

Finally, while the absence of language restrictions may reduce the risk of language bias, non-English and non-German sources will be translated using automated translation tools. Consequently, inaccuracies in translation or subtle differences in terminology and meaning may affect the interpretation of methodological concepts, definitions, or recommendations. We will seek to minimise this risk by checking key passages against the original text where necessary.

For peer review only

## Conclusion

Taken together, this methods review will provide a critical foundation for updating GRRAS into GRRAS-COSMIN reporting guideline. By mapping current reporting practices in reliability and agreement/measurement error studies, it aims to support the development of updated reporting guidelines that foster transparency and reproducibility and synthesis these studies.

For peer review only

## References

1 Kottner J, Audigé L, Brorson S, *et al.* Guidelines for Reporting Reliability and Agreement Studies (GRRAS) were proposed. *J Clin Epidemiol*. 2011;64:96–106. doi: 10.1016/j.jclinepi.2010.03.002

2 Mokkink LB, Eekhout I, Boers M, *et al.* Studies on Reliability and Measurement Error of Measurements in Medicine - From Design to Statistics Explained for Medical Researchers. *Patient Relat Outcome Meas*. 2023;14:193–212. doi: 10.2147/PROM.S398886

3 COSMIN definitions of domains, measurement properties, and aspects of measurement properties. https://cosmin.nl/wp-content/uploads/COSMIN-definitions-domains-measurement-properties.pdf (accessed 19 August 2025)

4 American Educational Research Association, American Psychological Association, National Council on Measurement in Education. *Standards for educational and psychological testing*. Washington, DC: American Educational Research Association 2014.

5 Kottner J, Audige L, Brorson S, *et al.* Guidelines for Reporting Reliability and Agreement Studies (GRRAS) were proposed. *Int J Nurs Stud*. 2011;48:661–71. doi: 10.1016/j.ijnurstu.2011.01.016

6 What is a reporting guideline? | EQUATOR Network. https://www.equator-network.org/about-us/what-is-a-reporting-guideline/ (accessed 3 June 2025)

7 Kottner J, Streiner DL. The difference between reliability and agreement. *J Clin Epidemiol*. 2011;64:701–2. doi: 10.1016/j.jclinepi.2010.12.001

8 Streiner DL. Statistics Commentary Series: Commentary #15—Reliability. *J Clin Psychopharmacol*. 2016;36:305. doi: 10.1097/JCP.0000000000000517

9 Kottner J, Streiner DL. Internal consistency and Cronbach's *α*: A comment on Beeckman et al. (2010). *Int J Nurs Stud*. 2010;47:926–8. doi: 10.1016/j.ijnurstu.2009.12.018

10 Vach W, Gerke O. Gwet's AC1 is not a substitute for Cohen's kappa – A comparison of basic properties. *MethodsX*. 2023;10:102212. doi: 10.1016/j.mex.2023.102212

11 Van Tiggelen H, Kottner J, Campbell K, *et al.* Measurement properties of classifications for skin tears: A systematic review. *Int J Nurs Stud*. 2020;110:103694. doi: 10.1016/j.ijnurstu.2020.103694

12 Cairney J, Streiner DL. Using relative improvement over chance (RIOC) to examine agreement between tests: Three case examples using studies of developmental coordination disorder (DCD) in children. *Res Dev Disabil*. 2011;32:87–92. doi: 10.1016/j.ridd.2010.09.001

13 de Vet H, Mokkink L, Terwee C, *et al.* Clinicians are right not to like Cohen's κ. *BMJ-Br Med J*. 2013;346. doi: 10.1136/bmj.f2125

14 Bourredjem A, Cardot H, Devilliers H. Asymptotic Confidence Interval, Sample Size Formulas and Comparison Test for the Agreement Intra-Class Correlation Coefficient in Inter-Rater Reliability Studies. *Stat Med*. 2024;43:5060–76. doi: 10.1002/sim.10217

15 Lu M-J, Zhong W-H, Liu Y-X, *et al.* Sample Size for Assessing Agreement between Two Methods of Measurement by Bland–Altman Method. *Int J Biostat*. 2016;12. doi: 10.1515/ijb-2015-0039

For peer review only

16 Hong H, Choi Y, Hahn S, *et al.* Nomogram for sample size calculation on a straightforward basis for the kappa statistic. *Ann Epidemiol*. 2014;24:673–80. doi: 10.1016/j.annepidem.2014.06.097

17 Mokkink LB, de Vet H, Diemeer S, *et al.* Sample size recommendations for studies on reliability and measurement error: an online application based on simulation studies. *Health Serv Outcomes Res Methodol*. 2023;23:241–65. doi: 10.1007/s10742-022-00293-9

18 Oster C, Schoo A, Litt J, *et al.* Supporting workforce practice change: protocol for a pilot study of a motivational interviewing virtual client software tool for health professionals. *BMJ OPEN*. 2020;10. doi: 10.1136/bmjopen-2019-033080

19 Gerke O, Möller S, Debrabant B, *et al.* Experience Applying the Guidelines for Reporting Reliability and Agreement Studies (GRRAS) Indicated Five Questions Should Be Addressed in the Planning Phase from a Statistical Point of View. *Diagnostics*. 2018;8:69. doi: 10.3390/diagnostics8040069

20 Gagnier J, de Arruda G, Terwee C, *et al.* COSMIN reporting guideline for studies on measurement properties of patient-reported outcome measures: version 2.0. *Qual LIFE Res*. 2025;34:1901–11. doi: 10.1007/s11136-025-03950-x

21 Elsman EBM, Mokkink LB, Terwee CB, *et al.* Guideline for reporting systematic reviews of outcome measurement instruments (OMIs): PRISMA-COSMIN for OMIs 2024. *J PATIENT-Rep OUTCOMES*. 2024;8. doi: 10.1186/s41687-024-00727-7

22 Mokkink LB, Elsman EBM, Terwee CB. COSMIN guideline for systematic reviews of patient-reported outcome measures version 2.0. *Qual Life Res*. 2024;33:2929–39. doi: 10.1007/s11136-024-03761-6

23 Mokkink LB, Boers M, Vleuten CPM van der, *et al.* COSMIN Risk of Bias tool to assess the quality of studies on reliability or measurement error of outcome measurement instruments: a Delphi study. *BMC Med Res Methodol*. 2020;20. doi: 10.1186/s12874-020-01179-5

24 Lucas NP, Macaskill P, Irwig L, *et al.* The development of a quality appraisal tool for studies of diagnostic reliability (QAREL). *J Clin Epidemiol*. 2010;63:854–61. doi: 10.1016/j.jclinepi.2009.10.002

25 Cohen JF, Korevaar DA, Altman DG, *et al.* STARD 2015 guidelines for reporting diagnostic accuracy studies: explanation and elaboration. *BMJ Open*. 2016;6:e012799. doi: 10.1136/bmjopen-2016-012799

26 Bossuyt PM, Reitsma JB, Bruns DE, *et al.* STARD 2015: an updated list of essential items for reporting diagnostic accuracy studies. *BMJ*. 2015;351:h5527. doi: 10.1136/bmj.h5527

27 Whiting PF, Rutjes AWS, Westwood ME, *et al.* QUADAS-2: a revised tool for the quality assessment of diagnostic accuracy studies. *Ann Intern Med*. 2011;155:529–36. doi: 10.7326/0003-4819-155-8-201110180-00009

28 Whiting P, Rutjes AW, Reitsma JB, *et al.* The development of QUADAS: a tool for the quality assessment of studies of diagnostic accuracy included in systematic reviews. *BMC Med Res Methodol*. 2003;3:25. doi: 10.1186/1471-2288-3-25

29 Moher D, Schulz KF, Simera I, *et al.* Guidance for Developers of Health Research Reporting Guidelines. *PLOS Med*. 2010;7:e1000217. doi: 10.1371/journal.pmed.1000217

For peer review only

30 Peters MDJ, Godfrey C, McInerney P, *et al.* Best practice guidance and reporting items for the development of scoping review protocols. *JBI Evid Synth*. 2022;20:953. doi: 10.11124/JBIES-21-00242

31 Clarke M. Guide to the contents of a Cochrane Methodology protocol and review.

32 Aguinis H, Ramani RS, Alabduljader N. Best-Practice Recommendations for Producers, Evaluators, and Users of Methodological Literature Reviews. *Organ Res Methods*. 2023;26:46–76. doi: 10.1177/1094428120943281

33 Hirt J, Ewald H, Briel M, *et al.* Searching a methods topic: practical challenges and implications for search design. *J Clin Epidemiol*. 2024;166:111201. doi: 10.1016/j.jclinepi.2023.10.017

34 Gentles SJ, Charles C, Nicholas DB, *et al.* Reviewing the research methods literature: principles and strategies illustrated by a systematic overview of sampling in qualitative research. *Syst Rev*. 2016;5:172. doi: 10.1186/s13643-016-0343-0

35 Tricco AC, Lillie E, Zarin W, *et al.* PRISMA Extension for Scoping Reviews (PRISMA-ScR): Checklist and Explanation. *Ann Intern Med*. 2018;169:467–73. doi: 10.7326/M18-0850

36 KI-Plattform von DeepL: Übersetzung, Voice und API. https://www.deepl.com (accessed 1 June 2026)

37 Vet HCW de, Mokkink LB, Terwee CB, *et al.* Clinicians are right not to like Cohen's κ. *BMJ*. 2013;346:f2125. doi: 10.1136/bmj.f2125

38 Clark J, Glasziou P, Del Mar C, *et al.* A full systematic review was completed in 2 weeks using automation tools: a case study. *J Clin Epidemiol*. 2020;121:81–90. doi: 10.1016/j.jclinepi.2020.01.008

39 Forbes C, Greenwood H, Carter M, *et al.* Automation of duplicate record detection for systematic reviews: Deduplicator. *Syst Rev*. 2024;13:206. doi: 10.1186/s13643-024-02619-9

40 Identify trusted publishers for your research • Think. Check. Submit. Think Check Submit. https://thinkchecksubmit.org/ (accessed 5 June 2026)

41 Organisation for Economic Co-operation and Development. OECD schema to Web of Science Category Mapping 2022.

42 MAXQDA Software für qualitative Datenanalyse. 1989.

43 Hsieh H-F, Shannon SE. Three approaches to qualitative content analysis. *Qual Health Res*. 2005;15:1277–88. doi: 10.1177/1049732305276687

44 Graneheim UH, Lundman B. Qualitative content analysis in nursing research: concepts, procedures and measures to achieve trustworthiness. *Nurse Educ Today*. 2004;24:105–12. doi: 10.1016/j.nedt.2003.10.001

45 10.2.8 Analysis of the evidence - JBI Manual for Evidence Synthesis - JBI Global Wiki. https://jbi-global-wiki.refined.site/space/MANUAL/355862791/10.2.8+Analysis+of+the+evidence (accessed 3 July 2025)

For peer review only

46 Braun V, and Clarke V. One size fits all? What counts as quality practice in (reflexive) thematic analysis? *Qual Res Psychol*. 2021;18:328–52. doi: 10.1080/14780887.2020.1769238

47 De Vet HCW, Terwee CB, Mokkink LB, *et al. Measurement in Medicine: A Practical Guide*. 1st ed. Cambridge University Press 2011.

48 Streiner DL, Kottner J. Recommendations for reporting the results of studies of instrument and scale development and testing. *J Adv Nurs*. 2014;70:1970–9. doi: 10.1111/jan.12402

49 Streiner DL, Norman GR, Cairney J. *Health Measurement Scales: A practical guide to their development and use*. Sixth Edition, Sixth Edition. Oxford, New York: Oxford University Press 2024.

50 Prinsen CAC, Vohra S, Rose MR, *et al.* How to select outcome measurement instruments for outcomes included in a “Core Outcome Set” – a practical guideline. *Trials*. 2016;17:449. doi: 10.1186/s13063-016-1555-2

51 Kottner J., Blume-Peytavi U. Reliability and agreement of instrumental skin barrier measurements in clinical pressure ulcer prevention research. *Int Wound J*. 2021;18:716 EP – 727. doi: 10.1111/iwj.13574

52 Importance of Transparent Reporting of Health Research. *Guidelines for Reporting Health Research: A User’s Manual*. Wiley 2014:1–13.

53 Simera I, Altman DG. Writing a research article that is “fit for purpose”: EQUATOR Network and reporting guidelines. *Ann Intern Med*. 2009;151:JC2-2. doi: 10.7326/0003-4819-151-4-200908180-02002

54 Using Reporting Guidelines Effectively to Ensure Good Reporting of Health Research. *Guidelines for Reporting Health Research: A User’s Manual*. Wiley 2014:32–40.

55 Field SM, Pownall M. Subjectivity is a Feature, not a Flaw: A Call to Unsilence the Human Element in Science. 2025. doi: 10.31219/osf.io/ga5fb_v1

**Author contributions:** D.L.S.: Funding acquisition, Methodology, and Writing - review & editing. J.K.: Conceptualization, Funding acquisition, Methodology, Project administration, Supervision, and Writing - review & editing. L.B.M.: Funding acquisition, Methodology, and Writing - review & editing. M.-M.P.: Conceptualization, Data curation, Methodology, Project administration, Resources, and Writing - original draft. M.M.: Data curation, Methodology, and Writing - review & editing. M.S.: Funding acquisition, Methodology, and Writing - review & editing. S.B.: Funding acquisition, Methodology, and Writing - review & editing. M.-M.P. is responsible for the overall content as guarantor.

**Funding statement:** The project is funded by the Deutsche Forschungsgemeinschaft (DFG, German Research Foundation) – 560535232.

**Competing Interest Statement:** The authors have no conflict of interest to declare.

**Acknowledgement:** We thank Corinna Dressler, PhD; Medical Library Charité-Universitätsmedizin Berlin for her support in the development of the search strategy.

For peer review only

# Supplement of: Developments in the field of reliability and agreement studies in health, psychological, and educational measurement: a methods review protocol

Pittelkow, M.-M., Maximilian M., Mokkink, L.B., Streiner, D. L., Brorson S., Shoukri M., Kottner J.

This supplement details the sample references and search string development corresponding to review question 2 of the protocol "Developments in the field of reliability and agreement studies in health, psychological, and educational measurement: a methods review protocol
".

**Review question 2**: What major methodological discussions, recommendations, and/or advancements have been made in the last decade concerning the design, conduct, analysis, and reporting of reliability, and agreement/measurement error studies?

## Sample references

This is a preliminary list of key references to be included in the scoping review in addition to the sources identified via the systematic search. Please note that this list is not exhaustive and sources may be added during the conduct of the review.

**Journal articles**

1. Borg, D. N., Bach, A. J. E., O'Brien, J. L., & Sainani, K. L. (2022). Calculating sample size for reliability studies. PM & R : The Journal of Injury, Function, and Rehabilitation, 14(8), 1018–1025. https://doi.org/10.1002/pmrj.12850
2. Cohen JF, Korevaar DA, Altman DG, Bruns DE, Gatsonis CA, Hooft L, Irwig L, Levine D, Reitsma JB, de Vet HC, Bossuyt PM. STARD 2015 guidelines for reporting diagnostic accuracy studies: explanation and elaboration. BMJ Open. 2016 Nov 14;6(11):e012799. https://doi.org/10.1136/bmjopen-2016-012799.
3. Koo, T. K., & Li, M. Y. (2016). A Guideline of Selecting and Reporting Intraclass Correlation Coefficients for Reliability Research. Journal of Chiropractic Medicine, 15(2), 155–163. https://doi.org/10.1016/j.jcm.2016.02.012
4. Mokkink, L. B., Boers, M., van der Vleuten, C. P. M., Bouter, L. M., Alonso, J., Patrick, D. L., de Vet, H. C. W., & Terwee, C. B. (2020). COSMIN Risk of Bias tool to assess the quality of studies on reliability or measurement error of outcome measurement instruments: A Delphi study. BMC Medical Research Methodology, 20(1), 293. https://doi.org/10.1186/s12874-020-01179-5
5. Mokkink, L. B., de Vet, H., Diemeer, S., & Eekhout, I. (2023). Sample size recommendations for studies on reliability and measurement error: An online application based on simulation studies.

For peer review only

Health Services and Outcomes Research Methodology, 23(3), 241–265. https://doi.org/10.1007/s10742-022-00293-9

6. Mokkink, L. B., Eekhout, I., Boers, M., van der Vleuten, C. P. M., & de Vet, H. C. W. (2023). Studies on Reliability and Measurement Error of Measurements in Medicine – From Design to Statistics Explained for Medical Researchers. Patient Related Outcome Measures, 14, 193–212. https://doi.org/10.2147/PROM.S398886
7. Oster, N. V., Carney, P. A., Allison, K. H., Weaver, D. L., Reisch, L. M., Longton, G., Onega, T., Pepe, M., Geller, B. M., Nelson, H. D., Ross, T. R., Tosteson, A. N. A., & Elmore, J. G. (2013). Development of a diagnostic test set to assess agreement in breast pathology: Practical application of the Guidelines for Reporting Reliability and Agreement Studies (GRRAS). BMC Women's Health, 13(1), 3. https://doi.org/10.1186/1472-6874-13-3
8. Stratford, P., Spadoni, G. F., Kuspinar, A., & Macedo, L. (2025). Considerations when designing, analyzing, and reporting reliability studies. Brazilian Journal of Physical Therapy, 29(3), 101193. https://doi.org/10.1016/j.bjpt.2025.101193
9. Vach, W., & Gerke, O. (2023). Gwet's AC1 is not a substitute for Cohen's kappa – A comparison of basic properties. MethodsX, 10, 102212. https://doi.org/10.1016/j.mex.2023.102212
10. Vet, H. C. W. de, Mokkink, L. B., Terwee, C. B., Hoekstra, O. S., & Knol, D. L. (2013). Clinicians are right not to like Cohen's κ. BMJ, 346, f2125. https://doi.org/10.1136/bmj.f2125
11. Tran, D., Dolgun, A., & Demirhan, H. (2020). Weighted inter-rater agreement measures for ordinal outcomes. *Communications in Statistics - Simulation and Computation*, *49*(4), 989–1003. https://doi.org/10.1080/03610918.2018.1490428
12. ten Hove, D., Jorgensen, T. D., & van der Ark, L. A. (2024). Updated guidelines on selecting an intraclass correlation coefficient for interrater reliability, with applications to incomplete observational designs. Psychological Methods, 29(5), 967–979. https://doi.org/10.1037/met0000516
13.

**Textbooks**

14. De Vet, H. C., Terwee, C. B., Mokkink, L. B., & Knol, D. L. (2011). *Measurement in medicine: a practical guide*. Cambridge university press.
15. American Educational Research Association, American Psychological Association, & National Council on Measurement in Education. (2014). Standards for educational and psychological testing. American Educational Research Association.
16. Streiner, D. L., Norman, G. R., & Cairney, J. (2024). *Health Measurement Scales: A practical guide to their development and use* (Sixth Edition, Sixth Edition). Oxford University Press.

For peer review only

**Manual**

17. Mokkink, L. B., E. Elsman, C.B. Terwee. COSMIN guideline for systematic reviews of Patient-Reported Outcome Measures version 2.0. Qual Life Res (2024). https://doi.org/10.1007/s11136-024-03761-6.

For peer review only

## Search String Development

The search string was developed with both sensitivity (maximizing the identification of relevant articles) and feasibility (retrieving a manageable number of records for screening by two reviewers) in mind. Finding this balance can be especially challenging for reviews of methods topics [1]. We started by compiling a reference sample based on PubMed IDs of key journal articles (i.e., 28137831, 27330520, 37448975, 23379630, 27464308, 40022968, 37234937, 23585065, 33267819, 35596122). These articles serve as examples of the types of methodological literature we aim to identify and are not necessarily expected to be fully captured by the final search string. For example, we decided a priori to limit the search to publications from the last decade (1.1.2015 to 30.6.2025), since our aim is to cover recent methodological discussions and advancements. As a result, some of the articles included in the reference sample may not appear in the search output due to their earlier publication date.

In a first step, we identified relevant search terms and Medical Subject Headings (MeSH) terms. To this end, we (1) used the Systematic Review Accelerator (SR-Accelerator) [2] to extract and review frequently used words in the key references (see table S1) and (2) reviewed the assigned MeSH terms for the reference sample using the Yale Mesh Analyzer (1, see table S2). We additionally consulted the term finder in Ovid for MeSH terms related to reliability, agreement, or measurement error as well as the Thesaurus for Health Research Methods from the Library of Guidance for Health Scientists [4].

*Table S1. Word frequencies in title, abstract, and keywords of key references.*

| word | unique | title | abstract | keywords | points |
|---|---|---|---|---|---|
| reliability | 6 | 5 | 18 | 0 | 23 |
| agreement | 4 | 3 | 18 | 0 | 21 |
| measurement | 3 | 4 | 12 | 0 | 16 |
| study | 3 | 1 | 14 | 0 | 15 |
| icc | 2 | 0 | 14 | 0 | 14 |
| studies | 5 | 6 | 7 | 0 | 13 |
| between | 5 | 1 | 10 | 0 | 11 |
| cohen's | 2 | 1 | 9 | 0 | 10 |
| reporting | 4 | 4 | 6 | 0 | 10 |
| methods | 3 | 1 | 8 | 0 | 9 |
| kappa | 1 | 1 | 8 | 0 | 9 |
| ac1 | 1 | 1 | 8 | 0 | 9 |
| design | 4 | 0 | 9 | 0 | 9 |
| gwet's ac1 | 1 | 0 | 8 | 0 | 8 |
| gwet's | 1 | 0 | 8 | 0 | 8 |
| error | 2 | 2 | 6 | 0 | 8 |
| measure | 1 | 0 | 7 | 0 | 7 |
| outcome measurement | 1 | 1 | 6 | 0 | 7 |
| outcome | 1 | 1 | 6 | 0 | 7 |
| cohen's kappa | 1 | 0 | 7 | 0 | 7 |
| measurements | 2 | 0 | 7 | 0 | 7 |
| form | 1 | 0 | 6 | 0 | 6 |
| values | 4 | 0 | 6 | 0 | 6 |
| test | 4 | 2 | 4 | 0 | 6 |
| repeated | 2 | 0 | 6 | 0 | 6 |
| measurement error | 2 | 2 | 4 | 0 | 6 |
| size | 2 | 1 | 5 | 0 | 6 |
| **word** | **unique** | **title** | **abstract** | **keywords** | **points** |
| sample | 2 | 1 | 5 | 0 | 6 |
| when | 3 | 1 | 5 | 0 | 6 |
| κ | 1 | 1 | 4 | 0 | 5 |
| article | 1 | 0 | 5 | 0 | 5 |
| instrument | 1 | 0 | 5 | 0 | 5 |
| assess | 2 | 2 | 3 | 0 | 5 |
| rate | 1 | 0 | 5 | 0 | 5 |

For peer review only

| | | | | | |
|---|---|---|---|---|---|
| researchers | 3 | 0 | 5 | 0 | 5 |
| ratings | 3 | 0 | 5 | 0 | 5 |
| different | 4 | 0 | 5 | 0 | 5 |
| scores | 2 | 0 | 5 | 0 | 5 |
| correlation | 2 | 1 | 4 | 0 | 5 |
| e | 3 | 0 | 5 | 0 | 5 |
| based | 3 | 1 | 4 | 0 | 5 |
| raters | 4 | 0 | 5 | 0 | 5 |
| information | 4 | 1 | 4 | 0 | 5 |
| results | 3 | 0 | 5 | 0 | 5 |
| sample size | 2 | 1 | 4 | 0 | 5 |
| purpose | 1 | 0 | 5 | 0 | 5 |
| quantity | 1 | 0 | 4 | 0 | 4 |
| clinicians | 1 | 1 | 3 | 0 | 4 |
| diagnostic | 2 | 2 | 2 | 0 | 4 |
| research | 3 | 1 | 3 | 0 | 4 |
| reached | 1 | 0 | 4 | 0 | 4 |
| consensus | 1 | 0 | 4 | 0 | 4 |
| variation | 2 | 0 | 4 | 0 | 4 |
| how | 1 | 0 | 4 | 0 | 4 |

| word | unique | title | abstract | keywords | points |
|---|---|---|---|---|---|
| measurement instruments | 1 | 1 | 3 | 0 | 4 |
| instruments | 1 | 1 | 3 | 0 | 4 |
| quality | 1 | 1 | 3 | 0 | 4 |
| agreement rate | 1 | 0 | 4 | 0 | 4 |
| positive | 2 | 0 | 4 | 0 | 4 |
| properties | 2 | 1 | 3 | 0 | 4 |
| developed | 3 | 0 | 4 | 0 | 4 |
| k | 1 | 0 | 4 | 0 | 4 |
| repeated measurements | 1 | 0 | 4 | 0 | 4 |
| patients | 2 | 0 | 4 | 0 | 4 |
| application | 2 | 2 | 2 | 0 | 4 |
| calculation | 3 | 0 | 4 | 0 | 4 |
| discussion | 3 | 0 | 4 | 0 | 4 |
| provide | 3 | 0 | 4 | 0 | 4 |
| study purpose | 1 | 0 | 4 | 0 | 4 |

| | | | | | |
|---|---|---|---|---|---|
| however | 4 | 0 | 4 | 0 | 4 |
| observations | 1 | 1 | 2 | 0 | 3 |
| same | 2 | 0 | 3 | 0 | 3 |
| cohen's κ | 1 | 1 | 2 | 0 | 3 |
| readers | 1 | 0 | 3 | 0 | 3 |
| appropriate | 2 | 0 | 3 | 0 | 3 |
| icc form | 1 | 0 | 3 | 0 | 3 |
| guidelines | 2 | 2 | 1 | 0 | 3 |
| breast | 1 | 1 | 2 | 0 | 3 |
| diagnostic test | 2 | 2 | 1 | 0 | 3 |

| word | unique | title | abstract | keywords | points |
|---|---|---|---|---|---|
| reported | 3 | 0 | 3 | 0 | 3 |
| standards | 1 | 0 | 3 | 0 | 3 |
| measurement instrument | 1 | 0 | 3 | 0 | 3 |
| tool | 2 | 1 | 2 | 0 | 3 |
| both | 2 | 0 | 3 | 0 | 3 |
| paper | 3 | 0 | 3 | 0 | 3 |
| using | 3 | 0 | 3 | 0 | 3 |
| agreement between | 2 | 1 | 2 | 0 | 3 |
| basic | 2 | 1 | 2 | 0 | 3 |
| most | 2 | 0 | 3 | 0 | 3 |
| mse | 1 | 0 | 3 | 0 | 3 |
| parameters | 2 | 0 | 3 | 0 | 3 |
| about | 3 | 0 | 3 | 0 | 3 |
| intraclass correlation | 2 | 1 | 2 | 0 | 3 |
| intraclass | 2 | 1 | 2 | 0 | 3 |
| number | 2 | 0 | 3 | 0 | 3 |
| online application | 1 | 1 | 2 | 0 | 3 |
| online | 1 | 1 | 2 | 0 | 3 |
| whether | 2 | 0 | 3 | 0 | 3 |
| reliability study | 3 | 0 | 3 | 0 | 3 |
| reliability studies | 2 | 1 | 2 | 0 | 3 |
| reporting reliability | 2 | 2 | 1 | 0 | 3 |

For peer review only

Table S2. Results from the Yale MeSH Analyzer

| **PMID** | 28137831 | 27330520 | 37448975 | 23379630 | 27464308 | 40022968 | 37234937 | 23585065 | 33267819 |
|---|---|---|---|---|---|---|---|---|---|
| **MeSH Headings** | Advisory Committees* | | | | | | | | |
| | Bias<br>Biomedical Research | | | Breast / pathology<br>Breast Diseases / pathology*<br>Breast Neoplasms / classification*<br>Breast Neoplasms / diagnosis<br>Breast Neoplasms / pathology* | | | | | Bias |
| | Checklist* | | | | | | | | Consensus |
| | Diagnostic Techniques and Procedures / standards* | | | Diagnosis, Differential | Diagnostic Tests, Routine / standards* | | | | Delphi Technique* |
| | | | | Female | | | | | |
| | | | | | Guidelines as Topic | | | | |
| | Humans | | | Humans | Humans | Humans | | Humans | Humans |
| | | | | Observer Variation | | | | Observer Variation* | |

(continued)

For peer review only

Table S2 continued. Results from the Yale MeSH Analyzer

| **PMID** | 28137831 | 27330520 | 37448975 | 23379630 | 27464308 | 40022968 | 37234937 | 23585065 | 33267819 |
|---|---|---|---|---|---|---|---|---|---|
| | Publishing | | | Predictive Value of Tests | Psychology / standards<br>Psychometrics / standards*<br>Publishing / standards* | | | Probability* | |
| | | | | Quality Assurance, Health Care / standards* | | | | | |
| | Research Design | | | Reproducibility of Results<br>Research Design / standards | | Reproducibility of Results<br>Research Design* | | Reproducibility of Results*<br>Research Design* | Reproducibility of Results |
| | | | | Sensitivity and Specificity | Societies, Scientific | Sample Size | | | |
| | Terminology as Topic | | | | | | | | |
| **Author Assigned Keywords** | Diagnostic accuracy<br>Medical publishing<br>Peer review<br>Reporting quality<br>Research waste<br>Sensitivity and specificity | Reliability and validity<br>Research<br>Statistics | classical test theory<br>generalizability theory<br>intraclass correlation coefficient<br>measurement error<br>reliability<br>standard error of measurement | | | Measurement Methods<br>Rehabilitation<br>Reliability<br>Research design<br>Statistics | Agreement<br>Gwet's AC1<br>Method comparison<br>Observer<br>Rater<br>Reliability<br>Repeatability<br>Reproducibility | | COSMIN<br>Delphi study<br>Measurement error<br>Outcome measurement instruments<br>Quality assessment<br>Reliability<br>Risk of Bias |

For peer review only - http://bmjopen.bmj.com/site/about/guidelines.xhtml

From this, we constructed a set of possible search and MeSH terms (see table S3).

**Table S3. Potential search and MeSH terms. MeSH terms are retrieved from Medline via Ovid**

| Search concept | Potential search terms | |
|---|---|---|
| **Target:** | Reliability | Method* |
| | reproducibility of measurements | Outcome |
| **Methodological studies, reviews, commentaries, editorials, and guidance documents that explicitly discuss, evaluate, reflect on, or propose methodological aspects of reliability, agreement, and/or measurement error.** | Agreement | Cohen's |
| | Bland Altmann analysis | Kappa |
| | Measurement | ICC |
| | Measurement error | AC1 |
| | Measure* | gwet's |
| | Stud* | Guidance |
| | Reporting | How to |
| | Reporting quality | Best practice |
| | MeSH: Research Design / standards | MeSH: Statistics as Topic |
| | MeSH: Reproducibility of Results | MeSH: Reproducibility of Results |
| | MeSH: Publishing / standards* | MeSH: Methods / Research Design |

We then iteratively piloted the search terms in Medline via OVID to develop a combination that was both sensitive and resulted in a feasible number of hits. To check the sensitivity of our search, we searched for the reference sample in the hits of the search string before adding the publication date restriction.

## References

1 Hirt J, Ewald H, Briel M, *et al.* Searching a methods topic: practical challenges and implications for search design. *J Clin Epidemiol*. 2024;166:111201. doi: 10.1016/j.jclinepi.2023.10.017

2 Clark J, Glasziou P, Del Mar C, *et al.* A full systematic review was completed in 2 weeks using automation tools: a case study. *J Clin Epidemiol*. 2020;121:81–90. doi: 10.1016/j.jclinepi.2020.01.008

3 Yale MeSH Analyzer. https://mesh.med.yale.edu/ (accessed 23 July 2025)

4 Hirt J, Schönenberger CM, Ewald H, *et al.* Introducing the Library of Guidance for Health Scientists (LIGHTS): A Living Database for Methods Guidance. *JAMA Netw Open*. 2023;6:e2253198. doi: 10.1001/jamanetworkopen.2022.53198

For peer review only

BMJ Open

# Developments in the field of reliability and agreement studies in health, psychological, and educational measurement: a methods review protocol


| | |
|---|---|
| Journal: | *BMJ Open* |
| Manuscript ID | bmjopen-2025-109876.R2 |
| Article Type: | Protocol |
| Date Submitted by the Author: | 01-Jul-2026 |



| | |
|---|---|
| Complete List of Authors: | Pittelkow, M.-M.; Charité – University Medical Center Berlin, corporate member of Freie Universität Berlin and Humboldt-Universität zu Berlin, Institute of Clinical Nursing Science; Berlin Institute of Health at Charité, Universitätsmedizin Berlin, QUEST Center for Responsible Research<br>Mönch, M; Charité - Universitätsmedizin Berlin, corporate member of Freie Universität Berlin and Humboldt-Universität zu Berlin, Institute of Biometry and Clinical Epidemiology<br>Mokkink, L.B.; Amsterdam UMC, Vrije Universiteit Amsterdam, Department of Epidemiology and Data Science; Amsterdam Public Health Research Institute, Methodology<br>Streiner, D.; McMaster University, Department of Psychiatry and Behavioural Neurosciences<br>Brorson, S.; University of Copenhagen, Centre for Evidence-Based Orthopaedics, Zealand University Hospital and Department of Clinical Medicine<br>Shoukri, M.; King Faisal Specialist Hospital and Research Centre, Biostatistics and Scientific Computing Department; University of Western Ontario, Department of Epidemiology and Biostatistics, Schulich School of Medicine and Dentistry<br>Kottner, J.; Charité – University Medical Center Berlin, corporate member of Freie Universität Berlin and Humboldt-Universität zu Berlin, Institute of Clinical Nursing Science |



| | |
|---|---|
| <b>Primary Subject Heading</b>: | Research methods |
| Secondary Subject Heading: | Diagnostics |
| Keywords: | STATISTICS & RESEARCH METHODS, Methods, Patient Reported Outcome Measures, Psychometrics, Systematic Review |
| | |


SCHOLARONE MANUSCRIPTS

# Developments in the field of reliability and agreement studies in health, psychological, and educational measurement: a methods review protocol

Pittelkow, M.-M.[1,2*], Mönch, M.[3], Mokkink, L.B. [4,5], Streiner, D. L.[6], Brorson S.[7], Shoukri M.[8,9], Kottner J.[1]

[1] Charité – University Medical Center Berlin, corporate member of Freie Universität Berlin and Humboldt-Universität zu Berlin, Institute of Clinical Nursing Science, Berlin, Germany

[2] Berlin Institute of Health at Charité – Universitätsmedizin Berlin, QUEST Center for Responsible Research, Berlin, Germany

[3] Charité – Universitätsmedizin Berlin, corporate member of Freie Universität Berlin and Humboldt-Universität zu Berlin, Institute of Biometry and Clinical Epidemiology, Charitéplatz 1, 10117 Berlin, Germany

[4] Amsterdam UMC, Vrije Universiteit Amsterdam, Department of Epidemiology and Data Science, Amsterdam, Netherlands

[5] Amsterdam Public Health research institute, Methodology, Amsterdam, Netherlands

[6] Department of Psychiatry and Behavioural Neurosciences, McMaster University, Hamilton, Ontario, Canada

[7] Centre for Evidence-Based Orthopaedics, Zealand University Hospital and Department of Clinical Medicine, University of Copenhagen, Denmark

[8] Biostatistics and Scientific Computing Department, King Faisal Specialist Hospital and Research Centre, Riyadh, 11211, Saudi Arabia

[9] Department of Epidemiology and Biostatistics, Schulich School of Medicine and Dentistry, University of Western Ontario, London, Ontario, Canada

[*] Correspondence should be addressed to Merle-Marie Pittelkow; Charité-Universitätsmedizin Berlin, Institute of Clinical Nursing Science, Charitéplatz 1, 10117 Berlin, Germany, Email: merle-marie.pittelkow@charite.de

For peer review only

## ABSTRACT

**Introduction**: The Guidelines for Reporting Reliability and Agreement Studies (GRRAS) were developed to improve the completeness and transparency of reporting of reliability and agreement of health measurement instruments. However, since their publication in 2011 methodological standards, both for reporting guideline development and reliability, agreement, and measurement error studies, have advanced, highlighting the need for aligning and updating. In addition, related initiatives like COnsensus-based Standards for the selection of health Measurement Instruments (COSMIN) have emerged, offering opportunities to harmonize terminology and promote consistency across diverse types of measurement instruments. This methods review aims (1) to systematically identify and synthesize commentaries on and evaluations of the original GRRAS, and (2) to map recent methodological developments in the planning, conduct, and interpretation of reliability, agreement and measurement error studies in health science, psychology and education that should be reflected in the reporting, to inform the development of the GRRAS-COSMIN reporting guidelines.

**Methods and analysis**: Two complementary search strategies will be employed. First, forward direct citation tracking of the original GRRAS publications will be conducted in Web of Science. We will include sources providing critique, commentaries, or suggestions related to the GRRAS. We will exclude publications that used GRRAS just for structuring their report, withdrawn or retracted articles, textbooks, peer reviews, supplements, and conference materials without full-text publications. Included articles will be analysed using descriptive content analysis. Second, we will perform a systematic search in Medline, PsycINFO, Embase, Eric, and Cinahl supplemented by key references identified by or known to the author team. We will include studies, reviews, commentaries, editorials, methodological papers, tutorials and guidance documents that discuss or critically reflect on methodological aspects of the measurement properties reliability and/or agreement/measurement error published between 01.01.2015 and 30.06.2026 in health sciences, psychology, or education. Eligibility will be assessed by two independent reviewers and included sources will be analysed using 'codebook' thematic analysis. Findings will be presented narratively supplemented by visuals and tables if appropriate.

**Ethics and dissemination**: This study involves publicly available data. No ethical approval is needed. Results will be disseminated through an open-access journal publication following the Preferred Reporting Items for Systematic Reviews and Meta-Analyses-Scoping Review Extension and conference presentations.


***Keywords:*** reliability, agreement, measurement error, reporting guidelines, methods review

## STRENGTH AND LIMITATIONS:

- This methods review protocol adheres to the methodological guidance provided by the Joanna Briggs Institute for conducting scoping reviews.
- Two complementary search strategies, each aligned with a specific review aim, are used, followed by tailored data synthesis for each.
- No language restrictions will be applied.
- A comprehensive search strategy is employed across multiple databases to ensure thorough coverage of the literature; however, the search strings are specific and focused, which may

For peer review only

improve precision but could limit sensitivity, especially for methodological literature that is inconsistently indexed or published outside health, psychology, and education databases.

- Grey literature will not be searched systematically; however, database searches will be supplemented by transparently documented additional sources.

For peer review only

## INTRODUCTION

In clinical, psychological or educational practice and research, obtained data (e.g. blood pressure, values of standardised pain scales, personality tests, evaluations of exams) or diagnoses (e.g. risk of falling, depression, melanoma yes/no) must be reliable. Reliability and agreement/measurement error are fundamental properties of health measurement instruments, including patient-reported outcome measures (PROMs), performance-based instruments, diagnostic test results, clinician-reported outcomes, or lab results. While reliability and agreement are often used interchangeably, they reflect conceptually distinct constructs. Definitions vary slightly (see Table 1), and for the purposes of this review, we adopt the following working definitions: *Reliability* refers to the ability of a measurement instrument to distinguish between individuals or objects [1,2]. It is operationalised as the ratio of variability between subjects (e.g., participants) to the total variability of all measurements in the sample. *Agreement*, also referred to as measurement error, is defined as the degree to which scores or ratings of a test or instrument are identical [1,2].

**Table 1. Selected examples of definitions of reliability and agreement as reported in the literature**

| | Reliability | Agreement/ Measurement error/ Error of measurement |
|---|---|---|
| COnsensus-based Standards for the selection of health Measurement Instruments (COSMIN) [3] | "the proportion of the total variance in the measurements which is due to the true difference between patients" (p. 1) [3] | "The systematic and random error of a patient's score that is not attributed to the true changes in the construct to be measured." (p.1) [3] |
| Guidelines for Reporting Reliability and Agreement Studies (GRRAS)[1] | "the ratio of variability between subjects (e.g., patients) or objects (e.g., computed tomography scans) to the total variability of all measurements in the sample" (p. 1) [1]<br>"the ability of a measurement to differentiate between subjects or objects" (p.1) [1] | "the degree to which scores or ratings are identical" (p.1) [1] |
| Standards for Educational and Psychological Testing [4] | "The degree to which test scores for a group of test takers are consistent over repeated applications of a measurement procedure and hence are inferred to be dependable and consistent for an individual test taker; the degree to which scores are free of random errors of measurement for a given group". [4] | "The difference between an observed score and the corresponding true score". [4] |

Since reliability and agreement/measurement error are essential for judging the quality of measurement instruments, and by extension directly impact the quality of research and practice, their transparent reporting is crucial. Only what is transparently reported can be critically assessed, interpreted, and built upon. To improve the quality, transparency, and consistency of reporting reliability, agreement and measurement error studies, a multidisciplinary working group developed the Guidelines for Reporting Reliability and Agreement Studies (GRRAS), published in 2011 [1,5]. Reporting guidelines - defined as "a checklist, flow diagram, or structured text to guide authors in

For peer review only

reporting a specific type of research, developed using explicit methodology" [6] - are recognised as valuable for enhancing the transparency and completeness of study reporting across various research designs. By articulating key elements to be addressed, such guidelines enhance transparency, support interpretability, and facilitate replicability and synthesis. Since its publication, GRRAS has been cited extensively and remains a frequently used reference for reporting reliability and agreement studies.

Nevertheless, several developments and ongoing challenges highlight the need to revisit and update the existing GRRAS. Central to these are enduring conceptual ambiguities, for example, the distinction between reliability and agreement/measurement error [7,8] and the appropriate use of internal consistency measures [9]. These uncertainties have contributed to methodological debates concerning the selection and interpretation of statistical coefficients [9,10] and errors in the application and reporting of method and results. This, in turn, hampers efforts to synthesize evidence and evaluate measurement quality in systematic reviews [1,11]. At the same time, important methodological discussions have advanced the assessment and interpretation of reliability, agreement, and measurement error in health, psychological, and educational research. These include discussions on agreement coefficients [10,12,13], recommendations for sample size determination for intraclass correlation coefficients (ICCs), κ statistics, and Bland–Altman approaches [14–17], guidance on the design, analysis, and interpretation of reliability and measurement error studies [2]. Commentaries on GRRAS have further emphasized the need for revision, offering constructive feedback and concrete suggestions for improvement [18,19].

In parallel, related initiatives have emerged, offering opportunities for conceptual and terminological alignment. Notably, the COnsensus-based Standards for the selection of health Measurement INstruments (COSMIN) developed several guidelines for conducting and reporting studies on measurement properties of PROMs [20] and for conducting and reporting systematic reviews on outcome measurement instruments [21,22], including a tool to assess the risk of bias in studies of reliability and agreement/measurement error [23]. While COSMIN initially focused risk of bias assessments of existing studies on any measurement property, and GRRAS targets specifically on reporting of studies on reliability and agreement, both frameworks address shared constructs. Harmonizing these efforts holds potential to improve clarity, promote consistency in terminology, and enhance the usability and uptake of reporting standards. Similarly, the Quality Appraisal tool for studies of Diagnostic Reliability (QAREL), developed by Lucas et al. [24], provides a structured framework for evaluating methodological quality in diagnostic reliability studies and is broadly applicable to reliability and agreement research.

In the related but different field of diagnostic accuracy, the Standards for Reporting of Diagnostic Accuracy Studies (STARD) statement [25,26] offers a comprehensive set of recommendations to ensure transparent and complete reporting, while the Quality Assessment of Diagnostic Accuracy Studies (QUADAS) tool [27,28] provides a structured approach for evaluating risk of bias of diagnostic accuracy studies included in systematic reviews.

Reporting and risk of bias assessment are inherently interdependent: only what is reported can be critically appraised. Therefore, reporting guidelines must explicitly include the elements targeted by risk of bias tools to ensure meaningful and comprehensive evaluation. In response to these developments and ongoing challenges, our multidisciplinary research group set out to develop the GRRAS-COSMIN guidelines, an updated and extended version of the original GRRAS.

**Objective**

For peer review only

For peer review only - http://bmjopen.bmj.com/site/about/guidelines.xhtml

The objective of this methods review is to systematically identify and summarize published comments, evaluations, and methodological discussions relevant to the development of the new GRRAS-COSMIN reporting guidelines for studies on reliability, agreement, and measurement error. While reporting guidelines do not prescribe how studies should be designed, conducted, or analysed, they should reflect contemporary methodological standards by ensuring that information relevant to assessing these aspects is reported transparently. Consequently, methodological developments may have implications not only for research practice itself, but also for what information should be reported to enable readers to judge whether current methodological recommendations have been followed. This process aligns with the methodological standards for reporting guideline development and revisions established by the EQUATOR Network [29].

Specifically, this review will examine literature published in the last decade that either explicitly critiques or evaluates the existing GRRAS, or that provides methodological recommendations, advancements, or reflections to inform best practice reporting of reliability, agreement and measurement error studies in health sciences, psychology, education. This includes issues related to the planning, conduct, analysis, and interpretation of such studies.

Specific review questions are:

1. What comments and/or evaluations have been published about GRRAS?
2. What major methodological discussions, recommendations, and/or advancements have been made in the last decade concerning the design, conduct, analysis, and reporting of reliability, and agreement/measurement error studies?

## METHODS

This protocol of a methods review is informed by the best practice guidelines for the development of scoping review protocols developed by the Joanna Briggs Institute [30] as well as relevant guidance for conducting methods reviews [31–34]. Results reporting will follow an adapted version of the Preferred Reporting Items for Systematic Reviews and Meta-Analyses-Scoping Review Extension [35]. To address the two review aims, we will implement two complementary search strategies.

### Eligibility criteria

To address review question 1, we will include peer-reviewed articles, commentaries, or other scholarly publications that cite one or both of the original GRRAS publications [1,5] and provide critique, commentaries, or suggestions related to the GRRAS guideline. We will exclude articles that cite GRRAS solely to structure their report; withdrawn or retracted articles; textbooks; peer-reviews; supplements; and conference abstracts, posters, or slide presentations without accompanying full-text publications. No language restrictions will be applied. Full texts published in languages other than English or German will be translated using DeepL Translator Pro [1][37]. Data extraction will be performed based on the translated text. In addition, where substantial contributions to the findings are derived from non-English or non-German sources, a back-translation procedure will be used for key excerpts to identify potential discrepancies in meaning.

In- and exclusion criteria to address review question 2 following the Population, Concept, and Context (PCC) scheme are displayed in Table 2.

[1] According to DeepL's Privacy Policy, submitted texts are only processed temporarily to generate the translation, deleted after completion of the service, and not used to improve DeepL's services [36].

For peer review only - http://bmjopen.bmj.com/site/about/guidelines.xhtml

| Table 2. In- and exclusion criteria for review question 2; structured following recommendations from [31]. | | |
|---|---|---|
| | **Inclusion criteria** | **Exclusion criteria** |
| **Types of documents** | • Studies, reviews, commentaries, editorials, methodological papers, dissertations, and guidance documents that explicitly discuss, evaluate, reflect on, or propose methodological aspects of the measurement properties reliability and/or agreement and/or measurement error<br>• Relevant textbook chapters.<br>Published between 01.01.2015 and 30.06.2026. | • Empirical studies investigating measurement properties without critical methodological reflection.<br>• Reviews summarizing measurement properties without methodological discussion.<br>• Conference abstracts, posters, slide presentations without full text.<br>• Errata, retracted or withdrawn articles. |
| **Types of information** | • Narrative discussions, recommendations, and proposals related to study design, planning, conduct, analysis, reporting, and interpretation of reliability/agreement studies.<br>• Text on measurement error (absolute and relative), reliability/agreement statistics (e.g., ICC, kappa, generalizability studies), and risk of bias appraisal. | • Purely empirical numeric results on measurement properties without methodological reflection<br>• Data on internal consistency/homogeneity.<br>• Data on reliability of automated systems. |
| **Types of methods** | • Methods and approaches for designing, conducting, analysing, and reporting reliability, agreement, and measurement error studies.<br>• Methods for appraising risk of bias in such studies. | • Methods addressing automated systems or engineering applications only. |
| **Types of disciplines** | • Health research, psychology and education | • Engineering; economics |

For peer review only

**Information sources**

To address review question 1, we will search Web of Science (WoS), which offers a broad and well-curated citation index across disciplines.

To address review question 2, we will consecutively search the databases Embase and Medline via OVID and PsycINFO, Eric, and Cinahl via EBSCOhost. These databases were selected based on two considerations: first, a search in the Information Matrix for the Analysis of Journals (MIAR; https://miar.ub.edu) to identify databases indexing the journals of key references; and second, their relevance for covering the fields we aim to include in this review. In addition, we will search the Charité Library (via Ex Libris Primo) for relevant books and book chapters.

**Search strategy**

To address review question 1, we will conduct direct forward citation tracking of the two original GRRAS guideline publications [1,5]. To this end, we will search WoS for all articles citing the original publications. A pilot search on July 1st 2025 resulted in 1557 citations for reference [1], and 441 citations for reference [5] respectively.

To address review question 2, the search string shown in Table 3 will be used with publication year restricted between 1.1.2015 to 30.6.2026. A detailed account of the development of the search string is documented in the supplement (Table S1-S3). The search string was developed in Medline via Ovid and will be translated to the other databases. The review will include supplementary sources identified by the author team. These sources are intended to supplement the systematic search by providing conceptual context, identifying seminal methodological contributions, and reducing the risk of overlooking influential work that may not be retrieved through database searching alone. This may include articles about methodological discussions [10,38]and relevant textbook chapters judged as important by the author team. Because these sources are intended to supplement the systematic database search, they are not necessarily subject to the same publication date restrictions. Additional sources will be included only if they are directly relevant to the review questions, and the source's identification and reason for inclusion will be documented. A list of additional sources is presented in the supplement. This list is not exhaustive, and additional sources will be added during the search. Lastly, reference lists of all included articles will be screened for additional relevant sources using the SpiderCite tool from the Systematic Review (SR) Accelerator [39].

**Table3.** Search string to address review question 2.

| Date | Database | Search String | Hits |
|---|---|---|---|
| 2026/0/02 | Medline via Ovid | Ovid MEDLINE(R) ALL <1946 to June 01, 2026><br>1 reliability.kf,ti. 62933<br>2 agreement.kf,ti. 15474<br>3 measurement error.kf,ti. 2208<br>4 reproducibility of measurements.kf,ti. 104<br>5 interrater.kf,ti. 1751<br>6 intrarater.kf,ti. 407<br>7 intraobserver.kf,ti. 982<br>8 5 or 6 or 7 2900<br>9 Cohen's.kf,ti. 297<br>10 kappa.kf,ti. 20011<br>11 ICC.kf,ti. 1126<br>12 Gwet's.kf,ti. 18<br>13 guidance.kf,ti. 31327<br>14 guideline.kf,ti. 32524<br>15 tutorial.kf,ti. 2611<br>16 checklist.kf,ti. 9827<br>17 exp "Statistics as Topic"/ 3347331<br>18 exp "Reproducibility of Results"/ 514177 | 2301 |

For peer review only - http://bmjopen.bmj.com/site/about/guidelines.xhtml

| | | | |
|---|---|---|---|
| | | 19 Research Design/ 135596 | |
| | | 20 Publishing/ or "Access to Information"/39696 | |
| | | 21 1 and 2 1109 | |
| | | 22 3 or 4 or 8 or 9 or 10 or 11 or 12 26450 | |
| | | 23 21 or 22 27336 | |
| | | 24 13 or 14 or 15 or 16 75846 | |
| | | 25 17 or 18 or 19 or 20 3776731 | |
| | | 26 24 or 25 3843007 | |
| | | 27 23 and 26 4982 | |
| | | 28 limit 22 to yr="2015 - 2026" 11698 | |
| | | 29 27 and 28 2301 | |

For peer review only

**Data management and selection process**

The retrieved records will be exported from the respective databases in XML format. For deduplication and screening and dispute we will use the tools provided by the SR Accelerator [39,40], a web-based platform designed to support systematic review workflows, per review question. First, duplicate entries will be identified and removed prior to screening. Two reviewers (MMP and MM) will then independently assess titles and abstracts. Full texts of potentially relevant studies will be retrieved and independently assessed against the eligibility criteria. If no full text can be extracted using standard methods (e.g., journal websites), we will (1) contact the library or (2) contact the corresponding author. Corresponding authors will be contacted twice with a follow-up after two weeks of no reply following the first contact. Reasons for exclusion during full text screening will be documented. Any disagreements will be resolved through discussions, with a third reviewer (JK) consulted in cases where consensus cannot be reached.

During full-text screening, we will also consider whether sources are published in journals or by publishers that raise concerns regarding editorial and publishing practices. For this purpose, we will use the Think. Check. Submit. checklist as guidance [41]. Sources for which substantial concerns arise will be discussed within the review team before a final inclusion decision is made. Reasons for exclusion will be documented.

**Data collection process and data items**

The first author (MMP) will extract the following, descriptive information from all included full text: title, author, publication year, country of the corresponding author, publication type, language, and field or discipline, and research question(s) or topic as stated by the authors (if available). The field or discipline of each source will be classified using the OECD (Organisation for Economic Co-operation and Development) Fields of Science categories [42]. For sources indexed in Web of Science, Web of Science subject categories will be extracted and mapped to OECD categories using the InCites OECD Category Schema. For sources not indexed in Web of Science, such as textbooks, guidance documents, or other supplementary sources, classification will be based on the source content, journal or book scope, and, where informative, author departments or affiliations. Multiple fields may be assigned where appropriate, and unclear cases will be discussed within the review team. Contextual information will be generated during data synthesis described below.

**Risk of bias in individual studies**

A critical appraisal of the evidence quality will not be conducted, since the included publication types are expected to be mostly opinion pieces which do not lend themselves to systematic quality assessment geared towards empirical studies [30].

**Data synthesis**

All included full-text publications will be uploaded to MAXQDA 24 [43] and coded by the first author (MMP). Different qualitative analytic approaches will be employed to address each research question.

To answer review question 1, we will employ qualitative content analysis using a directed approach [44]. The analysis is planned as deductive and descriptive, focusing on manifest content (i.e., what the text says instead of underlying meaning of the text [45]. Unit of analysis will be the individual sources. This approach is well-suited to systematically categorise and summarise clearly defined information, and thus is appropriate given our primary focus on descriptive and structured content, specifically how the GRRAS guidelines have been discussed or critiqued in the literature. Analysis will occur at the semantic level, emphasizing explicit statements, which will be organized into descriptive categories and summarized narratively. Prespecified codes include the type of measurement property addressed, definitions of reliability and agreement (if provided), the specific GRRAS items discussed, recommendations for revision such as proposed new items or clarifications, and commentary on the strengths and weaknesses of the original GRRAS.

To answer review question 2, we will use hybrid 'codebook' thematic analysis (TA) combining both deductive and inductive coding. We will use 'codebook' TA, defined by Braun and Clarke as a cluster of methods that use structured coding frameworks (e.g., codebooks), where themes are typically identified early but may be refined through further analysis [47]. This approach allows to begin with a set of a priori categories, while remaining open to inductively derived insights and aligns with our aim to describe the *main* developments, recurrent themes, and topics in the field. In some cases, extracting relevant content will be relatively straightforward—for example, documenting key concepts from included tutorials that illustrate standard approaches. In other cases, the process will be more interpretative, for example when grouping related methodological discussions into overarching categories and considering their relevance for reporting guidance. We will not apply an a priori threshold for what constitutes a "major" methodological development. Instead, the relevance and prominence of identified methodological discussions will be described transparently, considering the content of the recommendation, the type of source, whether similar issues are discussed across multiple sources, and the potential implications for reporting reliability, and agreement/measurement error studies. To enhance trustworthiness, we will use a structured codebook, document coding decisions, discuss uncertainties and changes to the codebook within the multidisciplinary author team, and provide examples linking synthesized themes to the underlying source material.

The initial codebook will be structured around the following deductive codes, derived from our research objectives: measurement property addressed (e.g., reliability, agreement, measurement error); methodological issues discussed (e.g., study design recommendations, statistical methods, handling of measurement error (absolute/relative), risk of bias in reliability/agreement studies, reporting quality); whether existing frameworks are referenced (e.g., COSMIN, QAREL, GRRAS, STARD); and definitions (e.g., agreement, reliability). As coding progresses, inductive codes will be developed to capture ideas or concepts not anticipated in the initial framework. During synthesis, we

For peer review only

will document whether themes are supported by systematically identified sources or arise mainly from supplementary sources.

Results will be synthesized descriptively. Study characteristics will be presented in tabular and graphical formats and summarized narratively in the text. For review question 1, we will map which specific GRRAS items have been commented on, identify proposals for their revision and clarifications. For review question 2, we will narratively summarize the key methodological developments identified through our hybrid ‘codebook’ TA, focusing on aspects related to the design, conduct, reporting, and interpretation of reliability and agreement studies. These findings may be organized into overarching categories such as conceptual discussions, practical recommendations, and methodological advancements. Furthermore, we aim to identify generated themes not currently addressed by GRRAS that may inform future development of the GRRAS-COSMIN guideline.

## ETHICS AND DISSEMINATION

This study will involve publicly available data. No ethical approval is needed. Results will be disseminated through an open-access journal publication and national and international conference presentations. Data curation and the analysis will be documented and openly accessible from the Open Science Framework.

### Patient and public involvement

Patients and/or the public were not involved in this research’s design, conduct, reporting or dissemination plans.

## DISCUSSION

Reliability and agreement/measurement error are fundamental measurement properties in healthcare, psychology, and education [8,23,48–52]. Transparent, accurate, and complete reporting of studies evaluating these properties is essential for the appropriate interpretation and application of measurement instruments. This methods review constitutes the first step in a structured revision of the GRRAS, informed by recent conceptual and methodological developments and conducted in accordance with established standards for reporting guideline development, as outlined by the EQUATOR Network [6]. The results will inform the development of an initial draft of the GRRAS-COSMIN guideline for reporting studies on reliability and agreement/measurement error, which will subsequently be refined through a modified Delphi consensus process involving key stakeholders and experts.

Well-developed reporting guidelines serve as key tools for enhancing both the reporting and design of research. They provide structured guidance on the minimum essential information to be included when reporting specific types of studies, thereby supporting completeness, clarity, and transparency [53]. Although their primary function is to assist authors during manuscript preparation [54], the influence of reporting guidelines goes beyond. When considered early, for instance during study protocol development, reporting guidelines can support improved study planning. When followed during reporting, these guidelines ensure that readers have sufficient information to assess the methodological quality of the research – in the case of GRRAS, the reliability and agreement/measurement error properties of a given measurement instrument.

Improved reporting also benefits systematic reviewers, developers of clinical practice guidelines, and those conducting electronic literature searches [55]. Complete and standardised reporting is crucial

For peer review only

for the conduct of meta-analyses and systematic reviews. Incomplete or inconsistent reporting poses significant barriers to the synthesis of findings across studies [53]. In the field of reliability, agreement and measurement studies this limits the ability to appraise the quality of measurement instruments and hampers the development of cumulative evidence bases. Complete and standardised reporting is thus critical not only for individual study interpretation and informed decision-making but also for the advancement of measurement science more broadly.

Importantly, reporting guidelines do not dictate how research should be conducted. Rather, by articulating what information must be transparently reported, they indirectly encourage better research practices and facilitate the assessment of adherence to contemporary methodological standards. For example, inadequate reporting in clinical trials may result in inflated confidence in the efficacy of an intervention. Similarly, insufficient detail in studies of reliability and agreement can lead to incorrect conclusions about measurement properties. In both cases, the downstream consequences include misguided clinical decision-making and reduced reproducibility. Over time, the widespread use of rigorously developed reporting guidelines, such as the GRRAS-COSMIN reporting guideline, has the potential to enhance research quality by fostering greater methodological awareness and transparency across disciplines.

**Limitations**

A comprehensive search strategy with no language restrictions will be employed across multiple databases to ensure thorough coverage of the literature. Nonetheless, this review has several methodological limitations that should be considered when interpreting the findings. First, the search strings are specific and focused, which may improve precision but could limit sensitivity and risk missing relevant literature. Specifically, this review seeks to identify methodological developments relevant to reliability, agreement, and measurement error in health, psychological or educational research and focuses on databases that primarily index health, psychological, and educational literature. Consequently, methodological developments published exclusively in, for example, mathematics or statistics journals may not be comprehensively captured.

Second, because the review includes diverse publication types such as methodological papers, tutorials, editorials, commentaries, guidance documents, and textbook material, and because the objective is to identify methodological developments rather than evaluate the validity of these studies, no formal critical appraisal of included sources will be undertaken. Consequently, the review will describe and synthesise methodological developments discussed in the literature but will not assess the relative merit or empirical support of competing methodological approaches.

Third, identifying and synthesising methodological developments requires a degree of interpretive judgement. This is inherent to qualitative synthesis [56]. We will address this through study selection being performed independently by two reviewers, documentation of coding decisions, reflexive discussions within the multidisciplinary author team, and transparent reporting of how themes are supported by the included sources.

Finally, while the absence of language restrictions may reduce the risk of language bias, non-English and non-German sources will be translated using automated translation tools. Consequently, inaccuracies in translation or subtle differences in terminology and meaning may affect the interpretation of methodological concepts, definitions, or recommendations. We will seek to minimise this risk by checking key passages against the original text where necessary.

For peer review only

## References

1 Kottner J, Audigé L, Brorson S, *et al.* Guidelines for Reporting Reliability and Agreement Studies (GRRAS) were proposed. *J Clin Epidemiol*. 2011;64:96–106. doi: 10.1016/j.jclinepi.2010.03.002

2 Mokkink LB, Eekhout I, Boers M, *et al.* Studies on Reliability and Measurement Error of Measurements in Medicine - From Design to Statistics Explained for Medical Researchers. *Patient Relat Outcome Meas*. 2023;14:193–212. doi: 10.2147/PROM.S398886

3 COSMIN definitions of domains, measurement properties, and aspects of measurement properties. https://cosmin.nl/wp-content/uploads/COSMIN-definitions-domains-measurement-properties.pdf (accessed 19 August 2025)

4 American Educational Research Association, American Psychological Association, National Council on Measurement in Education. *Standards for educational and psychological testing*. Washington, DC: American Educational Research Association 2014.

5 Kottner J, Audige L, Brorson S, *et al.* Guidelines for Reporting Reliability and Agreement Studies (GRRAS) were proposed. *Int J Nurs Stud*. 2011;48:661–71. doi: 10.1016/j.ijnurstu.2011.01.016

6 What is a reporting guideline? | EQUATOR Network. https://www.equator-network.org/about-us/what-is-a-reporting-guideline/ (accessed 3 June 2025)

7 Kottner J, Streiner DL. The difference between reliability and agreement. *J Clin Epidemiol*. 2011;64:701–2. doi: 10.1016/j.jclinepi.2010.12.001

8 Streiner DL. Statistics Commentary Series: Commentary #15—Reliability. *J Clin Psychopharmacol*. 2016;36:305. doi: 10.1097/JCP.0000000000000517

9 Kottner J, Streiner DL. Internal consistency and Cronbach's *α*: A comment on Beeckman et al. (2010). *Int J Nurs Stud*. 2010;47:926–8. doi: 10.1016/j.ijnurstu.2009.12.018

10 Vach W, Gerke O. Gwet's AC1 is not a substitute for Cohen's kappa – A comparison of basic properties. *MethodsX*. 2023;10:102212. doi: 10.1016/j.mex.2023.102212

11 Van Tiggelen H, Kottner J, Campbell K, *et al.* Measurement properties of classifications for skin tears: A systematic review. *Int J Nurs Stud*. 2020;110:103694. doi: 10.1016/j.ijnurstu.2020.103694

12 Cairney J, Streiner DL. Using relative improvement over chance (RIOC) to examine agreement between tests: Three case examples using studies of developmental coordination disorder (DCD) in children. *Res Dev Disabil*. 2011;32:87–92. doi: 10.1016/j.ridd.2010.09.001

13 de Vet H, Mokkink L, Terwee C, *et al.* Clinicians are right not to like Cohen's κ. *BMJ-Br Med J*. 2013;346. doi: 10.1136/bmj.f2125

For peer review only

14 Bourredjem A, Cardot H, Devilliers H. Asymptotic Confidence Interval, Sample Size Formulas and Comparison Test for the Agreement Intra-Class Correlation Coefficient in Inter-Rater Reliability Studies. *Stat Med*. 2024;43:5060–76. doi: 10.1002/sim.10217

15 Lu M-J, Zhong W-H, Liu Y-X, *et al.* Sample Size for Assessing Agreement between Two Methods of Measurement by Bland−Altman Method. *Int J Biostat*. 2016;12. doi: 10.1515/ijb-2015-0039

16 Hong H, Choi Y, Hahn S, *et al.* Nomogram for sample size calculation on a straightforward basis for the kappa statistic. *Ann Epidemiol*. 2014;24:673–80. doi: 10.1016/j.annepidem.2014.06.097

17 Mokkink LB, de Vet H, Diemeer S, *et al.* Sample size recommendations for studies on reliability and measurement error: an online application based on simulation studies. *Health Serv Outcomes Res Methodol*. 2023;23:241–65. doi: 10.1007/s10742-022-00293-9

18 Oster C, Schoo A, Litt J, *et al.* Supporting workforce practice change: protocol for a pilot study of a motivational interviewing virtual client software tool for health professionals. *BMJ OPEN*. 2020;10. doi: 10.1136/bmjopen-2019-033080

19 Gerke O, Möller S, Debrabant B, *et al.* Experience Applying the Guidelines for Reporting Reliability and Agreement Studies (GRRAS) Indicated Five Questions Should Be Addressed in the Planning Phase from a Statistical Point of View. *Diagnostics*. 2018;8:69. doi: 10.3390/diagnostics8040069

20 Gagnier J, de Arruda G, Terwee C, *et al.* COSMIN reporting guideline for studies on measurement properties of patient-reported outcome measures: version 2.0. *Qual LIFE Res*. 2025;34:1901–11. doi: 10.1007/s11136-025-03950-x

21 Elsman EBM, Mokkink LB, Terwee CB, *et al.* Guideline for reporting systematic reviews of outcome measurement instruments (OMIs): PRISMA-COSMIN for OMIs 2024. *J PATIENT-Rep OUTCOMES*. 2024;8. doi: 10.1186/s41687-024-00727-7

22 Mokkink LB, Elsman EBM, Terwee CB. COSMIN guideline for systematic reviews of patient-reported outcome measures version 2.0. *Qual Life Res*. 2024;33:2929–39. doi: 10.1007/s11136-024-03761-6

23 Mokkink LB, Boers M, Vleuten CPM van der, *et al.* COSMIN Risk of Bias tool to assess the quality of studies on reliability or measurement error of outcome measurement instruments: a Delphi study. *BMC Med Res Methodol*. 2020;20. doi: 10.1186/s12874-020-01179-5

24 Lucas NP, Macaskill P, Irwig L, *et al.* The development of a quality appraisal tool for studies of diagnostic reliability (QAREL). *J Clin Epidemiol*. 2010;63:854–61. doi: 10.1016/j.jclinepi.2009.10.002

25 Cohen JF, Korevaar DA, Altman DG, *et al.* STARD 2015 guidelines for reporting diagnostic accuracy studies: explanation and elaboration. *BMJ Open*. 2016;6:e012799. doi: 10.1136/bmjopen-2016-012799

For peer review only

For peer review only - http://bmjopen.bmj.com/site/about/guidelines.xhtml

26 Bossuyt PM, Reitsma JB, Bruns DE, *et al.* STARD 2015: an updated list of essential items for reporting diagnostic accuracy studies. *BMJ*. 2015;351:h5527. doi: 10.1136/bmj.h5527

27 Whiting PF, Rutjes AWS, Westwood ME, *et al.* QUADAS-2: a revised tool for the quality assessment of diagnostic accuracy studies. *Ann Intern Med*. 2011;155:529–36. doi: 10.7326/0003-4819-155-8-201110180-00009

28 Whiting P, Rutjes AW, Reitsma JB, *et al.* The development of QUADAS: a tool for the quality assessment of studies of diagnostic accuracy included in systematic reviews. *BMC Med Res Methodol*. 2003;3:25. doi: 10.1186/1471-2288-3-25

29 Moher D, Schulz KF, Simera I, *et al.* Guidance for Developers of Health Research Reporting Guidelines. *PLOS Med*. 2010;7:e1000217. doi: 10.1371/journal.pmed.1000217

30 Peters MDJ, Godfrey C, McInerney P, *et al.* Best practice guidance and reporting items for the development of scoping review protocols. *JBI Evid Synth*. 2022;20:953. doi: 10.11124/JBIES-21-00242

31 Clarke M. Guide to the contents of a Cochrane Methodology protocol and review.

32 Aguinis H, Ramani RS, Alabduljader N. Best-Practice Recommendations for Producers, Evaluators, and Users of Methodological Literature Reviews. *Organ Res Methods*. 2023;26:46–76. doi: 10.1177/1094428120943281

33 Hirt J, Ewald H, Briel M, *et al.* Searching a methods topic: practical challenges and implications for search design. *J Clin Epidemiol*. 2024;166:111201. doi: 10.1016/j.jclinepi.2023.10.017

34 Gentles SJ, Charles C, Nicholas DB, *et al.* Reviewing the research methods literature: principles and strategies illustrated by a systematic overview of sampling in qualitative research. *Syst Rev*. 2016;5:172. doi: 10.1186/s13643-016-0343-0

35 Tricco AC, Lillie E, Zarin W, *et al.* PRISMA Extension for Scoping Reviews (PRISMA-ScR): Checklist and Explanation. *Ann Intern Med*. 2018;169:467–73. doi: 10.7326/M18-0850

36 DeepL Privacy Policy | Protecting Your Privacy Is Important To Us. https://www.deepl.com/en/privacy (accessed 1 July 2026)

37 KI-Plattform von DeepL: Übersetzung, Voice und API. https://www.deepl.com (accessed 1 June 2026)

38 Vet HCW de, Mokkink LB, Terwee CB, *et al.* Clinicians are right not to like Cohen's κ. *BMJ*. 2013;346:f2125. doi: 10.1136/bmj.f2125

39 Clark J, Glasziou P, Del Mar C, *et al.* A full systematic review was completed in 2 weeks using automation tools: a case study. *J Clin Epidemiol*. 2020;121:81–90. doi: 10.1016/j.jclinepi.2020.01.008

For peer review only

40 Forbes C, Greenwood H, Carter M, *et al.* Automation of duplicate record detection for systematic reviews: Deduplicator. *Syst Rev*. 2024;13:206. doi: 10.1186/s13643-024-02619-9

41 Identify trusted publishers for your research • Think. Check. Submit. Think Check Submit. https://thinkchecksubmit.org/ (accessed 5 June 2026)

42 Organisation for Economic Co-operation and Development. OECD schema to Web of Science Category Mapping 2022.

43 MAXQDA Software für qualitative Datenanalyse. 1989.

44 Hsieh H-F, Shannon SE. Three approaches to qualitative content analysis. *Qual Health Res*. 2005;15:1277–88. doi: 10.1177/1049732305276687

45 Graneheim UH, Lundman B. Qualitative content analysis in nursing research: concepts, procedures and measures to achieve trustworthiness. *Nurse Educ Today*. 2004;24:105–12. doi: 10.1016/j.nedt.2003.10.001

46 10.2.8 Analysis of the evidence - JBI Manual for Evidence Synthesis - JBI Global Wiki. https://jbi-global-wiki.refined.site/space/MANUAL/355862791/10.2.8+Analysis+of+the+evidence (accessed 3 July 2025)

47 Braun V, and Clarke V. One size fits all? What counts as quality practice in (reflexive) thematic analysis? *Qual Res Psychol*. 2021;18:328–52. doi: 10.1080/14780887.2020.1769238

48 De Vet HCW, Terwee CB, Mokkink LB, *et al. Measurement in Medicine: A Practical Guide*. 1st ed. Cambridge University Press 2011.

49 Streiner DL, Kottner J. Recommendations for reporting the results of studies of instrument and scale development and testing. *J Adv Nurs*. 2014;70:1970–9. doi: 10.1111/jan.12402

50 Streiner DL, Norman GR, Cairney J. *Health Measurement Scales: A practical guide to their development and use*. Sixth Edition, Sixth Edition. Oxford, New York: Oxford University Press 2024.

51 Prinsen CAC, Vohra S, Rose MR, *et al.* How to select outcome measurement instruments for outcomes included in a “Core Outcome Set” – a practical guideline. *Trials*. 2016;17:449. doi: 10.1186/s13063-016-1555-2

52 Kottner J., Blume-Peytavi U. Reliability and agreement of instrumental skin barrier measurements in clinical pressure ulcer prevention research. *Int Wound J*. 2021;18:716 EP – 727. doi: 10.1111/iwj.13574

53 Importance of Transparent Reporting of Health Research. *Guidelines for Reporting Health Research: A User’s Manual*. Wiley 2014:1–13.

For peer review only

For peer review only - http://bmjopen.bmj.com/site/about/guidelines.xhtml

54 Simera I, Altman DG. Writing a research article that is “fit for purpose”: EQUATOR Network and reporting guidelines. *Ann Intern Med*. 2009;151:JC2-2. doi: 10.7326/0003-4819-151-4-200908180-02002

55 Using Reporting Guidelines Effectively to Ensure Good Reporting of Health Research. *Guidelines for Reporting Health Research: A User’s Manual*. Wiley 2014:32–40.

56 Field SM, Pownall M. Subjectivity is a Feature, not a Flaw: A Call to Unsilence the Human Element in Science. 2025.

**Author contributions:** D.L.S.: Funding acquisition, Methodology, and Writing - review & editing. J.K.: Conceptualization, Funding acquisition, Methodology, Project administration, Supervision, and Writing - review & editing. L.B.M.: Funding acquisition, Methodology, and Writing - review & editing. M.-M.P.: Conceptualization, Data curation, Methodology, Project administration, Resources, and Writing - original draft. M.M.: Data curation, Methodology, and Writing - review & editing. M.S.: Funding acquisition, Methodology, and Writing - review & editing. S.B.: Funding acquisition, Methodology, and Writing - review & editing. M.-M.P. is responsible for the overall content as guarantor.

**Funding statement:** The project is funded by the Deutsche Forschungsgemeinschaft (DFG, German Research Foundation) – 560535232.

**Competing Interest Statement:** The authors have no conflict of interest to declare.

**Acknowledgement:** We thank Corinna Dressler, PhD; Medical Library Charité-Universitätsmedizin Berlin for her support in the development of the search strategy.

For peer review only

**Supplement of: Developments in the field of reliability and agreement studies in health, psychological, and educational measurement: a methods review protocol**

Pittelkow, M.-M., Mönch, M., Mokkink, L.B., Streiner, D. L., Brorson S., Shoukri M., Kottner J.

This supplement details the sample references and search string development corresponding to review question 2 of the protocol "Developments in the field of reliability and agreement studies in health, psychological, and educational measurement: a methods review protocol
".

**Review question 2**: What major methodological discussions, recommendations, and/or advancements have been made in the last decade concerning the design, conduct, analysis, and reporting of reliability, and agreement/measurement error studies?

**Sample references**

This is a preliminary list of key references to be included in the scoping review in addition to the sources identified via the systematic search. Please note that this list is not exhaustive and sources may be added during the conduct of the review.

**Journal articles**

1. Borg, D. N., Bach, A. J. E., O'Brien, J. L., & Sainani, K. L. (2022). Calculating sample size for reliability studies. PM & R : The Journal of Injury, Function, and Rehabilitation, 14(8), 1018–1025. https://doi.org/10.1002/pmrj.12850
2. Cohen JF, Korevaar DA, Altman DG, Bruns DE, Gatsonis CA, Hooft L, Irwig L, Levine D, Reitsma JB, de Vet HC, Bossuyt PM. STARD 2015 guidelines for reporting diagnostic accuracy studies: explanation and elaboration. BMJ Open. 2016 Nov 14;6(11):e012799. https://doi.org/10.1136/bmjopen-2016-012799.
3. Koo, T. K., & Li, M. Y. (2016). A Guideline of Selecting and Reporting Intraclass Correlation Coefficients for Reliability Research. Journal of Chiropractic Medicine, 15(2), 155–163. https://doi.org/10.1016/j.jcm.2016.02.012
4. Mokkink, L. B., Boers, M., van der Vleuten, C. P. M., Bouter, L. M., Alonso, J., Patrick, D. L., de Vet, H. C. W., & Terwee, C. B. (2020). COSMIN Risk of Bias tool to assess the quality of studies on reliability or measurement error of outcome measurement instruments: A Delphi study. BMC Medical Research Methodology, 20(1), 293. https://doi.org/10.1186/s12874-020-01179-5
5. Mokkink, L. B., de Vet, H., Diemeer, S., & Eekhout, I. (2023). Sample size recommendations for studies on reliability and measurement error: An online application based on simulation studies.

For peer review only

Health Services and Outcomes Research Methodology, 23(3), 241–265. https://doi.org/10.1007/s10742-022-00293-9

6. Mokkink, L. B., Eekhout, I., Boers, M., van der Vleuten, C. P. M., & de Vet, H. C. W. (2023). Studies on Reliability and Measurement Error of Measurements in Medicine – From Design to Statistics Explained for Medical Researchers. Patient Related Outcome Measures, 14, 193–212. https://doi.org/10.2147/PROM.S398886
7. Oster, N. V., Carney, P. A., Allison, K. H., Weaver, D. L., Reisch, L. M., Longton, G., Onega, T., Pepe, M., Geller, B. M., Nelson, H. D., Ross, T. R., Tosteson, A. N. A., & Elmore, J. G. (2013). Development of a diagnostic test set to assess agreement in breast pathology: Practical application of the Guidelines for Reporting Reliability and Agreement Studies (GRRAS). BMC Women's Health, 13(1), 3. https://doi.org/10.1186/1472-6874-13-3
8. Stratford, P., Spadoni, G. F., Kuspinar, A., & Macedo, L. (2025). Considerations when designing, analyzing, and reporting reliability studies. Brazilian Journal of Physical Therapy, 29(3), 101193. https://doi.org/10.1016/j.bjpt.2025.101193
9. Vach, W., & Gerke, O. (2023). Gwet's AC1 is not a substitute for Cohen's kappa – A comparison of basic properties. MethodsX, 10, 102212. https://doi.org/10.1016/j.mex.2023.102212
10. Vet, H. C. W. de, Mokkink, L. B., Terwee, C. B., Hoekstra, O. S., & Knol, D. L. (2013). Clinicians are right not to like Cohen's κ. BMJ, 346, f2125. https://doi.org/10.1136/bmj.f2125
11. Tran, D., Dolgun, A., & Demirhan, H. (2020). Weighted inter-rater agreement measures for ordinal outcomes. *Communications in Statistics - Simulation and Computation*, *49*(4), 989–1003. https://doi.org/10.1080/03610918.2018.1490428
12. ten Hove, D., Jorgensen, T. D., & van der Ark, L. A. (2024). Updated guidelines on selecting an intraclass correlation coefficient for interrater reliability, with applications to incomplete observational designs. Psychological Methods, 29(5), 967–979. https://doi.org/10.1037/met0000516

**Textbooks**

13. De Vet, H. C., Terwee, C. B., Mokkink, L. B., & Knol, D. L. (2011). *Measurement in medicine: a practical guide*. Cambridge university press.
14. American Educational Research Association, American Psychological Association, & National Council on Measurement in Education. (2014). Standards for educational and psychological testing. American Educational Research Association.

For peer review only

15. Streiner, D. L., Norman, G. R., & Cairney, J. (2024). *Health Measurement Scales: A practical guide to their development and use* (Sixth Edition, Sixth Edition). Oxford University Press.

**Manual**

16. Mokkink, L. B., E. Elsman, C.B. Terwee. COSMIN guideline for systematic reviews of Patient-Reported Outcome Measures version 2.0. Qual Life Res (2024). https://doi.org/10.1007/s11136-024-03761-6.

For peer review only

## Search String Development

The search string was developed with both sensitivity (maximizing the identification of relevant articles) and feasibility (retrieving a manageable number of records for screening by two reviewers) in mind. Finding this balance can be especially challenging for reviews of methods topics [1]. We started by compiling a reference sample based on PubMed IDs of key journal articles (i.e., 28137831, 27330520, 37448975, 23379630, 27464308, 40022968, 37234937, 23585065, 33267819, 35596122). These articles serve as examples of the types of methodological literature we aim to identify and are not necessarily expected to be fully captured by the final search string. For example, we decided a priori to limit the search to publications from the last decade (1.1.2015 to 30.6.2025), since our aim is to cover recent methodological discussions and advancements. As a result, some of the articles included in the reference sample may not appear in the search output due to their earlier publication date.

In a first step, we identified relevant search terms and Medical Subject Headings (MeSH) terms. To this end, we (1) used the Systematic Review Accelerator (SR-Accelerator) [2] to extract and review frequently used words in the key references (see table S1) and (2) reviewed the assigned MeSH terms for the reference sample using the Yale Mesh Analyzer (1, see table S2). We additionally consulted the term finder in Ovid for MeSH terms related to reliability, agreement, or measurement error as well as the Thesaurus for Health Research Methods from the Library of Guidance for Health Scientists [4].

*Table S1. Word frequencies in title, abstract, and keywords of key references.*

| word | unique | title | abstract | keywords | points |
|---|---|---|---|---|---|
| reliability | 6 | 5 | 18 | 0 | 23 |
| agreement | 4 | 3 | 18 | 0 | 21 |
| measurement | 3 | 4 | 12 | 0 | 16 |
| study | 3 | 1 | 14 | 0 | 15 |
| icc | 2 | 0 | 14 | 0 | 14 |
| studies | 5 | 6 | 7 | 0 | 13 |
| between | 5 | 1 | 10 | 0 | 11 |
| cohen's | 2 | 1 | 9 | 0 | 10 |
| reporting | 4 | 4 | 6 | 0 | 10 |
| methods | 3 | 1 | 8 | 0 | 9 |
| kappa | 1 | 1 | 8 | 0 | 9 |
| ac1 | 1 | 1 | 8 | 0 | 9 |
| design | 4 | 0 | 9 | 0 | 9 |
| gwet's ac1 | 1 | 0 | 8 | 0 | 8 |
| gwet's | 1 | 0 | 8 | 0 | 8 |
| error | 2 | 2 | 6 | 0 | 8 |
| measure | 1 | 0 | 7 | 0 | 7 |
| outcome measurement | 1 | 1 | 6 | 0 | 7 |
| outcome | 1 | 1 | 6 | 0 | 7 |
| cohen's kappa | 1 | 0 | 7 | 0 | 7 |
| measurements | 2 | 0 | 7 | 0 | 7 |
| form | 1 | 0 | 6 | 0 | 6 |
| values | 4 | 0 | 6 | 0 | 6 |
| test | 4 | 2 | 4 | 0 | 6 |
| repeated | 2 | 0 | 6 | 0 | 6 |
| measurement error | 2 | 2 | 4 | 0 | 6 |
| size | 2 | 1 | 5 | 0 | 6 |
| word | unique | title | abstract | keywords | points |
| sample | 2 | 1 | 5 | 0 | 6 |
| when | 3 | 1 | 5 | 0 | 6 |
| κ | 1 | 1 | 4 | 0 | 5 |
| article | 1 | 0 | 5 | 0 | 5 |
| instrument | 1 | 0 | 5 | 0 | 5 |
| assess | 2 | 2 | 3 | 0 | 5 |
| rate | 1 | 0 | 5 | 0 | 5 |

For peer review only

| | | | | | |
|---|---|---|---|---|---|
| researchers | 3 | 0 | 5 | 0 | 5 |
| ratings | 3 | 0 | 5 | 0 | 5 |
| different | 4 | 0 | 5 | 0 | 5 |
| scores | 2 | 0 | 5 | 0 | 5 |
| correlation | 2 | 1 | 4 | 0 | 5 |
| e | 3 | 0 | 5 | 0 | 5 |
| based | 3 | 1 | 4 | 0 | 5 |
| raters | 4 | 0 | 5 | 0 | 5 |
| information | 4 | 1 | 4 | 0 | 5 |
| results | 3 | 0 | 5 | 0 | 5 |
| sample size | 2 | 1 | 4 | 0 | 5 |
| purpose | 1 | 0 | 5 | 0 | 5 |
| quantity | 1 | 0 | 4 | 0 | 4 |
| clinicians | 1 | 1 | 3 | 0 | 4 |
| diagnostic | 2 | 2 | 2 | 0 | 4 |
| research | 3 | 1 | 3 | 0 | 4 |
| reached | 1 | 0 | 4 | 0 | 4 |
| consensus | 1 | 0 | 4 | 0 | 4 |
| variation | 2 | 0 | 4 | 0 | 4 |
| how | 1 | 0 | 4 | 0 | 4 |

| word | unique | title | abstract | keywords | points |
|---|---|---|---|---|---|
| measurement instruments | 1 | 1 | 3 | 0 | 4 |
| instruments | 1 | 1 | 3 | 0 | 4 |
| quality | 1 | 1 | 3 | 0 | 4 |
| agreement rate | 1 | 0 | 4 | 0 | 4 |
| positive | 2 | 0 | 4 | 0 | 4 |
| properties | 2 | 1 | 3 | 0 | 4 |
| developed | 3 | 0 | 4 | 0 | 4 |
| k | 1 | 0 | 4 | 0 | 4 |
| repeated measurements | 1 | 0 | 4 | 0 | 4 |
| patients | 2 | 0 | 4 | 0 | 4 |
| application | 2 | 2 | 2 | 0 | 4 |
| calculation | 3 | 0 | 4 | 0 | 4 |
| discussion | 3 | 0 | 4 | 0 | 4 |
| provide | 3 | 0 | 4 | 0 | 4 |
| study purpose | 1 | 0 | 4 | 0 | 4 |

| | | | | | |
|---|---|---|---|---|---|
| however | 4 | 0 | 4 | 0 | 4 |
| observations | 1 | 1 | 2 | 0 | 3 |
| same | 2 | 0 | 3 | 0 | 3 |
| cohen’s κ | 1 | 1 | 2 | 0 | 3 |
| readers | 1 | 0 | 3 | 0 | 3 |
| appropriate | 2 | 0 | 3 | 0 | 3 |
| icc form | 1 | 0 | 3 | 0 | 3 |
| guidelines | 2 | 2 | 1 | 0 | 3 |
| breast | 1 | 1 | 2 | 0 | 3 |
| diagnostic test | 2 | 2 | 1 | 0 | 3 |

| word | unique | title | abstract | keywords | points |
|---|---|---|---|---|---|
| reported | 3 | 0 | 3 | 0 | 3 |
| standards | 1 | 0 | 3 | 0 | 3 |
| measurement instrument | 1 | 0 | 3 | 0 | 3 |
| tool | 2 | 1 | 2 | 0 | 3 |
| both | 2 | 0 | 3 | 0 | 3 |
| paper | 3 | 0 | 3 | 0 | 3 |
| using | 3 | 0 | 3 | 0 | 3 |
| agreement between | 2 | 1 | 2 | 0 | 3 |
| basic | 2 | 1 | 2 | 0 | 3 |
| most | 2 | 0 | 3 | 0 | 3 |
| mse | 1 | 0 | 3 | 0 | 3 |
| parameters | 2 | 0 | 3 | 0 | 3 |
| about | 3 | 0 | 3 | 0 | 3 |
| intraclass correlation | 2 | 1 | 2 | 0 | 3 |
| intraclass | 2 | 1 | 2 | 0 | 3 |
| number | 2 | 0 | 3 | 0 | 3 |
| online application | 1 | 1 | 2 | 0 | 3 |
| online | 1 | 1 | 2 | 0 | 3 |
| whether | 2 | 0 | 3 | 0 | 3 |
| reliability study | 3 | 0 | 3 | 0 | 3 |
| reliability studies | 2 | 1 | 2 | 0 | 3 |
| reporting reliability | 2 | 2 | 1 | 0 | 3 |

For peer review only

Table S2. Results from the Yale MeSH Analyzer

| **PMID** | 28137831 | 27330520 | 37448975 | 23379630 | 27464308 | 40022968 | 37234937 | 23585065 | 33267819 |
|---|---|---|---|---|---|---|---|---|---|
| **MeSH Headings** | Advisory Committees* | | | | | | | | |
| | Bias<br>Biomedical Research | | | Breast / pathology<br>Breast Diseases / pathology*<br>Breast Neoplasms / classification*<br>Breast Neoplasms / diagnosis<br>Breast Neoplasms / pathology* | | | | | Bias |
| | Checklist* | | | | | | | | Consensus |
| | Diagnostic Techniques and Procedures / standards* | | | Diagnosis, Differential | Diagnostic Tests, Routine / standards* | | | | Delphi Technique* |
| | | | | Female | | | | | |
| | | | | | Guidelines as Topic | | | | |
| | Humans | | | Humans | Humans | Humans | | Humans | Humans |
| | | | | Observer Variation | | | | Observer Variation* | |

(continued)

For peer review only

Table S2 continued. Results from the Yale MeSH Analyzer

| **PMID** | 28137831 | 27330520 | 37448975 | 23379630 | 27464308 | 40022968 | 37234937 | 23585065 | 33267819 |
|---|---|---|---|---|---|---|---|---|---|
| | Publishing | | | Predictive Value of Tests | Psychology / standards<br>Psychometrics / standards*<br>Publishing / standards* | | | Probability* | |
| | | | | Quality Assurance, Health Care / standards* | | | | | |
| | Research Design | | | Reproducibility of Results<br>Research Design / standards | | Reproducibility of Results<br>Research Design* | | Reproducibility of Results*<br>Research Design* | Reproducibility of Results |
| | | | | Sensitivity and Specificity | Societies, Scientific | Sample Size | | | |
| | Terminology as Topic | | | | | | | | |
| **Author Assigned Keywords** | Diagnostic accuracy<br>Medical publishing<br>Peer review<br>Reporting quality<br>Research waste<br>Sensitivity and specificity | Reliability and validity<br>Research<br>Statistics | classical test theory<br>generalizability theory<br>intraclass correlation coefficient<br>measurement error<br>reliability<br>standard error of measurement | | | Measurement Methods<br>Rehabilitation<br>Reliability<br>Research design<br>Statistics | Agreement<br>Gwet's AC1<br>Method comparison<br>Observer<br>Rater<br>Reliability<br>Repeatability<br>Reproducibility | | COSMIN<br>Delphi study<br>Measurement error<br>Outcome measurement instruments<br>Quality assessment<br>Reliability<br>Risk of Bias |

For peer review only

From this, we constructed a set of possible search and MeSH terms (see table S3).

**Table S3. Potential search and MeSH terms. MeSH terms are retrieved from Medline via Ovid**

| Search concept | Potential search terms | |
|---|---|---|
| **Target:**<br><br>**Methodological studies, reviews, commentaries, editorials, and guidance documents that explicitly discuss, evaluate, reflect on, or propose methodological aspects of reliability, agreement, and/or measurement error.** | Reliability | Method* |
| | reproducibility of measurements | Outcome |
| | Agreement | Cohen's |
| | Bland Altmann analysis | Kappa |
| | Measurement | ICC |
| | Measurement error | AC1 |
| | Measure* | gwet's |
| | Stud* | Guidance |
| | Reporting | How to |
| | Reporting quality | Best practice |
| | MeSH: Research Design / standards | MeSH: Statistics as Topic |
| | MeSH: Reproducibility of Results | MeSH: Reproducibility of Results |
| | MeSH: Publishing / standards* | MeSH: Methods / Research Design |

We then iteratively piloted the search terms in Medline via OVID to develop a combination that was both sensitive and resulted in a feasible number of hits. To check the sensitivity of our search, we searched for the reference sample in the hits of the search string before adding the publication date restriction.

## References


1 Hirt J, Ewald H, Briel M, *et al.* Searching a methods topic: practical challenges and implications for search design. *J Clin Epidemiol*. 2024;166:111201. doi: 10.1016/j.jclinepi.2023.10.017

2 Clark J, Glasziou P, Del Mar C, *et al.* A full systematic review was completed in 2 weeks using automation tools: a case study. *J Clin Epidemiol*. 2020;121:81–90. doi: 10.1016/j.jclinepi.2020.01.008

3 Yale MeSH Analyzer. https://mesh.med.yale.edu/ (accessed 23 July 2025)

4 Hirt J, Schönenberger CM, Ewald H, *et al.* Introducing the Library of Guidance for Health Scientists (LIGHTS): A Living Database for Methods Guidance. *JAMA Netw Open*. 2023;6:e2253198. doi: 10.1001/jamanetworkopen.2022.53198


For peer review only
